# Selective Ion Binding and Uptake Shape the Microenvironment of Biomolecular Condensates

**DOI:** 10.1021/jacs.5c07295

**Published:** 2025-07-14

**Authors:** Iris B. A. Smokers, Enrico Lavagna, Rafael V. M. Freire, Matteo Paloni, Ilja K. Voets, Alessandro Barducci, Paul B. White, Mazdak Khajehpour, Evan Spruijt

**Affiliations:** † Institute for Molecules and Materials, 6029 Radboud University, Heyendaalseweg 135, 6525 AJ Nijmegen, The Netherlands; ‡ Centre de Biologie Structurale, Université de Montpellier, CNRS, INSERM, Montpellier 34090, France; § Laboratory of Self-Organizing Soft Matter, Department of Chemical Engineering and Chemistry, 3169 Eindhoven University of Technology, P.O. Box 513, 5600 MB Eindhoven, The Netherlands; ∥ Institute for Complex Molecular Systems, Eindhoven University of Technology, P.O. Box 513, 5600 MB Eindhoven, The Netherlands; ⊥ Thomas Young Centre and Department of Chemical Engineering, 4919 University College London, London WC1E 7JE, United Kingdom; # Department of Chemistry, University of Manitoba, Winnipeg, Manitoba R3T 2N2, Canada

## Abstract

Biomolecular condensates modulate various ion-dependent cellular processes and can regulate subcellular ion distributions by selective uptake of ions. To understand these processes, it is essential to uncover the molecular grammar governing condensate-ion interactions. Here, we use nuclear magnetic resonance (NMR) spectroscopy of ions and model condensate components to quantify and spatially resolve selective ion binding to condensates and show that these interactions follow the “law of matching water affinities”, resulting in strong binding between proteins and chaotropic anions and between nucleic acids and kosmotropic cations. Ion uptake into condensates directly follows binding affinities, resulting in selective uptake of strong-binding ions but exclusion of weak-binding ions. Ion binding further shapes the condensate microenvironment by altering the composition, viscosity, and interface potential. Such changes can have profound effects on biochemical processes taking place inside condensates, as we show for RNA duplex formation. Our findings provide a new perspective on the role of condensate-ion interactions in cellular bio- and electrochemistry and may aid the design of condensate-targeting therapeutics.

## Graphical abstract

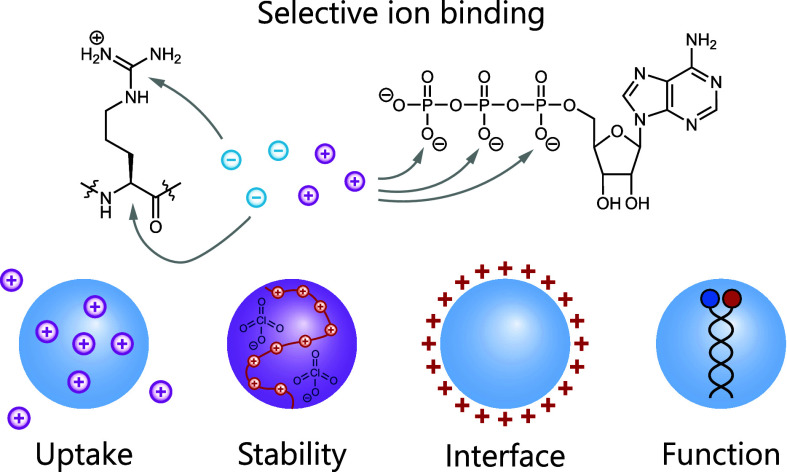

## 1 Introduction

Biomolecular condensates are phase-separated compartments that regulate a variety of cellular processes, including ribosome biogenesis, stress response, and protein aggregation.^1,2^ They are formed through multivalent interactions between proteins and, in some cases, RNA, resulting in droplets that are enriched in biomolecules and have distinct local physicochemical environments. These environments can differ in hydrophobicity, viscosity, and the presence of specific ions, and drastically alter biochemical processes.^3,4^ By uptake of divalent metal ions such as Mg^2+^ and Cu^2+^, condensates regulate both ribozyme function^5,6^ and protein aggregation,^2^ and could alter the reactivity of enzymes that require metal ions in their active site.^7–11^ In addition, the differential uptake of ions into condensates has recently been shown to modulate subcellular ion distributions and may thereby play a role in regulating the electrochemistry of cells.^12–14^ Outside the cell, condensates can also be used to remove heavy metals and other ions from wastewater.^15–17^

Ions also modulate the stability of condensates. Condensate formation is driven by supramolecular interactions such as charge–charge, π–π, and cation–π interactions and the hydrophobic effect, and the release of counterions and hydration water makes their formation entropically favorable.^18–21^ This makes condensates susceptible to salt, as ions screen charges,^22,23^ can give rise to re-entrant phase separation^24^ and can modulate the hydrophobic effect to salt in or salt out proteins following the Hofmeister series.^25–28^ Several marine species use condensates as underwater adhesives^29,30^ and extracellular hard tissues,^31,32^ and exploit the exposure to seawater to alter the physical state of the condensates from liquid to gel-like. Furthermore, condensates are suggested to have played a role in the origins of life as a first generation of cell-like compartments^33–35^ —or protocells—in the salty prebiotic soup.^36,37^

These striking effects of ions on condensates have sparked a growing interest in measuring ion uptake in condensates.^14,23,28,38–51^ However, the molecular mechanisms behind interactions of ions with condensates require further investigation. Binding of ions to specific regions in the condensate components could alter the conformation of biomolecules inside condensates, lead to selective uptake of ions and charged cofactors and therapeutics, and create distinct nanoscopic environments that foster specific reactions and alter condensate material properties. Therefore, insight into the molecular “grammar” of condensate-ion interactions is essential for a full understanding of condensate function and the potential of novel condensate-targeting therapeutics.^52–54^

In this work, we investigate the molecular mechanisms by which salt ions interact with the components of model condensates and how they affect the local environment inside the droplets. We investigate a range of ions with different properties: from weakly hydrated soft ions (chaotropes) to strongly hydrated hard ions (kosmotropes). Through NMR binding assays, we find that chaotropic anions and kosmotropic cations selectively bind to the condensate components, following the “law of matching water affinities” (LMWA), a trend that we postulate is universal for all condensates. Molecular dynamics simulations and SAXS measurements further show that the binding of ions to the peptide is sequence-specific and results in compaction of the peptide chain. Using a combination of ^1^H, ^7^Li, ^13^C, ^19^F, ^23^Na, ^25^Mg, ^31^P, ^35^Cl, ^39^K, ^81^Br, and ^133^Cs-NMR spectroscopy, we can quantify the full composition of the condensate phase with a single technique and show that the partitioning of ions directly follows their binding strength, resulting in preferential uptake of strong-binding ions but exclusion of weak-binding ions, in contrast to theoretical predictions.^55,56^

The binding and uptake of ions have a drastic effect on the properties of the condensates. Strong-binding ions remodel the phase diagram by effectively neutralizing charges and favoring other interactions such as π-π stacking, which can result in re-entrant phase separation. Selective ion binding further modulates the condensate viscosity and can flip the interface potential of condensates, which can impact their interaction with membranes and other intracellular structures. Lastly, we show that the altered microenvironment can affect biochemistry inside the condensates: kosmotropic cations can significantly stabilize RNA duplexes inside condensates, whereas the same ions destabilize RNA duplexes in solution. Taken together, our results show that ions can affect the condensate composition and microenvironment in dramatic and nontrivial manners, and we provide molecular insight into how specific ions bind selectively to condensates to shape their properties.

## 2 Results

### 2.1 Selective Binding of Ions to Condensate Components

Biomolecular condensates are composed of proteins that often contain significant disordered regions, and nucleic acids. To enable a systematic and high-resolution investigation of the specific binding of different salt ions to these condensate components, we used a peptide/nucleotide condensate model system, made with 1 mM protamine chloride (typical sequence H-PRRRRSSSRPIRRRRPRRASRRRRRRGGRRRR-OH)/25 mM sodium adenosine 5′-triphosphate (ATP) (Figure 1a,b, see Supporting Information Section 2.1 and 2.2 for a justification of the choice of model system and concentrations).^57–61^ We prepared condensates with a series of different anions and cations (100 mM for salts of monovalent ions and 50 mM for salts containing divalent ions to obtain equal concentrations of positive and negative charge for all salts, Figure 1a and Supporting Figure 2) ranging from strongly kosmotropic (hard, strongly hydrated) to strongly chaotropic (soft, weakly hydrated, Figure 1i). Ions can be classified according to their size and charge density, or “hardness”, which is linked to their hydration strength, because charge–charge interactions in water are mediated by water. This is one of the factors reflected in the Hofmeister series.^25^

**Figure 1:**
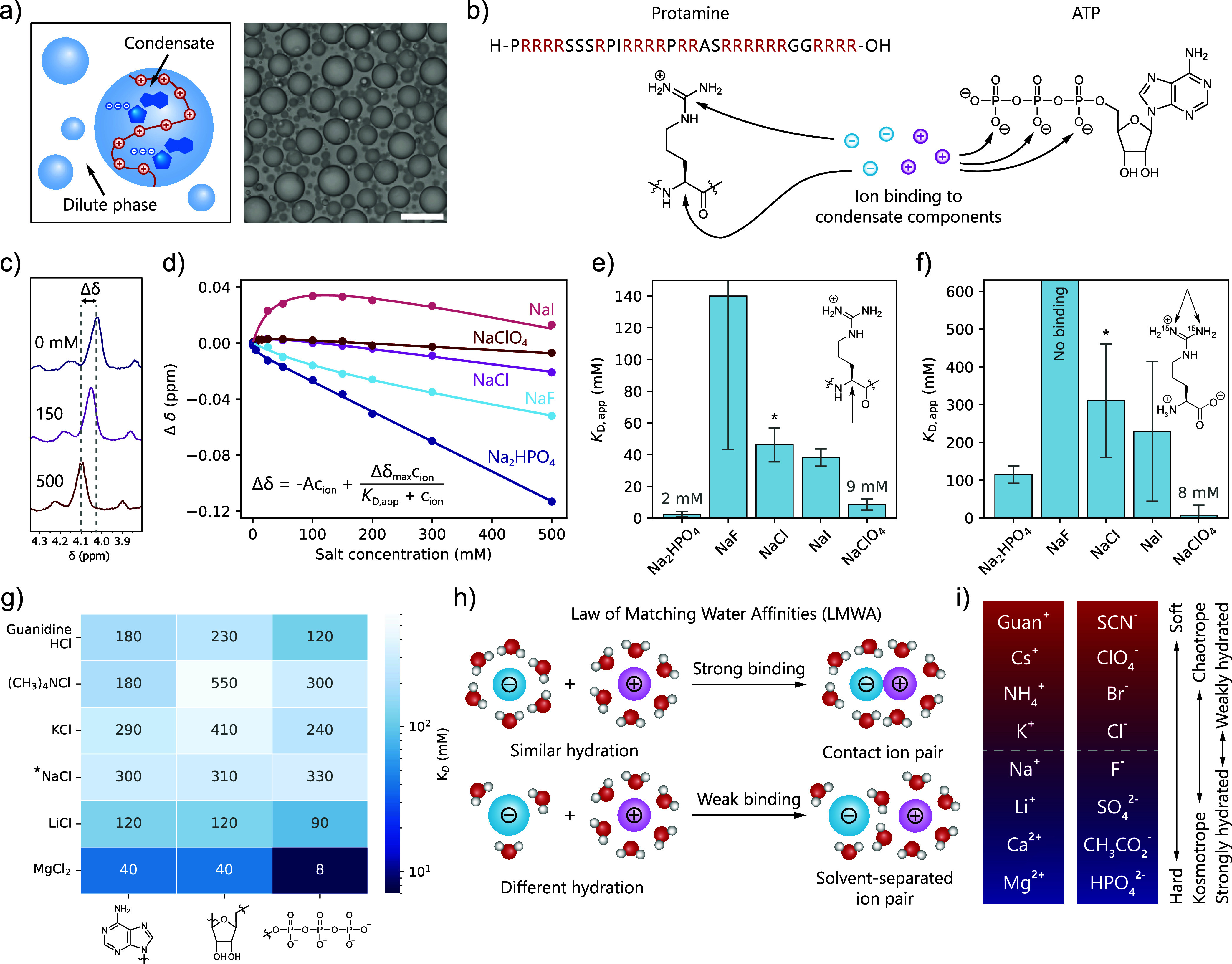
Ion binding to the condensate components protamine and ATP follows the “law of matching water affinities” (LMWA) and valency. (a) The arginine-rich peptide protamine and ATP form model biomolecular condensates; scale bar = 20 μm. (b) Through NMR-based binding assays, we investigated the binding of ions to the condensate components protamine and ATP in the absence of condensates. (c) ^1^H NMR chemical shifts of the arginine α-proton as a function of salt concentration at 5 °C. (d) Change in chemical shift (Δδ) as a function of salt concentration for different anions, fitted to a binding isotherm from Rembert et al.^62^ to obtain the apparent dissociation constant *K*_D,app_. The other binding curves can be found in Supporting Information Section 3.2. (e) Apparent dissociation constants of anions for the α-proton. Error bars represent the error of the fit. *Because the sample already contains 23.1 mM Cl^–^ at 0 mM added salt, this amount was added to the *K*_D,app_. (f) Apparent dissociation constants of anions for the guanidinium groups in arginine. Chaotropic and divalent anions bind strongly to the arginines in protamine. Error bars represent the error of the fit. *Because the sample already contains 61.8 mM Cl^–^ at 0 mM added salt, this amount was added to the *K*_D,app_. (g) Apparent dissociation constants of cations for the phosphate, ribose, and nucleobase of ATP. Kosmotropic and divalent cations bind strongly to the phosphates on ATP. *Because the sample already contains 92.9 mM Na^+^ at 0 mM added salt, this amount was added to the *K*_D,app_. (h) Binding of ions to the condensate components follows the “law of matching water affinities” (LMWA). (i) Trends in ion hardness, kosmo-/chaotropicity, and hydration for cations and anions.

We quantified the interaction strength between the ions and the individual condensate components in dilute solution using an NMR-based binding assay (Figure 1b), which was previously used in the literature to quantify binding strengths of ions to peptides (see Supporting Information Section 2.3.5 for the rationale for the choice of binding model).^62–65^ We measured the change in ^1^H, ^15^N, or ^31^P NMR chemical shift as a function of salt concentration (Figure 1c,d, and Supporting Information Section 3.2) to determine ion binding to the α-proton and guanidinium moiety of the arginines in protamine and to the phosphates, sugar, and nucleobase in ATP. Equation 1 describes the effect of ions on the NMR chemical shift: a linear term accounts for the nonspecific shift due to changes in the bulk solvent properties, such as an increase in ionic strength and change in water activity, and a Langmuir isotherm accounts for specific ion binding

1 $$\Delta \delta =-Ac_{\mathrm{ion}}+\frac{\Delta \delta_{\max}c_{\mathrm{ion}}}{K_{D,\mathrm{app}}+c_{\mathrm{ion}}}$$

In this equation, Δδ is the change in chemical shift in ppm with respect to the sample without added salt, *A* is the slope of the linear part with units of ppm/mM, *c*_ion_ is the concentration of the salt ion of interest in mM, Δδ_max_ is the maximum chemical shift change in ppm due to binding at saturation, and *K*_D,app_ is the apparent dissociation constant in mM. *A*, Δδ_max_ and *K*_D,app_ were obtained by fitting.

Using this method for sodium salts with different anions, we found that chaotropic anions such as ClO_4_^–^ bind surprisingly strongly to both the α-protons and guanidiniums of arginines, with low-mM apparent dissociation constants in both cases, while kosmotropic anions such as F^–^ do not bind or only bind weakly (Figure 1e,f, Supporting Information Sections 3.2.1 and 3.2.2). The strongly kosmotropic HPO_4_^2–^, however, binds to both the α-proton and the guanidinium, indicating that valency and possibly hydrogen bonding^66,67^ also play a role in binding strength. It is interesting to note that different ions have different signs and magnitudes of both the linear and nonlinear part of Δδ. While differences in the linear part may be explained by ion-specific effects on, e.g., water structuring and activity,^62,63^ differences in the sign of the nonlinear part suggest that the ions have different mechanisms of binding, which differentially affect the degree of shielding (Supporting Figures 4 and 6).

Contrary to the binding of chaotropic anions to protamine, for ATP, we observe that kosmotropic cations such as Mg^2+^ and Li^+^ bind strongly to the phosphates with low-mM *K*_D,app_ for Mg^2+^ (Figure 1g, and Supporting Information Section 3.2.3), while the weakly kosmotropic Na^+^, and chaotropic K^+^ and (CH_3_)_4_N^+^ have much weaker binding. Interestingly, for Mg^2+^, we observe two distinct populations of the β-phosphate between 5 and 20 mM MgCl_2_ (Supporting Figure 8), representing a bound and unbound state, indicating a slow release rate *k*_off_ for the Mg^2+^-ATP complex.^68^ The observed binding of Mg^2+^ and Li^+^ to the ribose and nucleobase is most likely due to proximity effects of the phosphates. We also observe binding for the strongly chaotropic guanidinium, likely because of hydrogen-bonding interactions.^66^

The binding assay shows that chaotropic monovalent anions interact strongly with the arginines in protamine, which are also chaotropic, while kosmotropic cations interact strongly with the phosphates on ATP, which are also kosmotropic. These observations follow the empirical “law of matching water affinities” (LMWA), as proposed by Collins.^69,70^ Because water has a high dielectric constant, long-range electrostatic forces disappear for ions in water. In these regimes, short-range effects such as the water affinity of ions become more pronounced. According to the LMWA, the water affinity, or hydration enthalpy, of ions predicts their interaction strength with other ions. Oppositely charged ions with matching water affinities interact most strongly (Figure 1h): two kosmotropes form a contact ion pair because of strong electrostatic attraction due to their high charge density, which can expel their strongly bound hydration water. Two chaotropes, on the other hand, have weaker electrostatic attraction, but form a contact ion pair to release unfavorably bound hydration water.^70^ Interaction between a kosmotrope and a chaotrope, however, is less favorable because the relatively weak electrostatic attraction cannot expel the kosmotrope’s hydration shell, and these combinations tend to form weaker solvent-separated ion pairs.^71^ We can see that the ability of matching water affinities to explain ion-interaction strengths for both kosmotropes and chaotropes is because water affinity is directly linked to ion hardness and thus the strength of electrostatic interactions, in addition to describing the strength of ion–water interactions.

Interestingly, all organic cations in nature (i.e., guanidinium and ammonium) are chaotropic, while all organic anions (i.e., phosphates, carboxylic acids, and sulfates) are kosmotropic (Figure 1i). Therefore, any protein in the condensate with positive charges is expected to bind chaotropic anions such as ClO_4_^–^, I^–^, and Br^–^, and any negatively charged protein or nucleic acid is expected to bind kosmotropic cations such as Li^+^ and Mg^2+^. The valency of the ions also influences their binding to condensate components: multivalent, kosmotropic phosphates do bind to the chaotropic arginines in protamine. Further analysis of ion binding to the squid-beak protein-derived hydrophobic peptide (GHGLY)_3_ ^31^ underlines the generality of these binding trends (Supporting Information Section 3.2.4).

### 2.2 Ion Binding Is Sequence-Specific and Compacts Protamine

To obtain deeper insight into the ion binding to condensate components, we ran all-atom explicit solvent molecular dynamics (MD) simulations of protamine with different anions. Radial distribution functions (Figure 2a,b) confirm that ClO_4_^–^ and Cl^–^ bind to the backbone α-protons and amide NHs, and to the guanidinium groups of arginines in protamine, with an apparent *K*_D_ that is in qualitative agreement with the NMR binding assays (Figure 2c, and Supporting Figures 15–16), while F^–^ shows no significant backbone binding.

**Figure 2:**
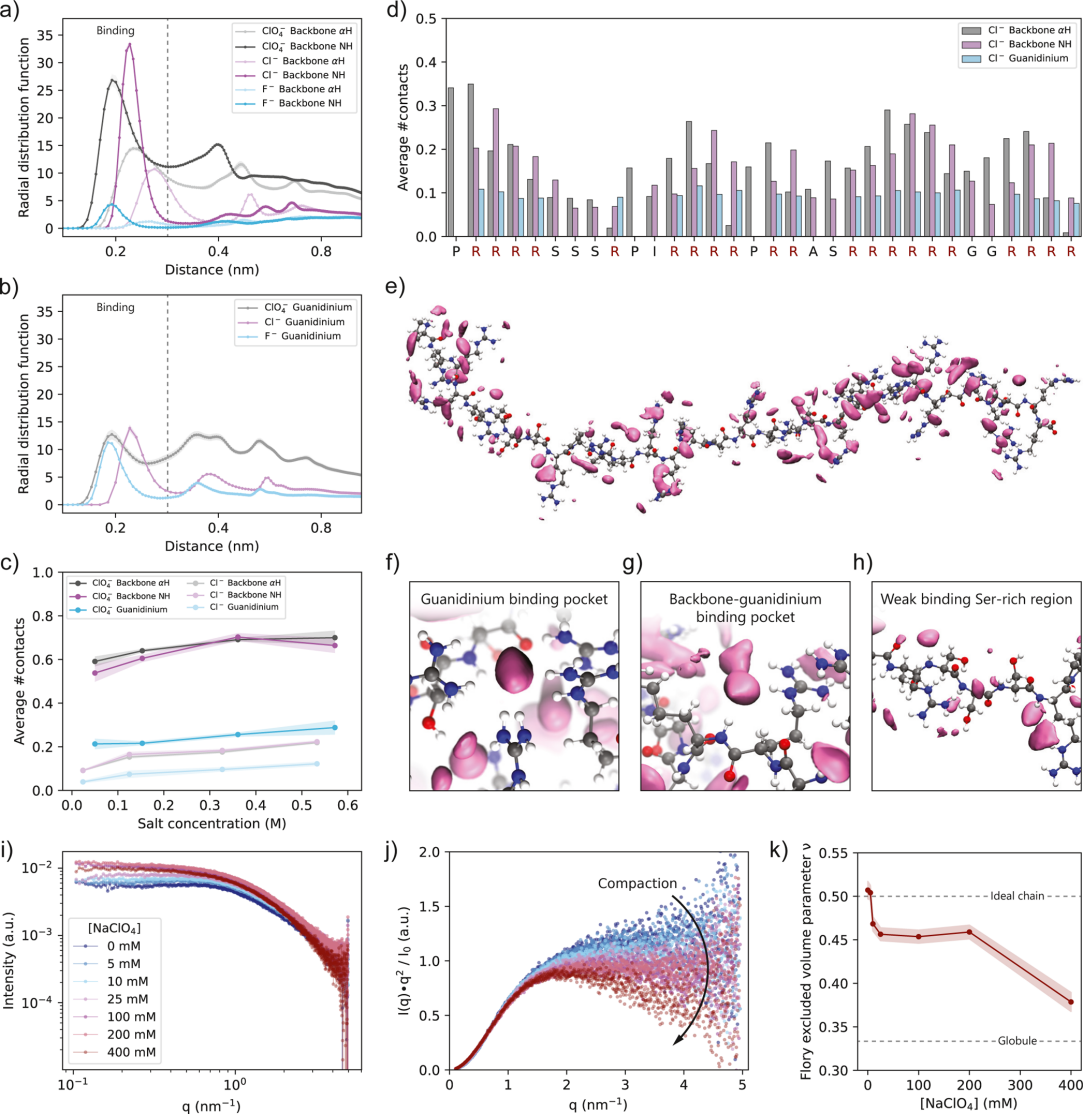
Ion binding to protamine is sequence-specific and causes compaction of the peptide chain. (a, b) Radial distribution functions (RDF) of NaF, NaCl, and NaClO_4_^–^ with respect to (a) the arginine backbone α-proton (αH) and NH and (b) guanidinium as obtained from all-atom explicit solvent Molecular Dynamics (MD) simulations at 300 mM added salt concentration. Dotted lines represent the cutoff radius for binding, which differs slightly per type of ion. (c) Average number of protamine hydrogen-ion contacts as a function of the ion concentration. (d) Average number of hydrogen-ion contacts per residue in protamine for 300 mM NaCl. (e) Snapshot of the whole protamine sequence with highlighted regions of high ion density from a simulation with the protamine constrained in the open configuration at 500 mM added NaCl concentration. The mauve solids represent isosurfaces with isovalue 40× the bulk ion concentration. The peptide is represented with white hydrogen, gray carbon, blue nitrogen, and red oxygen atoms. (f–h) Snapshots showing the binding pockets with high affinity for ions and the Ser-rich region with low affinity for ions. Snapshots are taken from simulations with the protamine constrained in the closed (f) or open configuration (g, h) at 500 mM added NaCl concentration. (i) SAXS scattering curves for 1 mM protamine with different concentrations of NaClO_4_. (j) Kratky representation of the SAXS data. The curves progressively bend downward for *q* > 1.5 nm^–1^ for increasing NaClO_4_ concentrations, indicating a compaction of the protamine chain. (k) The Flory excluded volume parameter ν, obtained from fitting the SAXS curves with a generalized Gaussian chain model, decreases slightly from 0 to 25 mM NaClO_4_ indicating compaction of the chain. The decrease at 400 mM is likely due to complexation of multiple protamines.

Analysis of the ion distribution per residue (Figure 2d, and Supporting Figure 17) reveals that ions bind specifically to arginine-rich regions, where multivalent ‘binding pockets’ are temporally formed between neighboring guanidinium groups (Figure 2e,f), and/or backbone NH and α-protons (Figure 2g). In arginine-poor regions (Figure 2h), binding to the backbone is significantly reduced, showing that ion binding is sequence-specific and is a cooperative effect between the backbone and guanidinium protons.

Analysis by small-angle X-ray scattering (SAXS) shows that the strong binding of chaotropic anions to protamine induces compaction of the peptide. Scattering curves of protamine for high NaClO_4_ concentrations exhibit a sharper decay at high *q* than for low NaClO_4_ concentrations (Figure 2i, and Supporting Information Section 3.3.2) and bend downward from *q* > 1.5 nm^–1^ in the Kratky plot (Figure 2j). By contrast, scattering curves of protamine with NaF do not show any dependence on salt concentration (Supporting Figure 18). The scattering curves were fitted with a generalized Gaussian chain model with Flory excluded volume parameter ν to quantify the degree of compaction (Figure 2k). These fits indeed demonstrate that the protamine behaves like an ideal Gaussian chain (ν = 0.5) at very low NaClO_4_ concentrations, followed by moderate compaction by ClO_4_^–^ binding at intermediate NaClO_4_ concentrations (ν = 0.45), and strong compaction at high NaClO_4_ concentration (ν = 0.37), demarcating the potential onset of homotypic protamine clusters (vide infra). Such compaction was also expected to be accompanied by a reduction in the radius of gyration (Supporting Table 8). However, this could not be adequately probed due to mixed contributions from interparticle interactions affecting the scattering curves at lower *q* values, in the same region that contains the information about the radius of gyration. The Flory excluded volume parameter, on the other hand, is extracted from the data at high *q* values and is therefore expected to be more reliable in this case (see Supporting Figure 19).

### 2.3 Ion Partitioning into Condensates Follows the “Law of Matching Water Affinities”

Selective binding of salt ions to individual condensate components could result in differential partitioning of these ions into the condensates themselves (Figure 3a). Therefore, we investigated the partitioning of a wide range of salt ions into the protamine/ATP condensates. Interestingly, many salt ions have an NMR-active nucleus, allowing us to measure their concentration simultaneously with all other condensate components with a combination of ^1^H, ^7^Li, ^13^C, ^19^F, ^23^Na, ^25^Mg, ^31^P, ^35^Cl, ^39^K, ^81^Br, and ^133^Cs-NMR spectroscopy (Figure 3b, and Supporting Information Section 3.4). Classical approaches to measure the ion content of condensates can be used at slightly smaller sample volumes, but either have the practical disadvantage that separate techniques are required to measure the ions and other condensate components (e.g., ICP-MS in combination with HPLC)^14,39,41,45^ or are less direct, because they cannot separately measure the anion and cation concentration (e.g., thermogravimetric analysis or tie-line analysis).^40,46,48^ The latter can lead to incorrect conclusions in cases in which the anion and cation partition differently. Our NMR-based approach, however, allows us to individually measure all condensate components—the salt anion and cation, protamine, ATP, and buffer—in both the condensate and dilute phase with a single technique for a wide variety of salts. Moreover, NMR-based detection of ion concentrations opens the possibility for future combination with in situ NMR measurements of condensates.^73,74^

**Figure 3:**
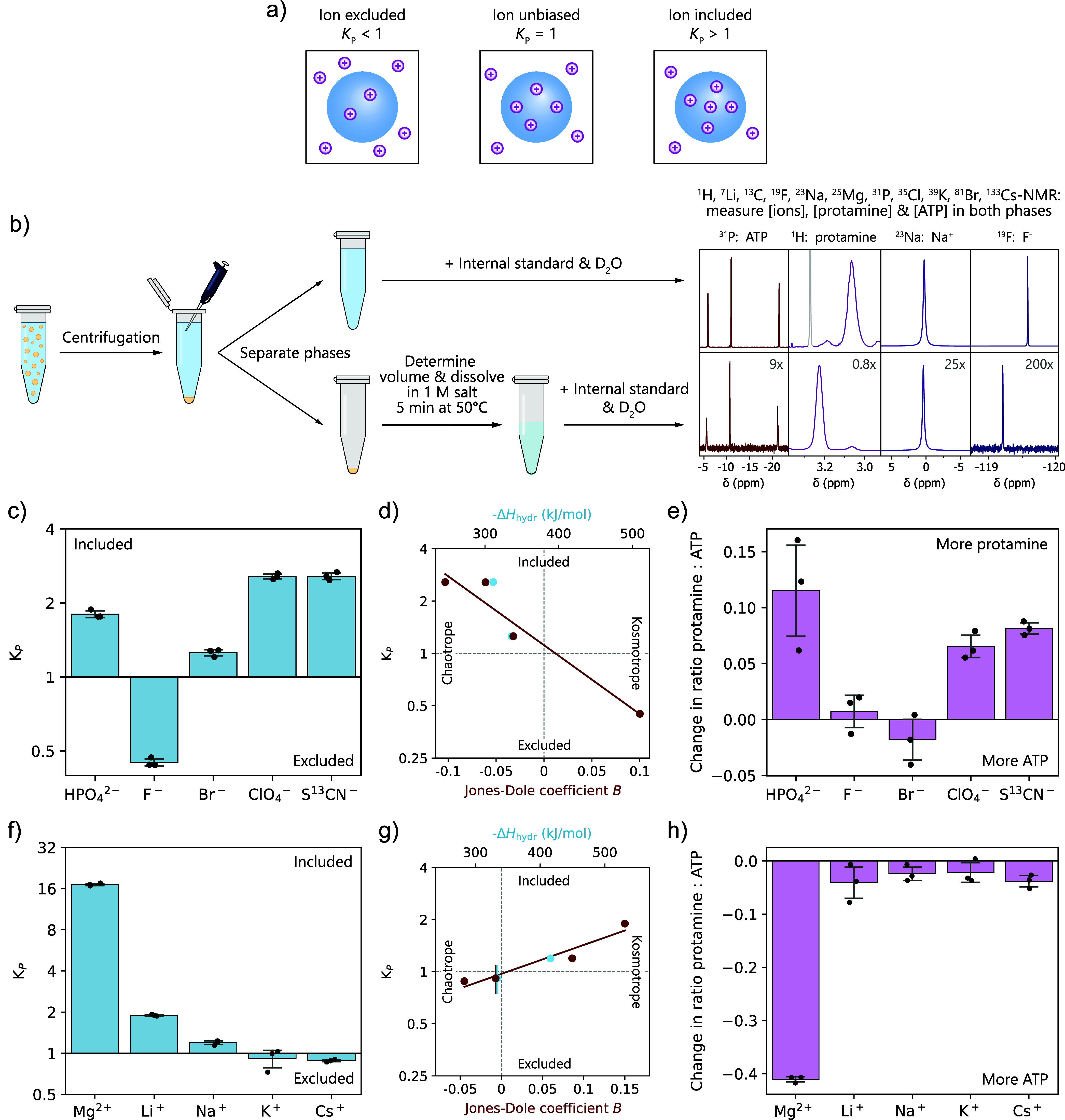
Protamine/ATP condensates selectively partition salt ions that bind to the condensate components. (a) Ions can either be excluded, have no bias, or be included in the condensate phase. (b) Graphical representation of the experimental procedure to measure ion- and condensate component partitioning by ^1^H, ^7^Li, ^13^C, ^19^F, ^23^Na, ^25^Mg, ^31^P, ^35^Cl, ^39^K, ^81^Br, and ^133^Cs-NMR. The NMR spectra are for the dilute (top) and condensate (bottom) phase of protamine/ATP condensates with 100 mM NaF. (c) Partitioning of 100 mM of different anions into protamine/ATP condensates. Chaotropic and divalent anions are included, while kosmotropic anions are excluded. (d) Partitioning of monovalent anions as a function of Jones-Dole coefficient *B* and hydration enthalpy.^70,72^ (e) Change in ratio of protamine:ATP inside the condensate phase upon anion partitioning to maintain the overall charge balance of the condensates. (f) Partitioning of 100 mM of different cations (except for Mg^2+^ which was at 50 mM) into protamine/ATP condensates. Kosmotropic and divalent cations are included, while chaotropic cations are excluded. (g) Partitioning of monovalent cations as a function of Jones–Dole coefficient *B* and hydration enthalpy.^70,72^ (h) Change in ratio of protamine:ATP inside the condensate phase upon cation partitioning to maintain overall charge balance of the condensates.

Using direct NMR spectroscopy of ions and other components in the condensate and dilute phase, we observe distinct partitioning behavior for different ions (Figure 3c,f), which directly follows the binding strengths measured in Section 2.1. For the anions, we observe that the chaotropic (ClO_4_^–^ and SCN^–^) and divalent anions (HPO_4_^2–^), which bind strongly, are included, whereas kosmotropic anions (F^–^) are excluded. For the monovalent anions, the partitioning correlates linearly with their hydration strength (Figure 3d). The transition point between inclusion and exclusion of the investigated ions coincides with a Jones-Dole coefficient *B* = 0, which marks the transition between weakly and strongly hydrated ions (see Supporting Information Section 2.7 for further explanation on the Jones-Dole coefficient),^75^ indicating that weakly hydrated anions in general are included, while strongly hydrated anions in general are excluded. Inclusion of anions in the condensates also changes the condensate composition, reflected by the ratio protamine:ATP (Figure 3e), to retain overall charge neutrality of the condensate (Supporting Figure 53). To confirm that our bulk measurements represent the ion distribution inside individual condensate droplets, we used Raman microscopy to measure the concentration of SCN^–^ inside individual condensate droplets and observe that SCN^–^ indeed localizes to the condensate and is evenly distributed throughout the droplet (Supporting Figure 55).

We observe a similar but reversed trend for the partitioning of cations (Figure 3f). Kosmotropic cations (Li^+^ and Mg^2+^) are included, while chaotropic cations (K^+^ and Cs^+^) are weakly excluded. Again, the transition between inclusion and exclusion coincides with *B* = 0 (Figure 3g), indicating that kosmotropic cations in general are included in condensates, while chaotropic cations in general are excluded. Like anions, cations also change the condensate composition (Figure 3h). In particular, kosmotropic cations compete with protamine for interaction with ATP and decrease the condensate protamine content.

Multivalent ions have stronger interactions with the condensate components, and therefore, they show enhanced partitioning. However, water affinity and the corresponding LMWA still influence their extent of partitioning, as is shown by the different partitioning of HPO_4_^2–^ and Mg^2+^.

The trends we observe in partitioning can also be fitted with lattice energies, which, similar to hydration enthalpy, correlate with ion hardness and have been used in recent literature to explain ion partitioning.^14^ However, they cannot explain the opposite trend in partitioning between anions and cations. While lattice energies correctly reflect the strong interaction between kosmotrope-kosmotrope pairs and therefore can explain the uptake of kosmotropic cations, the LMWA is required to explain the water-release-based favorable interaction between chaotrope-chaotrope pairs and thus the preferential uptake of chaotropic anions into the condensates.

### 2.4 Selective Ion Binding Remodels Condensate Phase Diagrams

It is well known that salt can modulate condensate stability by screening charges and lowering the entropic driving force for condensate formation.^22,49,76^ The critical salt concentration (CSC, typically NaCl) is therefore a conventional estimate of heterotypic condensate stability. However, the differential partitioning we found suggests that, unlike existing theories predict,^55,56^ different ions can influence the phase diagram and stability of condensates in very different ways.

We investigated the effect of selective ion binding on the stability and shape of the phase diagram of the protamine/ATP condensates. To this end, we used our NMR spectroscopy method to measure 5- or 6-dimensional phase diagrams containing the concentrations of protamine, ATP, Tris, and two or three salt ions, allowing us to assess changes in the total condensate and dilute phase composition as a function of salt concentration.

The obtained phase diagrams for LiCl, NaF, NaCl, and NaClO_4_ (Figure 4a–f, and Supporting Information Section 3.5.1) show marked differences. The ion concentrations in the condensate and dilute phase (Figure 4a–c) confirm our findings from Section 2.3 that F^–^ is excluded from the condensates, while Li^+^, Cl^–^, and ClO_4_^–^ are included for all salt concentrations. For Cl^–^ and ClO_4_^–^, which both bind to protamine, the inclusion becomes stronger for higher salt concentrations, and this effect is stronger for the more strongly binding ClO_4_^–^.

**Figure 4:**
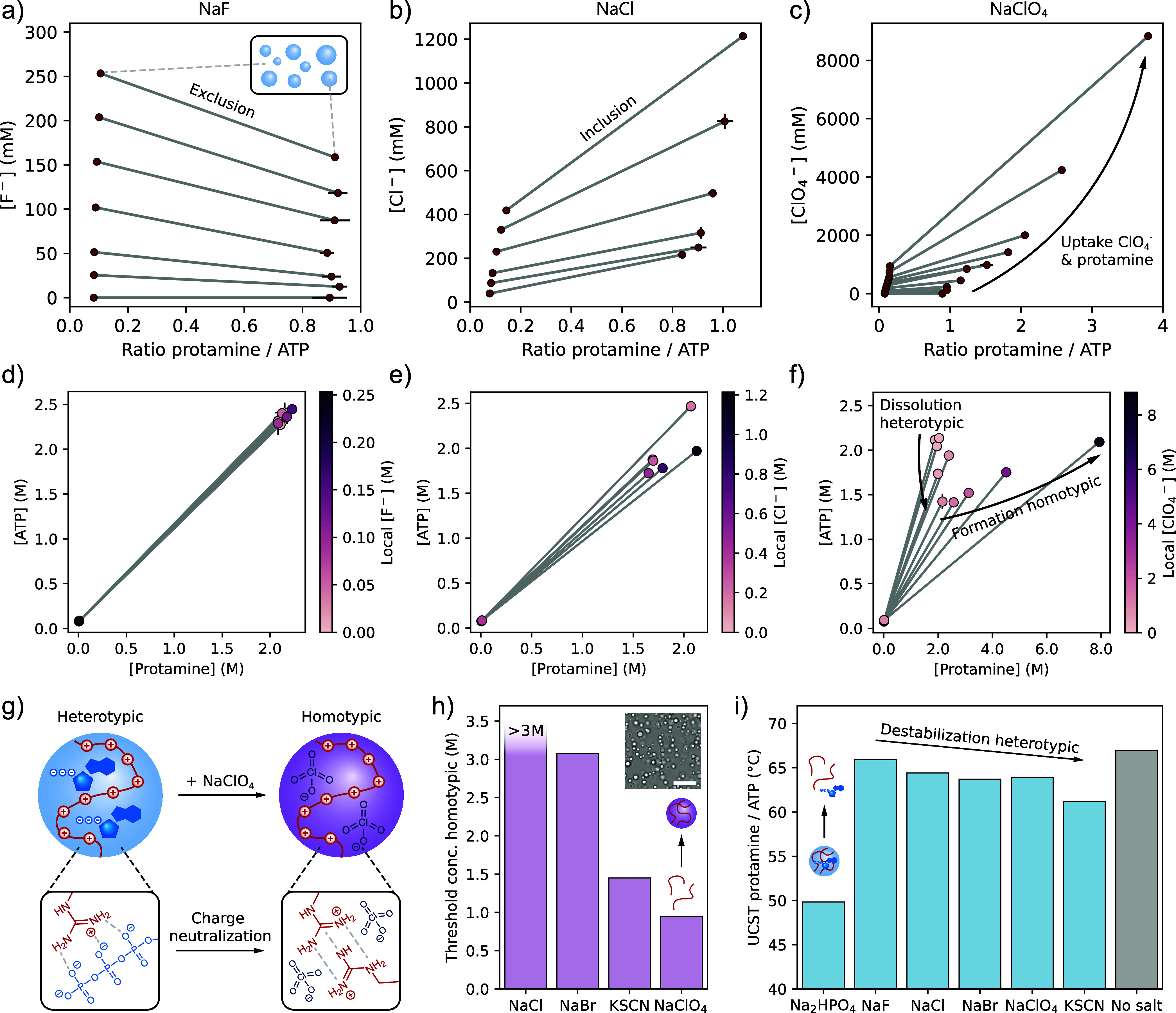
Selective ion binding remodels condensate phase diagrams. (a–c) Phase diagrams of protamine/ATP with increasing concentrations of (a) NaF, (b) NaCl, or (c) NaClO_4_. Concentrations represent the charge concentrations of each species. Gray lines are drawn as a guide to the eye to connect condensate and dilute phase concentrations. (d–f) [ATP] vs [protamine] representation of the same phase diagrams, showing the dissolution of heterotypic protamine/ATP condensates and subsequent formation of homotypic protamine/ClO_4_^–^ condensates for increasing concentrations of NaClO_4_. Gray lines represent tie lines. (g) Schematic illustration of the transition from heterotypic protamine/ATP condensates to homotypic protamine condensates due to charge neutralization by ClO_4_^–^. (h) Chaotropic anions lower the threshold concentration for the formation of homotypic protamine condensates. Inset: Bright-field microscopy image of protamine/ClO_4_^–^ homotypic condensates. Scale bar is 10 μm. (i) Upper critical solution temperature (UCST) for protamine/ATP condensates in the presence of 100 mM of different salts. Chaotropic anions screen protamine’s charges and thereby destabilize heterotypic condensates.

For LiCl, NaF, and NaCl, the condensates dissolve around 500 mM salt, and we observe no change in the protamine:ATP ratio inside the condensates as a function of salt. For NaClO_4_, however, we instead observe a transition from heterotypic condensates that are rich in protamine and ATP, to homotypic protamine condensates that are neutralized by ClO_4_^–^ (Figure 4g). Arginine-rich peptides are known to form homotypic condensates at high salt concentrations, mediated by π-π stacking interactions between the arginine moieties.^24^ Due to the strong binding of ClO_4_^–^ to protamine, the multivalent ATP is gradually replaced by the monovalent ClO_4_^–^ (Figure 4c,f), while ClO_4_^–^-induced compaction (Figure 2i–k) increases the local protamine concentration. As a result, homotypic condensates are formed that now localize Cl^–^, Na^+^, and Tris more strongly than before (Supporting Figure 58). Using a plate reader turbidity assay, we determined the threshold salt concentration required to form homotypic protamine-anion condensates in the absence of ATP and observed that the ability of anions to promote homotypic condensate formation is highly dependent on their chaotropicity (Figure 4h), as strongly binding anions can more effectively neutralize the positive charges on protamine.

The effect of ions on condensates is, however, more nuanced than this. Anions that bind strongly to protamine may promote homotypic phase separation, but they destabilize charge-balanced heterotypic protamine/ATP condensates. In the presence of 100 mM of anions, the upper critical solution temperature (UCST) is significantly reduced for strongly binding and divalent ions HPO_4_^2–^, ClO_4_^–^, and SCN^–^ (Figure 4i), while it is hardly affected by F^–^.

Interestingly, specific ion binding can also favor phase separation at a charge imbalance. When ATP is titrated into a solution of protamine and ions, there is a large excess of positively charged protamine. Anions that strongly bind to protamine lower the excess of positive charge and thereby favor phase separation with ATP and lower the critical ATP concentration required for condensate formation ([ATP]_crit_) (Supporting Figure 63). Cations that bind to ATP, however, increase the charge imbalance by neutralizing negative charge on ATP and thereby increase the [ATP]_crit_. Ions that do not bind to the condensate components generally also increase the [ATP]_crit_ due to nonspecific charge-screening effects.

These results show that specific ion binding modulates condensate phase diagrams in a multitude of ways. We observe similar trends for other heterotypic condensate systems (protamine/D_30_, K_30_/D_30_, and protamine/polyA; Supporting Information Section 3.5.3), and for a homotypic hydrophobic condensate formed by the short peptide derivative LLssLL^77^ (Supporting Information Section 3.5.4). Interestingly, for these homotypic condensates, we observe that both specific ion binding and salting-out effects play a role, as strongly chaotropic anions reduce the LCST through binding and neutralizing the two N-termini of the peptide derivative, while strongly kosmotropic anions reduce the LCST via general salting-out effects.

### 2.5 Ion Binding Changes Viscosity and Interface Potential of Condensates

Crucially, selectively binding ions can also affect the physical properties and function of condensates. We measured the viscosity and interface potential (ζ-potential) of protamine/ATP condensates in the presence of different salts. Using raster image correlation spectroscopy (RICS), we observe that most salts lower the viscosity of the protamine/ATP condensates (Figure 5a), as expected.^78^ However, salts containing strongly binding ions, such as ClO_4_^–^ and Mg^2+^, increase the viscosity by neutralizing charges on the protamine and ATP, facilitating π–π and hydrogen-bonding interactions between protamines or ATPs, respectively, and compacting protamine. Interestingly, we observed previously (Figure 4i, and Supporting Figure 54) that these ions destabilize the protamine/ATP condensates at charge balance, showing that there is no direct linear relation between condensate stability and viscosity.

**Figure 5:**
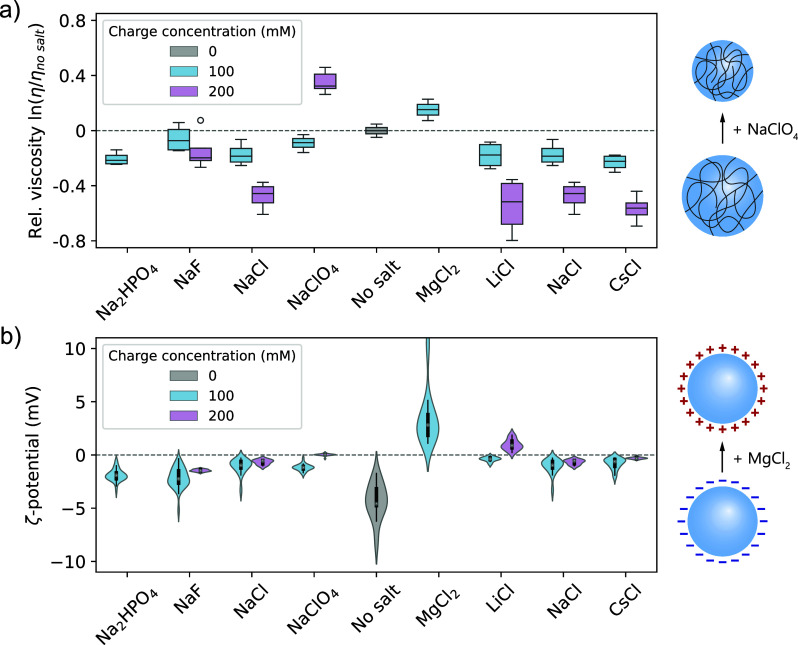
Ion binding and partitioning change the condensate viscosity and interface potential. (a) Ions that bind strongly to the condensate components (ClO_4_^–^ and Mg^2+^) increase the condensate viscosity, even though they disfavor phase separation at charge balance. Outliers are represented as empty circles. Experiments were performed with 100 and 200 mM of monovalent salts and 50 mM of salts containing divalent ions (=100 mM charge concentration). (b) Strongly binding cations (Mg^2+^ and Li^+^) can flip the interface (ζ-)­potential of protamine/ATP condensates from negative to positive.

Using microelectrophoresis,^79^ we measured the ζ-potential of condensates with different salts. Protamine/ATP condensates naturally have a negative ζ-potential (−4.3 mV), and for most salts, the ζ-potential remains negative (Figure 5b). However, addition of either 50 mM MgCl_2_ (100 mM charge concentration) or 200 mM LiCl flips the ζ-potential to positive (+3.5 or +1.0 mV, respectively), showing that binding of specific ions can also invert the interface potential. Importantly, this charge reversal contradicts the observed decrease in the protamine:ATP ratio inside the condensates (Figure 3h), indicating that the interface potential is not always determined by the macromolecular components, but instead small ionic components may localize at the interface and dictate the interface potential.

### 2.6 Ion Binding Alters RNA Duplex Stability in Condensates

Considering that the local environment inside condensates can have profound effects on biochemical processes inside,^3,4,77,80^ we investigated the stability of RNA and DNA duplexes inside the protamine/ATP condensates. We used complementary decamer strands labeled with a Cy3/Cy5 Förster resonance energy transfer (FRET) pair and analyzed the FRET intensity inside the condensates and in buffer using confocal fluorescence microscopy (Figure 6a) for condensates with 100 mM of different monovalent salts or 50 mM of MgCl_2_.

**Figure 6:**
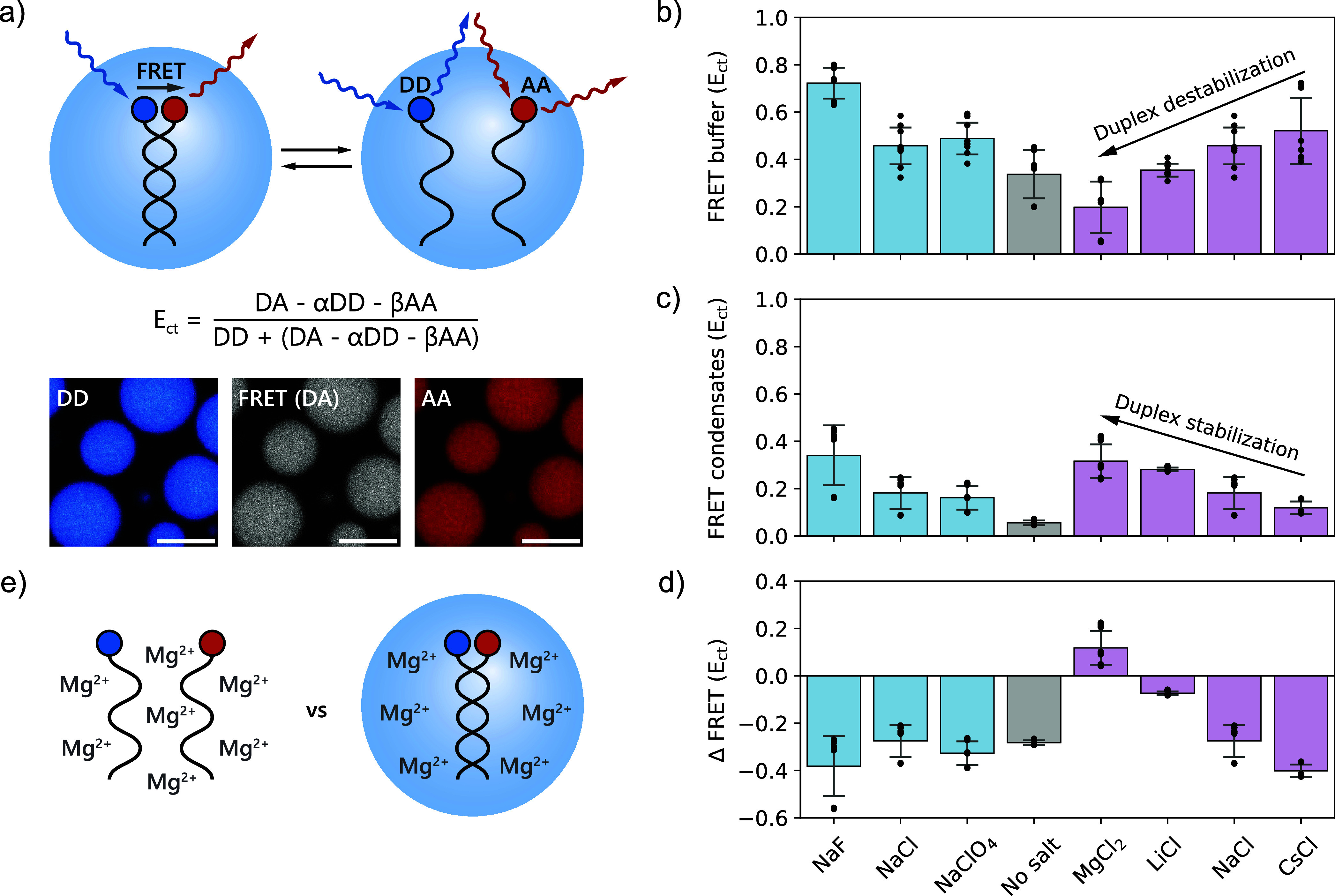
Ion binding and partitioning affect RNA duplex formation inside condensates. (a) Schematic illustration of FRET analysis of the duplex stability of a Cy3/Cy5-FRET pair labeled set of complementary decamer RNAs. Fluorescence microscopy images of the Cy3 donor–donor excitation and emission (DD), Cy3 donor excitation and Cy5 acceptor emission (FRET, DA) and Cy5 acceptor-acceptor excitation and emission (AA) in protamine/ATP condensates with 100 mM NaCl. Scale bar is 10 μm. (b) Corrected FRET efficiency of RNA in buffer. (c) Corrected FRET efficiency inside protamine/ATP condensates with 100 mM of monovalent salts or 50 mM of MgCl_2_. (d) Change in FRET efficiency between the condensate and dilute phase (ΔFRET = FRET efficiency condensate – FRET efficiency buffer). (e) Schematic illustration of the stabilization of RNA duplexes in condensates in the presence of Mg^2+^.

In general, the condensate environment destabilizes RNA duplexes (Figure 6b–d), in agreement with previous reports.^81,82^ However, the addition of salt reduces this destabilization. For kosmotropic anions, such as F^–^, duplex stability is enhanced both in buffer and inside condensates, likely because it lowers the solubility of the free nucleobases in the single-stranded state. For cations, however, we observe a complete inversion of their effects on duplex stability. While the kosmotropic cations Mg^2+^ and Li^+^ destabilize the duplex in buffer, they favor duplex formation in condensates (Figure 6e). According to the LMWA, individual charge–charge interactions between RNA and protamine are relatively weak, and therefore, the stronger-binding Mg^2+^ and Li^+^ may weaken the interaction with protamine and favor duplex formation. We also observe a significant increase in RNA partitioning in the presence of MgCl_2_, which may be due to the positive ζ-potential of the droplets (Supporting Figure 67). We observe similar trends for DNA (Supporting Figures 68–69), showing that ion binding can fundamentally alter condensate properties, and possibly their function as passive helicases.

## 3 Discussion

We have shown that protamine/ATP condensates selectively bind and localize chaotropic anions and kosmotropic cations. Because organic cations in biomolecules are all chaotropic, while biomolecular anions are all kosmotropic, we expect condensates containing charged groups in general to localize chaotropic anions more strongly than kosmotropic anions with the same charge and to localize kosmotropic cations more strongly than chaotropic cations. This trend is in general agreement with other reports on selected ions.^39,40,44,51,83^ However, for some condensates, the transition point between inclusion and exclusion may not lie exactly at *B* = 0. For instance, when condensates are formed at a charge imbalance of the condensate components, this may result in inclusion of all types of cations for an excess of negative charge, and vice versa.^45^ In addition, condensate formation of long polymers is initially favored by addition of low concentrations of salt (Supporting Information Section 3.5.3),^49^ as the salt ions can screen the repulsion between like charges on the polymer chains. In these systems, it may be the case that at low salt concentrations, all ion types are localized to the condensates. Nevertheless, we still expect the general preferences for kosmotropic cations and chaotropic anions to hold and therefore result in stronger partitioning.

The observation that charge–charge interactions are correlated with the hydration strengths of the interacting species via the “law of matching water affinities” prompted us to rethink such interactions in the context of biomolecular condensates. The entropic rearrangement of water molecules during ion complexation plays a key role in charge–charge interactions, and therefore, charge–charge interactions should not be considered as purely enthalpic, as is typically done. Specific ion binding may also help to explain the thermodynamic properties of charge-based condensate formation, which involves counterion release and is typically entropy-driven^18–21,84,85^ and sometimes even enthalpically unfavorable^18,21,85^ (see Extended Discussion for more details; Supporting Information Section 4.1).

Finally, it is worth noting that other approaches have been used in the literature to quantify and explain the interactions of ions with macromolecules, which we discuss in detail in the Extended Discussion (Supporting Information Section 4.2). Specifically, preferential interaction coefficients based on Kirkwood-Buff integrals^86–88^ and the surface-bulk partitioning model based on them^89,90^ have been used as an assumption-free approach to relate macroscopic thermodynamic properties such as solubility to molecule-level interactions between the water, protein, and ions. These methods can give useful insights into the effect of ion addition on the relative interaction preferences of the different molecules in a system, and show that changes in protein chemical potential upon addition of solutes are typically not caused by changes to bulk water structure,^87,91,92^ as classical explanations of the Hofmeister series assume, but rather by water-mediated protein-solute interactions,^93^ which we investigated in this work. Downsides of Kirkwood-Buff-based approaches are that information on specific ion-interaction sites on proteins can only be obtained through simulations^88^ or extensive measurements on small model compounds^90^ and the theory does not provide an explanation for these interactions. The site-specific NMR binding analysis in combination with the LMWA we employed here does provide a direct measurement of site-specific interactions and an explanation for these binding events that can be complementary to the insights obtained by Kirkwood-Buff theory.

## 4 Conclusions

Through thorough NMR analysis, we obtained detailed molecular insight into ion binding and uptake in biomolecular condensates. Interactions between condensate components and salt ions follow the “law of matching water affinities” (LMWA), resulting in strong binding between chaotropic anions and cationic proteins and between kosmotropic cations and nucleic acids or anionic proteins. This results in the strongest inclusion of kosmotropic cations (Li^+^, Mg^2+^, Ca^2+^, and to a lesser degree Na^+^), and chaotropic anions (ClO_4_^–^, I^–^, and to a lesser degree Br^–^ and Cl^–^), but exclusion of Cs^+^, K^+^, and F^–^. Ion binding shapes the condensate microenvironment by altering the composition, viscosity, and interface potential. Such changes can have profound effects on biochemical processes taking place inside the condensates, as we show for RNA duplex formation.

Our findings also provide further insight into the driving forces behind partitioning of small molecules into condensates, and may explain how condensates can modulate subcellular ion distributions and regulate cellular electrochemistry through interphase potentials.^12,13^ They further show that solvent effects should be explicitly considered when investigating interactions between guest molecules and condensates, for instance, in the context of wastewater treatment^15–17^ and delivery of small-molecule therapeutics.^52–54^

## Supplementary Material



## Notes

Full description of experimental procedures, supporting NMR spectra and figures of ion binding, MD simulations, SAXS measurements, ion partitioning, full phase diagrams, critical temperatures and polyion concentrations, and DNA duplex stability in condensates (PDF)

## Abbreviations

ATP: adenosine 5′-triphosphate
NMR: nuclear magnetic resonance spectroscopy
*K*_D,app_: apparent dissociation constant
Δδ: change in NMR chemical shift
LMWA: law of matching water affinities
MD: molecular dynamics simulations
RDF: radial distribution function
αH: protamine α-proton
SAXS: small-angle X-ray scattering
ν: Flory–Huggins excluded volume parameter
*K*_P_: partition coefficient
ICP-MS: inductively coupled plasma mass spectrometry
UCST: upper critical solution temperature
[ATP]_crit_: critical ATP concentration required for condensate formation
RICS: raster image correlation spectroscopy
FRET: Förster resonance energy transfer
