# Supplementary material for: Selective Ion Binding and Uptake Shape the Microenvironment of Biomolecular Condensates
Source: J Am Chem Soc. 2025 Jul 14;147(29):25692–704. doi: 10.1021/jacs.5c07295 (PMC12291443; doi:10.1021/jacs.5c07295)
Supplement: Supplementary file 1 [file ja5c07295_si_001.pdf]

Supporting Information

Selective ion binding and uptake shape the microenvironment of biomolecular condensates

Iris B. A. Smokers[a], Enrico Lavagna[b], Rafael V. M. Freire[c,d], Matteo Paloni[b], Ilja K. Voets[c,d], Alessandro Barducci[b], Paul B. White[a], Mazdak Khajehpour*[e], Evan Spruijt*[a]

[a] Institute for Molecules and Materials, Radboud University, Heyendaalseweg 135, 6523 AJ Nijmegen, The Netherlands.
[b] Centre de Biologie Structurale, Université de Montpellier, CNRS, INSERM, Montpellier 34090, France.
[c] Laboratory of Self-Organizing Soft Matter, Department of Chemical Engineering and Chemistry, Eindhoven University of Technology, P.O. Box 513, 5600 MB Eindhoven, the Netherlands.
[d] Institute for Complex Molecular Systems, Eindhoven University of Technology, P.O. Box 513, 5600 MB Eindhoven, the Netherlands.
[e] Thomas Young Centre and Department of Chemical Engineering, University College London, London WC1E 7JE, United Kingdom.
[f] Department of Chemistry, University of Manitoba, Winnipeg, Manitoba, R3T 2N2, Canada.
* Correspondence: evan.spruijt@ ru.nl; mazdak.khajehpour@umanitoba.ca

Contents

1. General methods 4
  1.1. Materials 4
  1.2. Nuclear Magnetic Resonance Spectroscopy – Binding assay 4
  1.3. Nuclear Magnetic Resonance Spectroscopy – Partitioning 5
  1.4. Brightfield microscopy 7
  1.5. pH measurements 7
2. Experimental methods 7
  2.1. Design of model system 7
  2.2. Condensate formation 8
  2.3. NMR assay for ion binding to condensate components 8
    2.3.1. Binding to the α-proton 8
    2.3.2. Binding to $^{15}N_2$-guanido-labeled arginine 9
    2.3.3. Binding to ATP 9
    2.3.4. Binding to $(GHGLY)_3$ 10
    2.3.5. Justification of the binding model 10
  2.4. Molecular dynamics simulations 11
  2.5. Small-Angle X-ray Scattering (SAXS) measurements 12
  2.6. NMR calibration curves 13
  2.7. Ion partitioning 13
  2.8. Condensate volume fraction determination by cell counting tubes 15
  2.9. Raman microscopy of SCN^- concentration in individual condensate droplets 15
  2.10. Phase diagrams for NaF, NaCl, $NaClO_4$ and LiCl 16
  2.11. Determination threshold salt concentration homotypic condensates 16
  2.12. Determination of protamine/ATP UCST and LLssLL LCST 16
  2.13. Determination of critical polyion concentration of condensates 17
  2.14. Viscosity determination using Raster Image Correlation Spectroscopy (RICS) 17
  2.15. ζ-potential determination by microelectrophoresis 18
  2.16. FRET microscopy analysis of RNA and DNA duplex stability 18
    2.16.1. Preparation of FRET pair oligo stock solutions 18
    2.16.2. Sample preparation for FRET measurements 19
    2.16.3. FRET measurement by confocal microscopy 19
3. Supporting Data 21
  3.1. General supporting data 21
  3.2. Supporting data 'Selective binding of ions to condensate components' 21
    3.2.1. Binding to the α-proton 21

3.2.2. Binding to $^{15}N_2$-guanido-labeled arginine 22
3.2.3. Binding to ATP 24
3.2.4. Binding to $(GHGLY)_3$ 28
3.3. Supporting data 'Ion binding is sequence specific and compacts protamine' 31
3.3.1. Supporting data MD-simulations 31
3.3.2. Supporting data SAXS measurements 33
3.4. Supporting data 'Ion partitioning into condensates follows LMWA' 34
3.4.1. NMR calibration curves 34
3.4.2. NMR spectra and measured concentrations ion partitioning 44
3.4.3. Supporting data ion partitioning 56
3.4.4. SCN^- partitioning by Raman microscopy 57
3.5. Supporting data 'Selective ion binding remodels condensate phase diagrams' 58
3.5.1. Phase diagrams 58
3.5.2. $[ATP]_{crit}$ protamine/ATP 67
3.5.3. $[Polyion]_{crit}$ of other heterotypic condensates 67
3.5.4. Salt effects on a homotypic hydrophobic condensate 69
3.6. Supporting data 'Ion binding changes viscosity and interface potential of condensates' 70
3.7. Supporting data 'Ion binding alters RNA duplex stability in condensates' 71
4. Extended Discussion 73
4.1. Rethinking charge-charge interactions 73
4.2. Comparison to other approaches to investigate protein-ion binding 74
5. Data repository 75
6. References Supporting Information 76

1. General methods

1.1. Materials

All chemicals and reagents were used as received from commercial suppliers. We used MilliQ (MQ) water (i.e., ultrapure deionized water) from Millipore Corporation.

Protamine chloride grade V, adenosine 5'-triphosphate disodium salt hydrate, poly(A) potassium salt, potassium thiocyanate, potassium thiocyanate-^{13}C (99% ^{13}C), sodium iodide, sodium bromide, sodium chloride, sodium fluoride, sodium formate, sodium acetate, sodium phosphate dibasic anhydrous, calcium chloride dihydrate, magnesium chloride hexahydrate, spermidine trihydrochloride, lithium chloride anhydrous, potassium chloride, cesium chloride, tetramethylammonium chloride, deuterium oxide, hexamethylphosphoramide, phosphonoacetic acid, ethylenediaminetetraacetic acid disodium salt dihydrate and ^{15}N-ammonium chloride were purchased from Sigma-Aldrich / Merck.

Tris-(hydroxymethyl)-aminomethane (Tris), volumetric 1.0 M hydrochloric acid, volumetric 1.0 M sodium hydroxide, sodium perchlorate, sodium phosphate monobasic anhydrous, sodium sulfate, L(+)-Tartaric acid disodium salt dihydrate and sodium trifluoromethanesulfonate were purchased from Fisher Scientific.

L-Arginine·HCl (guanido-$^{15}N_2$, 98%) and sodium acetate-1,2-$^{13}C_2$ were purchased from Cambridge Isotope Laboratories, Inc. Sodium nitrate and guanidine hydrochloride were purchased from VWR International B.V. $(GHGLY)_3$ peptide lyophilized TFA salt was purchased from CASLO ApS. Putrescine dihydrochloride was purchased from Bio Connect B.V. 3-(Trimethylsilyl)propionic-2,2,3,3-d_4 acid sodium salt was purchased from Deutero GmbH.

Poly-L-lysine hydrobromide (x = 30, MW=6,300 Da) and poly-L-aspartic acid sodium salt (x=30, MW=4,100 Da) were purchased from Alamanda polymers. pLL-*g*-PEG was purchased from SuSoS AG. LLssLL was synthesized according to the procedure in Abbas & Lipiński et al.[1]

Cy3- and Cy5-labelled DNA and RNA oligos for FRET measurements were purchased from Integrated DNA Technologies (IDT).

1.2. Nuclear Magnetic Resonance Spectroscopy – Binding assay

^{1}H, ^{15}N and ^{31}P-Nuclear Magnetic Resonance (NMR) spectra were measured on a Bruker-AVANCE III 500 MHz spectrometer equipped with a Prodigy BB cryoprobe at 278.15 K for ^{1}H and ^{31}P-NMR spectra and at 298.15 K for ^{15}N-NMR spectra, with a ^{1}H frequency of 500.13 MHz, a ^{15}N frequency of 50.68 MHz and a ^{31}P frequency of 202.46 MHz.

NMR samples were prepared in coaxial NMR tubes with the sample in the outer tube and an internal standard in the inner tube: 3-(trimethylsilyl)propionic-2,2,3,3-d_4 acid in D_2O for ^{1}H-NMR for binding to the α-proton and $(GHGLY)_3$, phosphonoacetic acid in 9 : 1 H_2O : D_2O for ^{1}H and ^{31}P-NMR for binding to ATP, and ^{15}N-ammonium chloride in 9 : 1 H_2O : D_2O for ^{15}N-NMR for binding to ^{15}N-labelled arginine. Chemical shifts were determined in reference to the internal standard. All data was processed in MestReNova 14 and Python.

For ^{1}H-NMR, a pulse sequence was used with 8 or 64 transients (ns = 8 or 64), a P1 pulse length of 12.0 µs which corresponds to approximately a 30° pulse angle and a d1 relaxation delay of 4.0 s. For ^{1}H-NMR of ATP binding, a solvent suppression sequence was used for the measurement, as a 9 : 1 H_2O : D_2O solvent was used. For ^{15}N-NMR, a pulse sequence was set up with 128 transients (ns = 128),

a P1 pulse length of 15.0 µs which corresponds to approximately a 90° pulse angle and a d1 relaxation delay of 6 s. For ^{31}P-NMR, a pulse sequence was set up with 64 transients (ns = 64), a P1 pulse length of 13.0 µs which corresponds to approximately a 90° pulse angle and a d1 relaxation delay of 5 s.

1.3. Nuclear Magnetic Resonance Spectroscopy – Partitioning

^{1}H, ^{7}Li, ^{13}C, ^{19}F, ^{23}Na, ^{31}P, ^{35}Cl and ^{81}Br-Nuclear Magnetic Resonance (NMR) spectra were measured on a Bruker-AVANCE III 500 MHz spectrometer equipped with a Prodigy BB cryoprobe at 298.15 K, with a ^{1}H frequency of 500.13 MHz, a ^{7}Li frequency of 194.37 MHz, a ^{13}C frequency of 125.77 MHz, a ^{19}F frequency of 470.53 MHz, a ^{23}Na frequency of 132.29 MHz, a ^{31}P frequency of 202.46 MHz, a ^{35}Cl frequency of 49.0 MHz and a ^{81}Br frequency of 135.07 MHz. ^{25}Mg and ^{39}K-NMR spectra were measured on a Bruker-AVANCE III 400 MHz HD nanobay spectrometer equipped with a BBFO probe at 298.15 K, with a ^{25}Mg frequency of 24.50 MHz and a ^{39}K frequency of 18.68 MHz. ^{133}Cs-NMR spectra were measured on a JEOL JNM-ECZ500R/S3 RoyalHFX 500 MHz spectrometer equipped with an ROHFX probe at 298.15 K, with a ^{133}Cs frequency of 65.51 MHz.

Quantitative NMR measurements were performed for all nuclei. Either in reference to an internal standard or to a calibration curve. Relaxation delay d1 values were determined such that all ions and internal standards are fully quantitative (d1 $\geq$ 5x T1). All data was processed in MestReNova 14, and OriginPro 2020b.

For ^{1}H-NMR, concentrations were determined in reference to a hexamethylphosphoramide (HMPA) internal standard. A solvent suppression sequence was used for the measurement of ^{1}H-NMR spectra, as a 9 : 1 H_2O : D_2O solvent was used. A pulse sequence was used with 64 transients (ns = 64), a pulse length of 12.0 µs which corresponds to approximately a 30° pulse angle and a d1 relaxation delay of 4.0 s.

For ^{7}Li-NMR, a pulse sequence was set up with a P1 pulse length of 12.15 µs which corresponds to approximately a 90° pulse angle and a d1 relaxation delay of 200 s, in order to ensure full relaxation of the nuclei between each transient. For the dense phase samples 16 transients (ns = 16) were taken with a set receiver gain of rg = 2050. For the dilute phase samples 8 transients (ns = 8) were taken with a set receiver gain of rg = 1030. Concentrations were determined in reference to a calibration curve (Supporting Figures 20-21), for which the receiver gain and pulse length were kept constant.

For ^{13}C-NMR, a pulse sequence was set up with a P1 pulse length of 10.5 µs which corresponds to approximately a 90° pulse angle and a d1 relaxation delay of 212 s, in order to ensure full relaxation of the nuclei between each transient. For the dense phase samples 64 transients (ns = 64) were taken. For the dilute phase samples 32 transients (ns = 32) were taken. Concentrations were determined in reference to a sodium acetate-1,2-$^{13}C_2$ internal standard.

For ^{19}F-NMR, a pulse sequence was set up with 128 transients (ns = 128), a P1 pulse length of 15 µs which corresponds to approximately a 30° pulse angle and a d1 relaxation delay of 10 s, in order to ensure full relaxation of the nuclei between each transient. O1P was set to -125 ppm. Concentrations were determined in reference to a sodium trifluoromethanesulfonate internal standard.

For ^{23}Na-NMR, a pulse sequence was set up with 1024 transients (ns = 1024), a P1 pulse length of 20.5 µs which corresponds to approximately a 90° pulse angle and a d1 relaxation delay of 2 s, in order to ensure full relaxation of the nuclei between each transient. The receiver gain was set at rg = 1820. Concentrations were determined in reference to a calibration curve (Supporting Figures 22-23), for which the receiver gain (rg = 1820) and pulse length were kept constant.

For ^{25}Mg-NMR, a pulse sequence was set up with a P1 pulse length of 20.0 µs which corresponds to approximately a 90° pulse angle and a d1 relaxation delay of 0.100 s, in order to ensure full relaxation of the nuclei between each transient. For the dense phase samples 20480 transients (ns = 20480) were taken. For the dilute phase samples 4096 transients (ns = 4096) were taken. For both the receiver gain was set at rg = 203. Concentrations were determined in reference to a calibration curve (Supporting Figures 24-25), for which the receiver gain (rg = 203) and pulse length were kept constant.

For ^{31}P-NMR, a pulse sequence was set up with 64 transients (ns = 64), a P1 pulse length of 13.0 µs which corresponds to approximately a 90° pulse angle and a d1 relaxation delay of 40 s, in order to ensure full relaxation of the nuclei between each transient. Concentrations were determined in reference to a hexamethylphosphoramide (HMPA) internal standard.

For ^{35}Cl-NMR, a pulse sequence was set up with a P1 pulse length of 15.5 µs which corresponds to approximately a 90° pulse angle and a d1 relaxation delay of 2 s, in order to ensure full relaxation of the nuclei between each transient.

For Cl^- the following settings were used: For the dense phase samples either 512 transients (ns = 512) were taken with the receiver gain set to rg = 50.8 or 2048 transients (ns = 2048) were taken with the receiver gain set at rg = 64. For the dilute phase samples 512 transients (ns = 512) were taken with the receiver gain set to rg = 50.8. Concentrations were determined in reference to a calibration curve (Supporting Figures 26-28) for which the receiver gain (rg = 50.8 or 64) and pulse length were kept constant.

For ClO_4^- (and the Cl^- in the $NaClO_4$ samples) the following settings were used: The sweep width was set to 1195 ppm and O1P to 500 ppm. For both the dense and dilute phase samples 1024 transients (ns = 1024) were taken with the receiver gain set to rg = 50.8. Concentrations were determined in reference to a calibration curve (Supporting Figures 29-30 for Cl^-; 31-32 for ClO_4^-), for which the receiver gain (rg = 50.8) and pulse length were kept constant.

For ^{39}K-NMR, a pulse sequence was set up with a P1 pulse length of 25.0 µs which corresponds to approximately a 90° pulse angle and a d1 relaxation delay of 0.100 s, in order to ensure full relaxation of the nuclei between each transient. For the dense phase samples 20480 transients (ns = 20480) were taken with the receiver gain set to rg = 203. For the dilute phase samples 4096 transients (ns = 4096) were taken with the receiver gain set to rg = 101. Concentrations were determined in reference to a calibration curve (Supporting Figures 33-34), for which the receiver gain (rg = 101 or 203) and pulse length were kept constant.

For ^{81}Br-NMR, a pulse sequence was set up with a P1 pulse length of 12.0 µs which corresponds to approximately a 90° pulse angle and a d1 relaxation delay of 1 s, in order to ensure full relaxation of the nuclei between each transient. For the dense phase samples 6144 transients (ns = 6144) were taken with the receiver gain set to rg = 2050. For the dilute phase samples 2048 transients (ns = 2048) were taken with the receiver gain set to rg = 2050. Concentrations were determined in reference to a calibration curve (Supporting Figures 35-36) for which the receiver gain (rg = 2050) and pulse length were kept constant.

For ^{133}Cs-NMR, a pulse sequence was set up with 32 transients (ns = 32), a P1 pulse length of 7.5 µs which corresponds to approximately a 90° pulse angle and a d1 relaxation delay of 23 s, in order to ensure full relaxation of the nuclei between each transient. The receiver gain was set at rg = 50. Concentrations were determined in reference to a calibration curve (Supporting Figures 37-38), for which the receiver gain (rg = 50) and pulse length were kept constant.

1.4. Brightfield microscopy

Samples were transferred to 96- or 384-well plates and imaged on an Olympus IX83 fluorescence microscope equipped with a motorized stage (TANGO Märzhäuser) and LED light source (pE-4000 CoolLED). Images were recorded on a 40× universal plan fluorite objective (WD 0.51 mm, NA 0.75, Olympus) and a temperature controlled CMOS camera (Orca-Flash4.0 Hamamatsu).

1.5. pH measurements

The pH of solutions was determined using a Mettler Toledo Five Easy FE20 pH meter equipped with a Hamilton SpinTrode pH probe, and calibrated with Thermo Scientific Orion standard buffers at pH 4.01. pH 7.00 and pH 10.00.

2. Experimental methods

2.1. Design of model system

The condensate system protamine/ATP was chosen because both components are commercially available and cheap, so we could easily make large quantities of condensate sample for the partitioning experiments. We chose the chloride salt of protamine instead of the more commonly used sulfate salt in order to be able to measure the counterions of both protamine and ATP by NMR spectroscopy. The pH of all solutions was maintained at pH 8.5, in order to have both the arginines in protamine and the phosphates of ATP, as well as all tested ions for the partitioning experiments in a single speciation state. The pH of the buffer and stock solutions was adjusted with minimal amounts of hydrochloric acid or sodium hydroxide, since these ions are already present as counterions of the protamine and ATP, and therefore we would not introduce additional types of ions to the system. To investigate the effect of different ions, we used a series of different anions and cations as sodium- and chloride salts, respectively. We selected anions and cations ranging from strongly kosmotropic (strongly hydrated) to strongly chaotropic (weakly hydrated). In the presence of 100 mM of every tested monovalent type of ion, or 50 mM of divalent ion (to obtain equal charge concentrations of 100 mM for all ions), condensates were observed by Brightfield microscopy as liquid droplets that fused rapidly (Supporting Figure 2).

Throughout this Supporting Information, concentrations are either reported as standard molecule-based (default, as is done in the manuscript unless mentioned otherwise) or as charge-based concentrations. Molecule-based concentrations of salts are defined as the concentration of the ion with highest valency, i.e., 100 mM $MgCl_2$ (molecule-based) is 100 mM of Mg^{2+} ions. Charge-based concentrations are defined as $c_{\text{charge-based}} = |z| c_{\text{molecule-based}}$, where z is the charge of the ion. For $MgCl_2$ that means that 100 mM charge-based represents 50 mM of Mg^{2+} ions, giving 2x 50 mM = 100 mM of positive charge, together with 100 mM of Cl^- ions. For protamine, which has 21 positive charges, 1 mM molecule-based is 21 mM charge-based and for ATP with 4 negative charges, 25 mM molecule-based equals 100 mM charge-based. For monovalent ions, charge-based concentrations equal molecule-based concentrations.

2.2. Condensate formation

To select the most stable protamine/ATP condensate composition, the critical sodium chloride concentration (CSC) of different ratios of protamine : ATP was measured. A composition of 1 mM protamine with 25 mM ATP gave the highest CSC: 585 mM. We assumed that at the maximum CSC, the condensates are charge-neutral.

Condensate emulsions of 1 mM protamine (molecule-based = 21 mM charge-based) and 25 mM ATP (molecule-based = 100 mM charge-based) in 50 mM Tris pH 8.5 were prepared using stock solutions of 4 mM protamine chloride (grade V, Histone free) in 50 mM Tris pH 8.5 and 100 mM adenosine 5'-triphosphate disodium salt hydrate (ATP) in 50 mM Tris pH 8.5. 50 mM Tris pH 8.5 was prepared by dissolving the appropriate amount of Tris-(hydroxymethyl)-aminomethane (Tris) in MQ water and adjusting the pH to 8.5 using 1 M HCl. 4 mM protamine was prepared by dissolving the solid protamine chloride grade V in 50 mM Tris pH 8.5 and adjusting the pH back to 8.5 using 1 M NaOH. The concentration of the protamine stock is molecule-based and based on the typical sequence of protamine H-PRRRRSSSRPIRRRRPRRASRRRRRRGGRRRR-OH (MW = 4235.73 g/mol). 100 mM ATP was prepared by dissolving the appropriate amount of adenosine 5'-triphosphate disodium salt hydrate in 50 mM Tris pH 8.5 and adjusting the pH back to 8.5 using 1 M NaOH. Salt stock solutions were similarly prepared in 50 mM Tris pH 8.5 and the pH was corrected back to 8.5 using 1 M NaOH or 1 M HCl.

For a 1 mL condensate sample, 250 µL 4 mM protamine chloride was added to 500 µL of 50 mM Tris pH 8.5 and the appropriate amount of salt, and the solution was pipetted up and down several times. Subsequently, 250 µL 100 mM ATP was added, upon which the solution became turbid. The emulsion was mixed either by vortexing for a few seconds and inverting the tube 3x or by pipetting up and down several times (for volume determination by cell counting tubes).

2.3. NMR assay for ion binding to condensate components

2.3.1. Binding to the α-proton

NMR samples with 1 mM protamine chloride and 0 – 800 mM salt (molecule-based, see Supporting Information Section 2.1) in D_2O were prepared using stock solutions of 4 mM protamine chloride in D_2O, 2 M NaClO4 in D_2O, 2 M NaI in D_2O, 2 M NaCl in D_2O, 800 mM NaF in D_2O and 800 mM Na_2HPO_4 in D_2O. The pD of the protamine stock solution was corrected to 8.5 using 1 M NaOD. The pD of the salt stock solutions was not adjusted, so no additional salt ions would be introduced. Samples were prepared in D_2O without buffer to minimize the amount of ions in the sample before salt addition. Samples were prepared by mixing 125 µL 4 mM protamine chloride with the appropriate amount of 2 M or 800 mM salt and D_2O was added up to a total volume of 500 µL. The samples were transferred to the outer tube of coaxial NMR tubes. 5 mM (3-(trimethylsilyl)propionic-2,2,3,3-d_4 acid in D_2O was added to the inner coaxial tube as internal standard. The pD was determined for samples with different salt concentrations and never varied by more than 0.77 between 0 and 500 mM salt (Na_2HPO_4: 0.40, NaF: 0.77, NaCl: 0.37, NaI: 0.50, $NaClO_4$: 0.11). ^{1}H-NMR spectra were recorded as described in Supporting Information Section 1.2, and the changes in NMR chemical shift (Δδ) were determined in reference to the internal standard. Apparent dissociation constants $K_{D,app}$ were calculated by fitting the change in chemical shift as a function of salt concentration to Equation 1:[2]

$$\Delta\delta = -Ac_{\text{ion}} + \frac{\Delta\delta_{\text{max}} c_{\text{ion}}}{K_{\text{D,app}} + c_{\text{ion}}} \qquad \text{(Equation 1)}$$

Because the samples already contained Cl^- from the counterions of protamine (23.1 mM), for NaCl binding, this amount was added to the $K_{D,app}$.

2.3.2. Binding to $^{15}N_2$-guanido-labeled arginine

To determine the strength of charge-charge interaction between different anions and the arginines in protamine, we measured binding to ^{15}N-guanidino-labelled arginine monomer. NMR samples with 40 mM $^{15}N_2$-guanido-labeled arginine and 0 – 800 mM salt (molecule-based, see Supporting Information Section 2.1) in 50 mM Tris pH 8.5 in 9 : 1 H_2O : D_2O were prepared using stock solutions of 80 or 160 mM ^{15}N-arginine in 50 mM Tris pH 8.5 and 2 M NaClO4, NaI and NaCl in 50 mM Tris pH 8.5 and 800 mM NaF and Na_2HPO_4 in 50 mM Tris pH 8.5. The pH of the ^{15}N-arginine stock solution was corrected back to 8.5 using 1 M NaOH. The pH of the salt stock solutions was not adjusted, so no additional salt ions would be introduced. Samples were prepared in Tris buffer, to avoid large changes in pH upon addition of high salt concentrations. Samples were prepared by mixing 125 µL or 250 µL 160 or 80 mM ^{15}N-arginine with 50 µL D_2O and the appropriate amount of 2 M or 800 mM salt, and 50 mM Tris pH 8.5 was added up to a total volume of 500 µL. The samples were transferred to the outer tube of coaxial NMR tubes. 100 mM ^{15}N-ammonium chloride in 9 : 1 H_2O : D_2O was added to the inner coaxial tube as internal standard. The pH was determined for samples with different salt concentrations and never varied by more than 0.24 between 0 and 500 mM salt (Na_2HPO_4: 0.14, NaF: 0.23, NaCl: 0.18, NaI: 0.03, $NaClO_4$: 0.24). ^{15}N-NMR spectra were recorded as described in Supporting Information Section 1.2, at 25 ˚C because no peaks were observed for the guanidinium at 5 ˚C due to slow exchange. Changes in NMR chemical shift (Δδ) were determined in reference to the internal standard. Apparent dissociation constants $K_{D,app}$ were calculated by fitting the change in chemical shift as a function of salt concentration to Equation 1. Because the samples already contained Cl^- from the counterions of the ^{15}N-guanidino-labelled arginine and the Tris buffer (61.8 mM), for NaCl binding, this amount was added to the $K_{D,app}$. F^- was deemed non-binding because the error of the fit was larger than the $K_{D,app}$.

2.3.3. Binding to ATP

NMR samples with 25 mM ATP and 0 – 800 mM salt (molecule-based, see Supporting Information Section 2.1) in 50 mM Tris pH 8.5 in 9 : 1 H_2O : D_2O were prepared using stock solutions of 100 mM ATP in 50 mM Tris pH 8.5 and 2 M $MgCl_2$, LiCl, NaCl, KCl, $(CH_3)_4NCl$ and guandinine HCl in 50 mM Tris pH 8.5. The pH of the ATP stock solution was corrected back to 8.5 using 1 M NaOH. The pH of the salt stock solutions was not adjusted, so no additional salt ions would be introduced. Samples were prepared in Tris buffer, because in D_2O the change in pH upon addition of high salt concentrations was larger than 0.8 pH units. Samples were prepared by mixing 125 µL 100 mM ATP with 50 µL D_2O and the appropriate amount of 2 M salt, and 50 mM Tris pH 8.5 was added up to a total volume of 500 µL. The samples were transferred to the outer tube of coaxial NMR tubes. 5 or 20 mM phosphonoacetic acid in 9 : 1 H_2O : D_2O was added to the inner coaxial tube as internal standard. The pH was determined for samples with different salt concentrations and never varied by more than 0.26 between 0 and 500 mM salt ($MgCl_2$: 0.26, LiCl: 0.15, NaCl: 0.16, KCl: 0.01, $(CH_3)_4NCl$: 0.04, guanidinium HCl: 0.23). ^{31}P- and ^{1}H-NMR spectra were recorded as described in Supporting Information Section 1.2, and the changes in NMR chemical shift (Δδ) were determined in reference to the internal standard. Apparent dissociation constants $K_{D,app}$ were calculated by fitting the change in chemical shift as a function of salt concentration to Equation 1. The resulting $K_{D,app}$'s were grouped for the phosphates, the ribose and

the nucleobase. For the ribose, the *g* and *h* H's (Supporting Figure 9) were not taken into account for the average, because of their close proximity to the phosphates. Because the samples already contained Na^+ from the counterions of ATP and from pH adjustment with NaOH (92.9 mM), for NaCl binding, this amount was added to the $K_{D,app}$.

2.3.4. Binding to $(GHGLY)_3$

NMR samples with 1 mM $(GHGLY)_3$ and 0 – 800 mM salt (molecule-based, see Supporting Information Section 2.1) in 50 mM acetate pH 5.0 in 9 : 1 H_2O : D_2O were prepared using stock solutions of 4 mM $(GHGLY)_3$ in 50 mM acetate pH 5.0 and 2 M $NaClO_4$, NaCl, KCl, LiCl and $MgCl_2$ in 50 mM acetate pH 5.0 and 800 mM NaF and in 50 mM acetate pH 5.0. The pH of the $(GHGLY)_3$ stock solution was corrected back to 5.0 using 1 M NaOH. The pH of the salt stock solutions was not adjusted, so no additional salt ions would be introduced. Samples were prepared by mixing 125 µL 4 mM $(GHGLY)_3$ with 50 µL D_2O and the appropriate amount of 2 M or 800 mM salt, and 50 mM acetate pH 5.0 was added up to a total volume of 500 µL. The samples were transferred to the outer tube of coaxial NMR tubes. 5 mM (3-(trimethylsilyl)propionic-2,2,3,3-d_4 acid in D_2O was added to the inner coaxial tube as internal standard. ^{1}H-NMR spectra were recorded as described in Supporting Information Section 1.2, and the changes in NMR chemical shift (Δδ) were determined in reference to the internal standard. Apparent dissociation constants $K_{D,app}$ were calculated by fitting the change in chemical shift as a function of salt concentration to Equation 1. Because the sample already contains 50 mM Na^+ and 14.4 mM Cl^- at 0 mM added salt, these amounts were included in the $K_{D,app}$'s for Na^+ and Cl^-, respectively.

2.3.5. Justification of the binding model

Ionic effects upon NMR spectral shifts are often correlated with Equation 1,[2–5] which has also been adapted to fit changes in LCST.[2,6] This equation assumes that ion addition affects the NMR spectrum via two modalities. First, ion addition changes bulk solvent properties (e.g., water activity, ionic strength, etc.). These have been shown to affect the chemical shift linearly.[2,3]

Second, ions can form contact pairs with the moiety of interest (e.g., the α-proton), in other words, the ion is replacing water molecules hydrating the moiety. If the chemical shift of the contact pair is different from that of the hydrated moiety, the measured NMR spectrum allows the estimation of the fraction of the moiety participating in contact pairs through the relationship:

$$fraction = \frac{\delta_{measured}}{\delta_{max}} \qquad \text{(Equation 1a)}$$

Therefore, if the linear effect of ion addition is accounted for, one can calculate the fraction of α-protons participating in ion contact pairs at any given bulk ion concentration from the NMR spectrum. If this contact pair formation is modeled via a simple binding equation of ion (I) to the peptide (P):

$$fraction = \frac{[PI]}{[PI]+[P]} = \frac{\delta_{measured}}{\delta_{max}} = \frac{[I]}{K_d+[I]} \qquad \text{(Equation 1b)}$$

This gives the non-linear term of Equation 1, where [P] is the concentration of free peptide, [I] the concentration of free ion and [PI] is the concentration of ion-peptide contact pair.

Our observed $K_{D,app}$'s of 150 mM and lower indicate that the interactions of these ions with certain moieties of protamine and ATP are much stronger than that of water molecules. Contact ion formation thus significantly out-competes hydration. We therefore believe it makes sense to describe these effect via a stoichiometric ligand-binding model as in Equation 1.

Equation 1 assumes distinct binding sites on the peptide, where binding at one site does not influence binding at a neighboring site, as is common for a Langmuir-type model. While we believe this is a reasonable assumption for monovalent ions, which create a charge-neutral contact pair upon binding and give a good fit to Equation 1, the binding of multivalent ions can create a local charge inversion upon binding one binding site, which could influence neighboring binding sites. While we can get a decent order-of-magnitude fit for Mg^{2+} binding to ATP (Supporting Figure 10k,l), we do observe that this binding curve has a different shape than the ones for multivalent ions and the weaker binding HPO_4^{2-}, indicating that for a thorough comparison of the binding behavior of multivalent ions, a different model may be more suitable.

It would be formally correct to use the thermodynamic activity instead of concentration in equations that describe ion effects and binding. However, because the linear part of Equation 1 represents a multitude of indirect bulk effects, there is no advantage in using the activity of the ion vs. the ion concentration: the activity coefficients would simply change the value of parameter A. With regards to the non-linear part, it must be emphasized that spectroscopic methods (Equation 1) only yield "apparent dissociation constants" not true dissociation constants, because they only report on the "fraction bound" value. The apparent dissociation constant is defined by $K_{\mathrm{D,app}} = \frac{[P][I]}{[PI]}$. The thermodynamic dissociation constant is defined as $K_T = K_{\mathrm{D,app}} \frac{\gamma_P \gamma_I}{\gamma_{PI}}$, where γ represents the activity coefficient of each species. Using activities of the ion instead of concentrations in Equation 1 has no advantage because this still does not take into account the activities of the "P" and "PI" species. Using Equation 1 with the spectroscopic data yields an average $K_{\mathrm{D,app}}$ over the concentration range of 0 – 500 mM. Most of these $K_{\mathrm{D,app}}$ values obtained are around 150 mM or less. Because non-specific electrostatic effects are the dominant contributors to the magnitude of the activity coefficients at concentrations significantly lower than 1 M, the contribution of the $\frac{\gamma_P \gamma_I}{\gamma_{PI}}$ term is assumed to be similar for all ions. The differences in $K_{\mathrm{D,app}}$ are thus assumed to be indicative of differences in ion affinities towards specific moieties on protamine and ATP.

2.4. Molecular dynamics simulations

Molecular Dynamics (MD) simulations were performed using Gromacs 2022[7] paired with the Des-Amber force-field, which was specifically parametrized for protein-protein interactions and included parameters for the ions.[8] tip4p was used as water model. The nonbonded parameters provided by DES-amber force-field were used for the Cl^- and ClO_4^- ions, while for the F^- we used the parameters provided in Li *et al.*, which are specifically tuned to reproduce the free energy of hydration in combination with tip4p water.[9] Simulations were run with the leap-frog algorithm, with a time-step of 2 fs. Short-range interactions were treated with a cut-off of 1.0 nm. Long-range electrostatic interactions were treated with the PME scheme with a cubic interpolation of order 4. Temperature coupling was set at 298 K with the velocity-rescale algorithm[10] and a time constant 0.1 ps. In production runs, the pressure coupling was set at 1 bar with the Parrinello-Rahman algorithm,[11] with a 2 ps time constant and a compressibility of $4.5 \cdot 10^{-5}$ bar^{-1}. Each production run was preceded by a system minimization: two equilibrations of 100 ps in the nvt and npt ensemble, respectively. The simulation boxes contained 1 protamine and water and different concentrations of ions F^-, Cl^- or ClO_4^-. The total number of ions included the 21 counterions necessary to neutralize the charge of the system, and the ions to produce the following added salt concentrations: 0 mM, 100 mM, 300 mM, 500 mM (Supporting Table 1). The box shape was a rhombic dodecahedron, which forms a hexagonal

lattice with the periodic boundary conditions. The starting volume of simulation boxes was 1450 nm^3 for the Cl^- systems and 707 nm^3 for the ClO_4^- systems, with a minimal distance between the periodic images of 12.7 nm and 10 nm, respectively, as indicated in Supporting Table 1.

Supporting Table 1: Number of excess ion pairs and minimal periodic image distance for each unbiased simulation.

	F^-		Cl^-		ClO_4^-	
	**Excess ion pairs**	**Box size**	**Excess ion pairs**	**Box size**	**Excess ion pairs**	**Box size**
100 mM			89	12.7 nm	43	10 nm
300 mM	128	10 nm	266	12.7 nm	128	10 nm
500 mM			444	12.7 nm	213	10 nm

We initially performed simulations with protamine in an unbiased configuration (for Figure 2a-d) and simulated different salt concentrations. For the Cl^- we ran a set of three replicas of 200 ns for each salt concentration. For ClO_4^-, we let the replicas run for a total of 1.1 µs in order to achieve proper structural convergence. For the F^- ions, we ran a set of three replicas at 300 mM added salt.

Interactions between protamine and ions were measured as hydrogen-ion contacts using the Gromacs mindist tool, counting how many ions were localized within a threshold distance of the protamine hydrogens. The threshold distance was chosen from the radial distribution functions (RDFs), including only atoms within the width of the first visible peak of the interaction. For the Cl^- atoms, these distances were 0.3 nm, 0.3 nm and 0.4 nm for the backbone NH, the α-proton and the guanidine terminal NH groups, respectively. For ClO_4^- ions, the distances were 0.28 nm, 0.36 nm and 0.25 nm, and we counted only the closest oxygen to avoid counting the same ion more than once.

To reproduce the NMR binding curves for Cl^- and ClO_4^- from the binding analysis with MD, binding data up to 500 mM added salt was used and fit to a Langmuir isotherm as in Equation 1, without the linear term:

$$P = \frac{\Delta \cdot c_{\text{ion}}}{K_{\text{D,app}} + c_{\text{ion}}} \qquad \text{(Equation 2)}$$

where P is the average number of protamine hydrogen-ion pairs and Δ is the asymptotic occupancy level.

In the unbiased simulations with Cl^- ions, we consistently observed two distinct conformation populations (Supporting Figure 17a), one in which the protein was folded once onto itself (called closed) and one in which the protein was stretched (called open). To observe exemplary instances of ion binding to specific residues or residue subsets, we extracted one specific configuration from each conformation population and simulated protamine in a fixed open or closed configuration (for Figure 2e-g). To fix the conformation, we used harmonical position restraints on the heavy atoms with a 10^3 kJ/(mol nm^2) force constant. We performed simulations in sets of two replicas of 150 ns of systems with 100 mM and 500 mM added NaCl. Volumetric density maps of ion distributions were measured using the Volmap tool from VMD.

2.5. Small-Angle X-ray Scattering (SAXS) measurements

SAXS measurements were done for samples containing 0 or 1 mM (4.2 mg/mL) protamine and 0, 5, 10, 25, 100, 200 or 400 mM $NaClO_4$ or NaF in 50 mM Tris pH 8.5. Samples were prepared by mixing

buffer with the appropriate amount of 1 M $NaClO_4$ or 800 mM NaF in 50 mM Tris pH 8.5. The appropriate amount of 4 mM protamine in 50 mM Tris pH 8.5 was added just before the measurement, after which the samples were vortexed briefly.

SAXS measurements were performed at the BM29 beamline of the European Synchrotron Radiation Facility (ESRF, Grenoble, France), equipped with a Pilatus3 X 2M 2D detector. The sample-to-detector distance was set to 2.87 meters and the beam wavelength to 1.0 Å. To avoid dust or solid contaminants, samples were centrifuged at 10000 rcf for 10 minutes at 4 °C prior to measurements and the supernatant was used. Samples were measured in 10 exposures of 1 second each at room temperature (22 °C), in constant flow via a flow-through quartz capillary set-up to minimize radiation damage. Backgrounds with the same salt concentration as the sample were measured before each sample. The intensity of the integrated background-subtracted data was plotted as a function of the magnitude of the scattering vector $q = 4\pi\ \lambda^{-1}\ \sin(\theta/2)$, where λ is the wavelength of the beam and θ the scattering angle. Acquired raw data is available through ESRF (DOI: 10.15151/ESRF-ES-1893861834).

Data was fitted in SASFit software[12] by a generalized gaussian coil form factor,[13] including a hard sphere structure factor to account for repulsion between peptides. This model has been used previously in literature to fit SAXS data of peptides.[14,15] The generalized gaussian coil form factor *P(q)* is described in Equation 3-4.

$$P(q) = I_0\left(\frac{1}{\nu U^{\frac{1}{2\nu}}}\gamma\left(\frac{1}{2\nu}, U\right) - \frac{1}{\nu U^{\frac{1}{\nu}}}\gamma\left(\frac{1}{\nu}, U\right)\right) \qquad \text{(Equation 3)}$$

$$U = (2\nu + 1)(2\nu + 2)\frac{q^2 R_G^2}{6} \qquad \text{(Equation 4)}$$

Where I_0 is the forward scattering, γ is the lower incomplete Gamma function, R_G is the radius of gyration, and *ν* is the Flory excluded volume parameter. Theoretical values for ν are: ν = ⅓ for collapsed globules (chains in poor solvents), ν = ½ for ideal (Gaussian) chains (in θ-solvents), and ν = ⅗ for swollen chains (in good solvents).

2.6. NMR calibration curves

NMR calibration curves were measured for samples of 0.5, 1, 2, 3, 5, 10, 17.5, 25, 37.5, 50, 100, 150, 200, 250, 300 and 350 mM salt (molecule-based, see Supporting Information Section 2.1), either in 9 : 1 H_2O : D_2O or in 50 mM Tris pH 8.5 containing 10 % D_2O. Receiver gain settings were selected such that there was no saturation of the signal and a linear trend was obtained. The number of scans was selected such that there was signal for all concentrations in the range. Separate calibration curves were prepared for 0 – 350 mM and 0.5 – 5 mM, which were used for the dilute phase and condensate phase samples, respectively. 0.5 – 5 mM samples were typically prepared in duplo or triplo and measurement triplicates were taken.

2.7. Ion partitioning

To determine the protamine, ATP and ion concentration in the dense and dilute phase for different salts, 10 mL samples of 1 mM protamine / 25 mM ATP condensates were prepared with 100 mM of different salts (molecule-based, see Supporting Information Section 2.1; for $MgCl_2$ 50 mM molecule-based was used), following the procedure in Supporting Information Section 2.2 using 0.5 M salt stock solutions. The samples were left to equilibrate at room temperature for 20 minutes, after which they were centrifuged for 30 min at 3095 RCF and 20°C. After centrifugation, the supernatant was clear

and the condensate was collected as a slightly opaque liquid at the bottom. Most of the dilute phase was removed by micropipette and saved for NMR measurement, making sure that the pipette tip did not touch the condensate phase. The samples were centrifuged again at 3095 RCF and 20°C for 5 minutes to spin down the remaining dilute phase, which was subsequently removed carefully with a micropipette, resulting in an isolated condensate phase. The condensate phase was dissolved by adding 2 - 5 mL 1 M solution of a salt we did not want to measure: 1 M KBr for the partitioning of Na_2HPO_4, NaF, NaCl, NaClO4, CsCl, LiCl and $MgCl_2$, 1 M KCl for NaBr partitioning, and 1 M NaBr for partitioning of $KS^{13}CN$ and KCl. The condensate phase with added salt was heated to 50 °C for 5 minutes, after which it was shaken vigorously, and was allowed to cool to room temperature to check if the solution remained clear at room temperature. NMR samples of the dilute phase were prepared by mixing 400 µL dilute phase with 50 µL 1 M potassium bromide (or other salt used for dissolving the condensate phase), 50 µL D_2O and 5 µL 1 M hexamethylphosphoramide (HMPA) internal standard. NMR samples of the condensate phase were prepared by mixing 450 µL of the dissolved condensate phase with 50 µL D_2O and 2 µL 1 M HMPA internal standard. Concentrations of protamine and Tris were determined using ^{1}H-NMR spectroscopy, the concentration of ATP with ^{31}P-NMR, and the concentrations of salt ions with ^{7}Li, ^{13}C, ^{19}F, ^{23}Na, ^{25}Mg, ^{31}P, ^{35}Cl, ^{39}K, ^{81}Br and ^{133}Cs-NMR spectroscopy. All NMR samples were prepared in triplo. To calculate the dilution factor of the condensate phase upon dissolution and determine the original concentrations in the condensate phase, we used cell counting tubes to determine the volume fraction of condensate phase in the sample, following the procedure in Smokers & Spruijt (Supporting Information Section 2.8).[16]

For $MgCl_2$, one condensate sample was prepared for which the condensate phase was dissolved in 2 mL 1 M KBr and heated to 50 °C for 5 minutes, which was used to measure the concentration of protamine, ATP, Na^+ and Cl^- in the sample. However, because Mg^{2+} is strongly quadrupolar, at neutral pH no signal is observed due to strong binding to ATP. We therefore prepared a sample for which the condensate phase was dissolved in 2 mL 5 M hydrochloric acid and heated to 50 °C for 5 minutes to hydrolyze the ATP and neutralize the charges on the phosphates. This sample was used to measure the concentration of Mg^{2+} in the condensate phase. To measure the concentration of Mg^{2+} in the dilute phase, NMR samples were prepared by mixing 400 µL dilute phase with 50 µL 5 M hydrochloric acid and 50 µL D_2O.

The partition coefficient of Mg^{2+} was verified by ICP-MS to be K_P = 17.9 (± 1.3), in close agreement with the value found by ^{25}Mg-NMR. ICP-MS samples were prepared in triplo for 100x dilution and 1000x dilution. Samples of the dilute phase and dense phase dissolved in HCl were diluted 100x and 1000x in MQ with 1% nitric acid and analyzed on a Thermo Scientific iCAP RQ ICP-MS with a limit-of-detection of 0.7 ng/L for ^{24}Mg and concentrations were determined in reference to a 103Rh internal standard and calibration curve.

To relate the partitioning behavior of the monovalent ions to their water affinity, we made use of the Jones-Dole viscosity B coefficient:[17]

$$\frac{\eta}{\eta_0} = 1 + A\sqrt{c} + Bc \qquad \text{(Equation 5)}$$

where η is the viscosity of an aqueous salt solution, η_0 the viscosity of water, c the concentration of salt, A an electrostatic term, and B the Jones-Dole coefficient. B is a measure for the strength of ion-water interactions, and is normalized to the strength of water-water interactions.[18,19] This parameter is an easily interpretable measure for the water affinity of ions: when B = 0, the ion-water interaction strength is equal to that of water-water interactions; when B > 0, the ion-water interactions are stronger than water-water, meaning that the ion is strongly hydrated; when B < 0, the ion-water

interactions are weaker than water-water, meaning that the ion is weakly hydrated. Therefore, B = 0 is the transition point between weak hydration (chaotropes) and strong hydration (kosmotropes).

2.8. Condensate volume fraction determination by cell counting tubes

PCV cell counting tubes (capillary graduations only, no cap, Sigma-Adrich) were used directly without surface modification. 1 mL condensate samples were prepared directly in cell counting tubes following the preparation method above, after which the emulsion was mixed by pipetting up and down several times. The samples were centrifuged for 30 min at 3100 RCF and 20°C directly after preparation. The condensate volume was read out from the graduations (Supporting Figure 1), and divided by the total sample volume to obtain the volume fraction. All experiments were carried out in triplo.

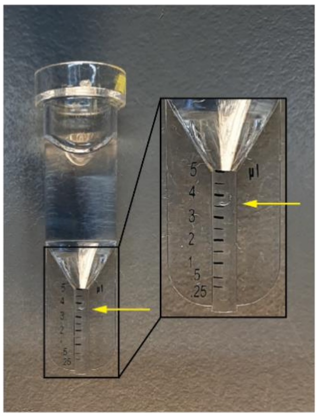

Supporting Figure 1: Read-out of the condensate volume fraction from a cell counting tube. Figure reused from ref[16].

2.9. Raman microscopy of SCN^- concentration in individual condensate droplets

Condensate samples for Raman microscopy were prepared as described in Supporting Information Section 2.2 and added to Ibidi µ-Slide $I^{0.2}$ Luer channel slides, which were modified in advance by cleaning them in a plasma cleaner, modifying for 24 hours using 0.01 mg/mL PLL-*g*-PEG (SuSoS AG) in MQ and subsequently cleaning with MQ and drying with pressurized air. The samples were left to equilibrate and settle on the bottom of the slide for half an hour, after which the slide was inverted and the suspended condensates were visualized using a WITec alpha 300R microscope equipped with a 457 nm laser and Zeiss EC Epiplan-Neofluar 100×/0.9 DIC M27 objective. A 15 mW laser power, 600 g/mm grating (BLZ 500.0 nm) and 10 s integration time were used to obtain per-pixel Raman spectra of a 20×20 µm area (pixel size 1×1 µm). The measurement was performed at the z-height where the protamine and ATP C-H signal ($\tilde{\nu}$ = 2950 cm^{-1}) was maximal, i.e., the slice with maximum signal from the condensate. At the start of each measurement day, the Raman signal was calibrated using a SiO_4 sample ($\tilde{\nu}$ = 520.7 cm^{-1}).

Data was prepared for analysis using the WiTec Project 6.1 software. Cosmic rays were removed using a median filter size (spectral) of 8 and background subtraction was performed using a shape size of 1000. The resulting data was further analyzed using a Python script that fit the signal at $\tilde{\nu}$ = 2064 cm^{-1} (for SCN^-) with a Gaussian curve and plotted the integral of the signal per pixel.

2.10. Phase diagrams for NaF, NaCl, $NaClO_4$ and LiCl

To determine the composition of condensates at different total salt concentrations and make phase diagrams, condensate samples were prepared following the procedure in Supporting Information Section 2.2 with 0 – 300 mM LiCl, 0 – 250 mM NaF, 0 – 400 mM NaCl or 0 – 1 M $NaClO_4$. Phase diagrams were measured using ^{1}H, ^{7}Li, ^{19}F, ^{23}Na, ^{31}P and ^{35}Cl-NMR, for which samples were prepared following the procedure in Supporting Information Section 2.7. Instead of 10 mL total volume, 2 mL was used. The condensate phase was isolated by removing most of the supernatant by micropipette and subsequently taking up the remaining dilute phase by lightly touching it with filter paper. The isolated condensate phase was dissolved in either 1 mL (for volume fractions > 0.1%) or 0.5 mL (for volume fractions < 0.1%) 1 M KBr.

2.11. Determination threshold salt concentration homotypic condensates

The threshold salt concentration for homotypic protamine / salt condensates was determined for different salts using a plate reader turbidity assay. Samples of 1 mM protamine and different concentrations of salts in 50 mM Tris pH 8.5 with a total volume of 100 µL were prepared by mixing 50 mM Tris pH 8.5 and different amounts of salt stock solution and adding 25 µL 4 mM protamine. Stock solutions of 4 mM protamine, 4 M NaCl, 5 M NaBr, 4 M KSCN and 5 M $NaClO_4$ were prepared in 50 mM Tris pH 8.5 and their pH was corrected back to 8.5 using 1 M NaOH or 1 M HCl. Samples were vortexed and transferred to a transparent 96-well plate. The absorbance at 600 nm was measured in a Tecan Spark M10 plate reader and the turbidity was calculated as (100 – T%), with T% being the fraction of transmitted light at 600 nm, calculated as:

$$T_{\%} = \frac{I}{I_0} \cdot 100\ \% = 10^{-Abs} \cdot 100\ \% \qquad \text{(Equation 6)}$$

Where Abs is the measured absorbance at 600 nm. The threshold concentration for homotypic protamine / salt condensate formation was determined by plotting the recorded turbidity (100 – T%) as a function of the salt concentration in the sample, and taking the intersect of the tangent lines to the highest and lowest first-order derivative of the turbidity, calculated by a MATLAB script. For NaCl the threshold concentration could not be measured exactly due to limited solubility of the salt.

2.12. Determination of protamine/ATP UCST and LLssLL LCST

To determine the upper critical solution temperature (UCST) of protamine/ATP condensates in presence of 100 mM of different salts (molecule-based, see Supporting Information Section 2.1), a UV-Vis turbidity assay as a function of temperature was used. Condensate samples of 120 µL were prepared as detailed in Supporting Information Section 2.2 directly in a Hellma Analytics High Precision Cell quartz cuvette with an optical path length of 10x2 mm and center height of 15 mm, suitable for sample volumes of 100 µL. A JASCO V-630 UV-Vis spectrophotometer equipped with Peltier controlled cell holder was used to measure the loss in absorbance at 600 nm as a function of temperature from 20 to 70 ˚C at a ramp rate of 1.0 ˚C per minute. The turbidity was calculated as (100 – T%), with T% being the fraction of transmitted light at 600 nm, calculated using Equation 6. The UCST for protamine/ATP condensates was determined by plotting the recorded turbidity (100 – T%) as a function of the temperature in the sample, and taking the intersect of the tangent lines to the highest and lowest first-order derivative of the turbidity, calculated by a MATLAB script.

A similar procedure was used to determine the LCST of LLssLL condensates. Samples of 3 mg/mL LLssLL (TFA salt) with 100 mM of monovalent salts or 50 mM of divalent $MgCl_2$ or Na_2HPO_4 (molecule-based, see Supporting Information Section 2.1; to obtain equal charge concentrations of all salts) in 50 mM Tris pH 8.5 were prepared by mixing 60 µL 100 mM Tris pH 8.5, 24 µL 0.5 M salt and 36 µL 10 mg/mL LLssLL. The 10 mg/mL LLssLL stock was prepared in MQ water. At room temperature, these samples were clear, but they became turbid upon heating. The appearance of turbidity as a function of temperature was measured using the same method described above, and the LCST was determined using the MATLAB script described above.

2.13. Determination of critical polyion concentration of condensates

The critical polyion concentration of condensates was assessed using a plate reader turbidity assay. 100 µL solution of a polyion with 100 mM of monovalent salt, 50 mM of divalent salt, or 40 mM spermidine (molecule-based, see Supporting Information Section 2.1; to obtain equal charge concentrations of all salts) in 50 mM Tris buffer pH 8.5 were added to 96-well plates (Greiner Bio-one, clear flat bottom) and absorbance at 600 nm was measured on a microplate reader (Tecan Spark M10) equipped with an automated injector. After every readout, 2 µL solution of the oppositely charged polyion was added and the samples were mixed by shaking for 5 s. A total of 40 readouts was made, and all measurements were made in triplo. The following pairs of polyions were tested:

Supporting Table 2: Stock solutions used to determine the critical polyion concentration for condensate formation ($[\text{polyion}]_{crit}$) by plate reader assay.

Polyion in sample	Polyion stock added in titration
0.5 mM protamine	5 mM ATP for monovalent ions, sodium sulphate, sodium tartrate, sodium phosphate; 10 mM ATP for putrescine HCl; 25 mM for calcium chloride, spermidine; 100 mM for magnesium chloride
0.5 mM polyA	75 µM protamine
1 mM protamine	5 mM D_{30}
0.5 mM D_{30}	0.2 mM protamine
4 mM K_{30}	5 mM D_{30}
3 mM D_{30}	10 mM K_{30}

Turbidity was calculated as (100 – T%), with T% being the fraction of transmitted light at 600 nm, calculated using Equation 6. The critical polyion concentration was determined by plotting the recorded turbidity (100 – T%) as a function of the added polyion concentration in the sample, and taking the intersect of the tangent lines to the highest and lowest first-order derivative of the turbidity, calculated by a MATLAB script.

2.14. Viscosity determination using Raster Image Correlation Spectroscopy (RICS)

Condensate samples for RICS were prepared as described in Supporting Information Section 2.2 with 100 or 200 mM salt (charge-based, see Supporting Information Section 2.1; i.e., 100 mM charge-based of $MgCl_2$ or Na_2HPO_4 equals 50 mM molecule-based, while for monovalent salts charge-based concentrations equal molecule-based concentrations) and 0.1 mg/mL 4.4 kDa TAMRA-labelled

dextran added before addition of protamine and ATP. The samples were added to Ibidi 18-well chambered µ-slides with glass bottom, which were modified in advance by cleaning them in a plasma cleaner, modifying for 24 hours using 0.01 mg/mL PLL-*g*-PEG (SuSoS AG) in MQ and subsequently cleaning with MQ and drying with pressurized air.

The viscosity of condensates was determined using Raster Image Correlation Spectroscopy (RICS) on a Leica SP8 confocal microscope equipped with a single-photon detector. Calibration of the focal volume waist ω_0 was performed using the known diffusion coefficient of Alexa 488 of 435 µm^2 s^{-1} (T = 22.5 ± 0.5 °C) in water, and ω_z was set to 3 times the value of ω_0.[20] All measurements were captured at a resolution of 256 × 256 pixels with a 20 nm pixel size using a 63× objective. Condensate samples were measured at 10 Hz line speed with 15 frames acquired per data point. Analysis of autocorrelation curves was done using PAM.[21] Measured diffusion coefficients and respective viscosities for each sample are shown in Supporting Table 29.

2.15. ζ-potential determination by microelectrophoresis

Condensate samples for ζ-potential determination were prepared as described in Supporting Information Section 2.2 with 100 or 200 mM salt (charge-based, see Supporting Information Section 2.1; i.e., 100 mM charge-based of $MgCl_2$ or Na_2HPO_4 equals 50 mM molecule-based, while for monovalent salts charge-based concentrations equal molecule-based concentrations). All samples were imaged on Ibidi 6-well µ-channel slides VI 0.4 with bioinert surface modification. Before image acquisition, 100 µL condensate suspensions were transferred to the channels and incubated for 30 minutes to allow droplets to coalesce and settle on the glass surface. Electrodes (2 mm, silver) connected with copper wires to a BT-305A PSU direct current power source (Basetech) were lowered into opposing ends of the microchannel slide and an electric field of 5 to 12 V/cm was applied, with the cathode at the top of the field of view. Moving condensates were imaged in the middle of the channel of the microslide. Samples were imaged on an Olympus IX83 inverted fluorescence microscope equipped with a motorized stage (TANGO, Märzhäuser) and LED light source (pE-4000 CoolLED). Images were recorded with a 40× universal plan fluorite objective (WD 0.51 mm, NA 0.75, Olympus) with a temperature-controlled CMOS camera (Hamamatsu Orca-Flash 4.0).

Raw microscopy videos were processed and analyzed with MATLAB 2021 Image processing Toolbox and droplet trajectories were determined using the method described in Van Haren et al.[22] ζ-potentials for all detected droplets in a sample were determined from their velocities with a modified Smoluchowski equation, using the applied electric field strength, Debye length calculated from salt concentration and the droplet viscosity determined by raster image correlation spectroscopy (RICS). All parameters used to calculate the condensate ζ-potential are available in Supporting Table 30.

2.16. FRET microscopy analysis of RNA and DNA duplex stability

2.16.1. Preparation of FRET pair oligo stock solutions

For FRET measurements on duplex stability, DNA and RNA decamer complementary strands were used which were labeled with Cy3 on the forward strand and Cy5 on the reverse strand (Supporting Table 3). DNA and RNA oligo stock solutions were prepared by dissolving the oligos in TE buffer (10 mM Tris pH 8.0, 1 mM EDTA) up to final concentrations of 0.1 mM for the Cy3- and Cy5-labeled oligos and of 1 mM for the unlabeled oligos. Upon addition of TE buffer, the samples were vortexed, left for 15

minutes and vortexed again, after which they were aliquoted and stored at -20 ˚C (DNA) or -80 ˚C (RNA) until used. A fresh stock was used for every day of experiments.

Supporting Table 3: DNA and RNA sequences used for FRET analysis of duplex stability.

**Name**	**Sequence (5' – 3')**
Cy3-DNA (forward)	ACCTTGTTCC-Cy3
Cy5-DNA (reverse)	Cy5-GGAACAAGGT
Cy3-RNA (forward)	ACCUUGUUCC-Cy3
Cy5-RNA (reverse)	Cy5-GGAACAAGGU
DNA forward	ACCTTGTTCC
DNA reverse	GGAACAAGGT
RNA forward	ACCUUGUUCC
RNA reverse	GGAACAAGGU

2.16.2. Sample preparation for FRET measurements

For FRET measurements, condensate samples were prepared containing 0.1 µM of both the Cy3- and Cy5-oligo, 50 mM Tris pH 8.5, 15 mM NaCl, 0.5 mM $MgCl_2$, 1 mM protamine, 25 mM ATP and 100 mM of added salt of interest (molecule-based, see Supporting Information Section 2.1; for $MgCl_2$ 50 mM molecule-based was used to obtain equal charge concentrations for all salts). All stock solutions except for the oligo's were prepared in 50 mM Tris pH 8.5. Samples were prepared by mixing Tris buffer, NaCl, $MgCl_2$, the salt of interest and the oligos, after which they were vortexed and centrifuged briefly, heated to 90˚ C for two minutes and left to cool down to room temperature for at least half an hour. Protamine and ATP were added and the samples were vortexed briefly before being transferred to the microscopy slide. For each salt of interest, FRET samples containing both Cy3- and Cy5-labelled DNA or RNA were measured in triplo, and two control samples were measured to determine the FRET correction parameters α and β: one with the Cy3-labelled forward strand and non-labelled reverse strand (donor only), and one with the Cy5-labelled reverse strand and non-labelled forward strand (acceptor only).

Control samples without condensates were prepared in a similar way, but the protamine and ATP were omitted.

2.16.3. FRET measurement by confocal microscopy

Condensate samples were imaged in Ibidi 18-well chambered µ-slides with glass bottom, which were modified in advance by cleaning them in a plasma cleaner, modifying for 24 hours using 0.01 mg/mL PLL-*g*-PEG (SuSoS AG) in MQ and subsequently cleaning with MQ and drying with pressurized air. Before image acquisition, 50 µL condensate suspension was transferred to the well and incubated for at least 5 minutes to allow droplets to settle on the surface. Samples without condensates were imaged in Ibidi 6-well µ-channel slides VI 0.4 (No. 1.5 polymer coverslip) without surface modification. In all cases, triplicate samples and controls were added to separate coverslips to avoid heating during measurement of a neighboring sample.

FRET efficiencies of dsRNA or dsDNA with a Cy3 / Cy5 FRET pair were determined inside condensates using a Leica Sp8x laser scanning confocal microscope equipped with a white-light laser and a 100× magnification oil-immersion objective and the whole setup was incubated at 29 – 30 ˚C. Images were recorded at a line scanning speed of 50 Hz, zoom factor 4.0, a pinhole of 3.0, without averaging or accumulation and saved in 16-bit 256×256 pixels format.

FRET efficiencies in absence of condensates were determined in a similar way, but using a 10× magnification objective, higher laser power, a line scanning speed of 10 Hz, zoom factor 1.00, a pinhole of 3.0, without averaging or accumulation and images were saved in 16-bit 16×16 pixels format. To find the right z-position for the samples, the edge of the channel was put in focus, and this height was used to measure the FRET efficiency inside the channel.

The Cy3 donor was excited at 514 nm and its emission was collected between 555 – 620 nm. FRET emission from the Cy5 acceptor was collected between 650 – 750 nm. The Cy5 acceptor was excited at 633 nm and its emission collected between 650 – 750 nm. Three fluorescence channels were recorded for each image:

DD_{obs}= observed Cy3 donor emission after Cy3 donor excitation

DA_{obs}= observed Cy5 acceptor emission after Cy3 donor excitation (FRET)

AA_{obs}= observed Cy5 acceptor emission after Cy5 acceptor excitation

To correct for the overlap between the emission and excitation wavelengths of the donor and acceptor, the DD, DA and AA measurements described above were also performed for samples containing only donor or only acceptor, with a non-labeled complementary nucleic acid strand and equivalent hardware settings as described above, from which DD_{donor}, DA_{donor}, $DA_{acceptor}$ and $AA_{acceptor}$ were obtained.

ImageJ was used to quantify the fluorescence intensities from the obtained images. Circular regions of interest (ROIs) were selected inside three different droplets per image, and for each ROI the average DD, DA and AA signal was quantified. The corrected FRET intensity was then calculated according to Equation 7:

$$\mathrm{E}_{CT} = \frac{\mathrm{DA}}{\mathrm{DA+DD}} = \frac{\mathrm{DA_{obs}} - \alpha \cdot \mathrm{DD_{obs}} - \beta \cdot \mathrm{AA_{obs}}}{\mathrm{DD_{obs}} + (\mathrm{DA_{obs}} - \alpha \cdot \mathrm{DD_{obs}} - \beta \cdot \mathrm{AA_{obs}})} \quad \text{(Equation 7)}$$

With the correction terms calculated according to Equation 8 and 9:

$$\alpha = \frac{\mathrm{DA_{donor}}}{\mathrm{DD_{donor}}} \quad \text{and} \quad \beta = \frac{\mathrm{DA_{accept}}}{\mathrm{AA_{accept}}} \quad \text{(Equation 8 \& 9)}$$

Correction terms were determined for three ROIs in three images of single measurements. Using the average correction terms, the corrected FRET efficiency was determined per ROI in the images of samples with both donor and acceptor. For each salt of interest, samples were prepared in triplicate and for each replicate three images were recorded, for which three ROIs were measured, resulting in 27 measurements per salt of interest.

3. Supporting Data

3.1. General supporting data

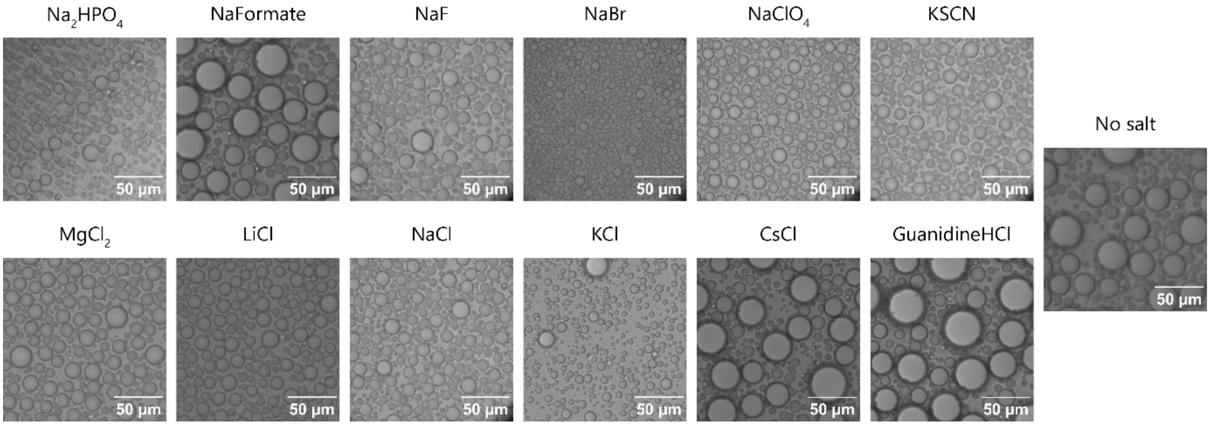

Supporting Figure 2: Brightfield microscopy images of 1 mM protamine / 25 mM ATP condensates in presence of 100 mM of different monovalent salts, 50 mM of divalent salts (molecule-based, see Supporting Information Section 2.1) and in absence of salt show that in all cases the condensates are liquid droplets that fuse rapidly.

3.2. Supporting data 'Selective binding of ions to condensate components'

3.2.1. Binding to the α-proton

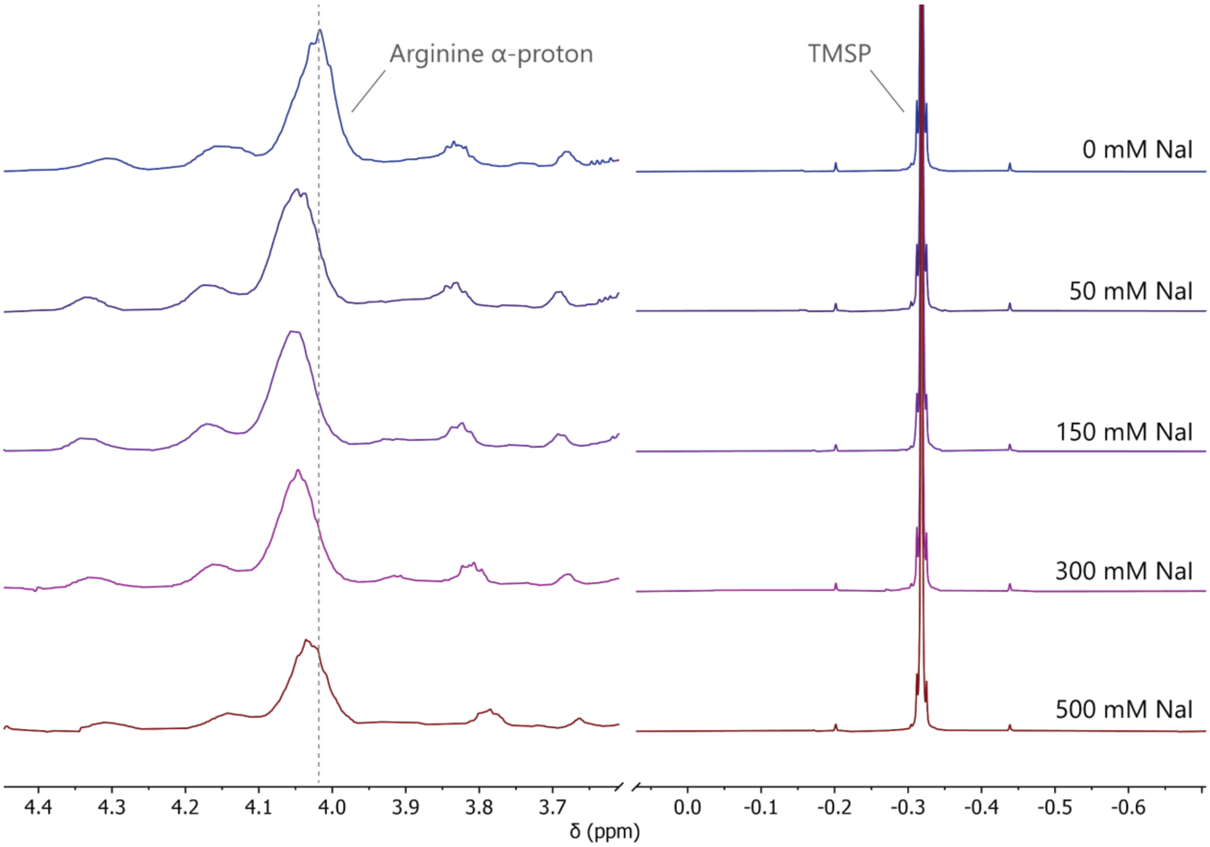

Supporting Figure 3: ^{1}H-NMR-spectra for binding of NaI to the α-proton of the arginines on 1 mM protamine in D_2O pD 8.5 with 5 mM (3-(trimethylsilyl)propionic-2,2,3,3-d_4 acid (TMSP) in D_2O as internal standard in the inner tube of an NMR coaxial tube, measured at 5˚C. Full NMR spectra and spectra for other salts can be found on the Radboud Data Repository (Section 5).

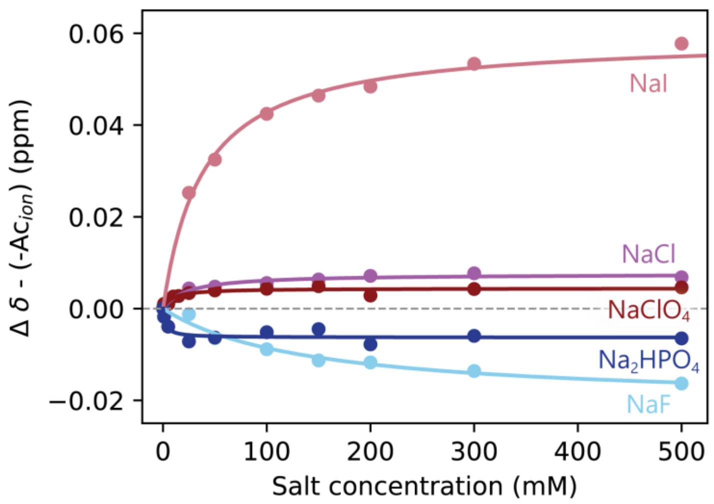

Supporting Figure 4: Fitted binding curves for the non-linear part $\Delta\delta - (-Ac_{\text{ion}}) = \frac{\Delta\delta_{\max} c_{\text{ion}}}{K_{\text{D,app}} + c_{\text{ion}}}$ for binding of different anions to the α-proton of the arginines on 1 mM protamine at 5˚C. The difference in sign of $\Delta\delta$-($-Ac_{\text{ion}}$) between the chaotropes and kosmotropes likely indicates a different mechanism of binding.

Supporting Table 4: Fitted $K_{\text{D,app}}$ of Equation 1 for binding of different anions to the α-proton of the arginines on 1 mM protamine at 5˚C. *Because the sample already contains 23.1 mM Cl^- at 0 mM added salt, this amount is included in the $K_{\text{D,app}}$.

**Salt**	**$K_{\text{D,app}}$ (mM)**
Na_2HPO_4	2.4 ± 1.7
NaF	140.0 ± 96.7
NaCl	46.3 ± 10.7*
NaI	38.2 ± 5.4
$NaClO_4$	8.6 ± 3.5

3.2.2. Binding to $^{15}N_2$-guanido-labeled arginine

Supporting Table 5: Fitted $K_{\text{D,app}}$ of Equation 1 for binding of different anions to the guanidinium nitrogens of $^{15}N_2$-guanido-labeled arginine at 25˚C. *Because the sample already contains 61.8 mM Cl^- at 0 mM added salt, this amount is included in the $K_{\text{D,app}}$.

**Salt**	**$K_{\text{D,app}}$ (mM)**
Na_2HPO_4	114.9 ± 23.2
NaF	1786.7 ± 94881.2 (Non-binding)
NaCl	310.7 ± 150.4*
NaI	229.2 ± 185.0
$NaClO_4$	7.6 ± 26.5

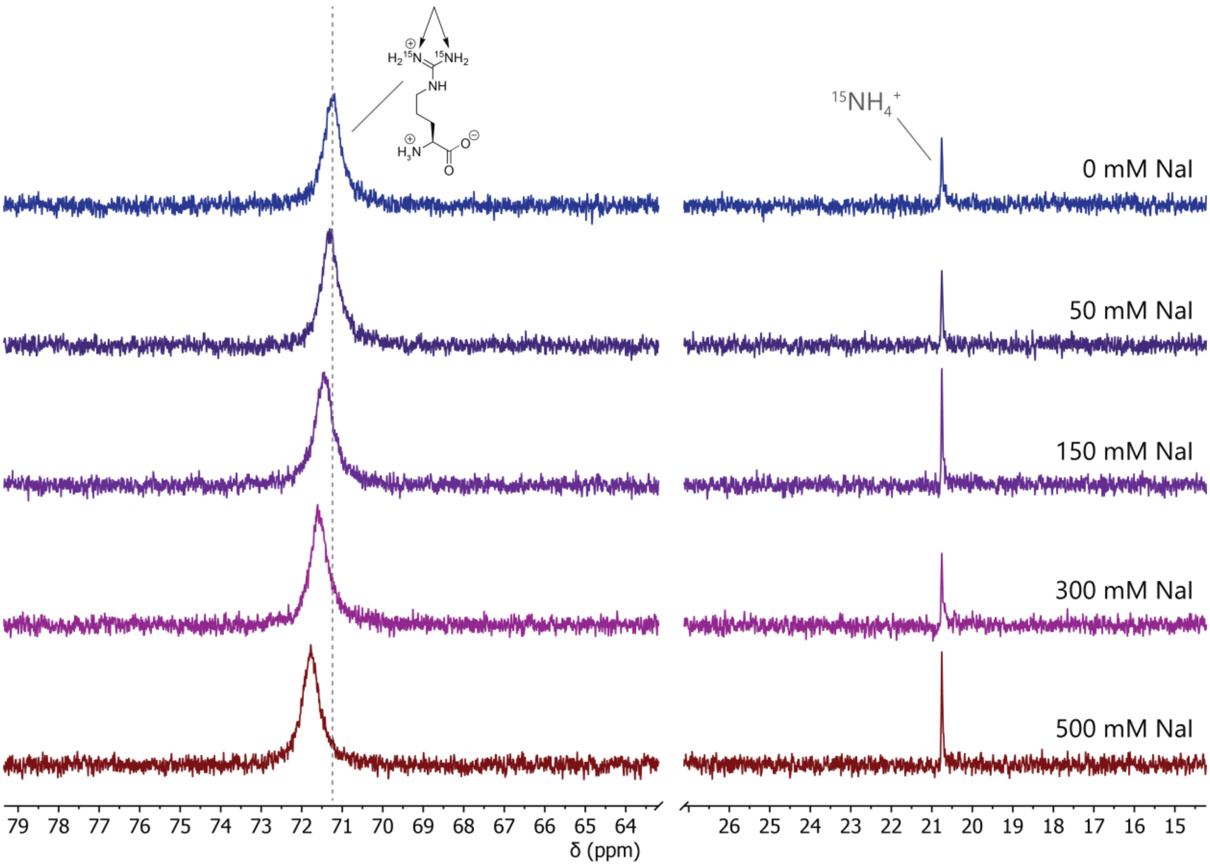

Supporting Figure 5: ^{15}N-NMR-spectra for binding of NaI to the guanidinium nitrogens of $^{15}N_2$-guanido-labeled arginine in 50 mM Tris pH 8.5 in 9 : 1 H_2O : D_2O, with 100 mM ^{15}N-ammonium chloride in 9 : 1 H_2O : D_2O as internal standard in the inner tube of an NMR coaxial tube, measured at 25˚C. Full NMR spectra and spectra for other salts can be found on the Radboud Data Repository (Section 5).

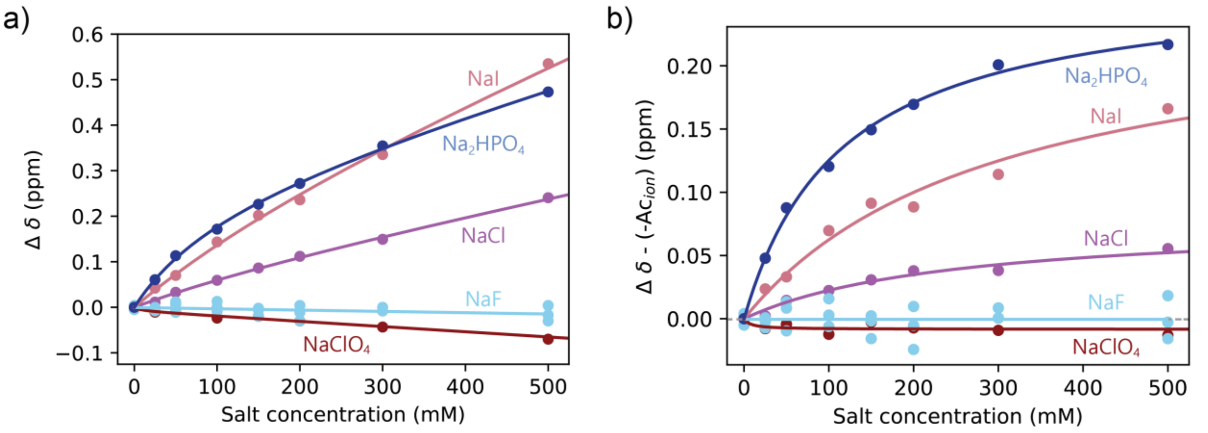

Supporting Figure 6: a) Fitted binding curves for binding of sodium salts of different anions to the guanidinium nitrogens of $^{15}N_2$-guanido-labeled arginine at 25˚C. **b)** Fitted binding curves for the non-linear part $\Delta\delta - (-Ac_{\text{ion}}) = \frac{\Delta\delta_{\text{max}}\, c_{\text{ion}}}{K_{\text{D,app}} + c_{\text{ion}}}$ for binding of different anions to the guanidinium nitrogens of $^{15}N_2$-guanido-labeled arginine at 25˚C. The difference in sign of $\Delta\delta$-($-Ac_{\text{ion}}$) between different ions likely indicates a different mechanism of binding.

3.2.3. Binding to ATP

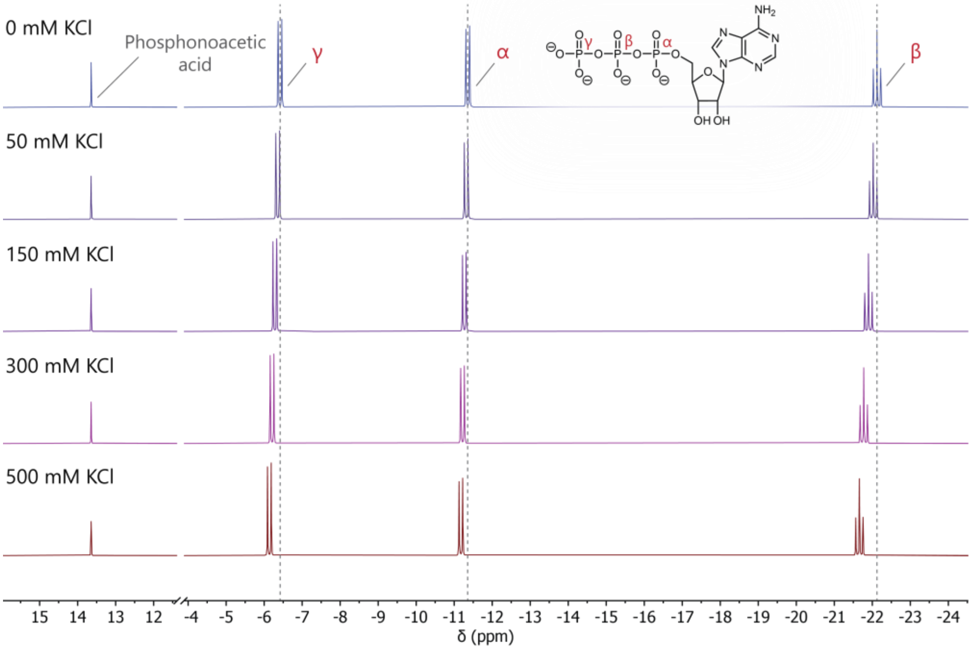

Supporting Figure 7: ^{31}P-NMR-spectra for binding of KCl to the phosphates on 25 mM ATP in 50 mM Tris pH 8.5 in 9 : 1 H_2O : D_2O, with 20 mM phosphonoacetic acid in 9 : 1 H_2O : D_2O as internal standard in the inner tube of an NMR coaxial tube, measured at 5˚C. Full NMR spectra and spectra for other salts can be found on the Radboud Data Repository (Section 5). In the analysis for Figure 1g, the $K_{D,app}$'s of the α-, β- and γ-phosphate were averaged for binding to the phosphates.

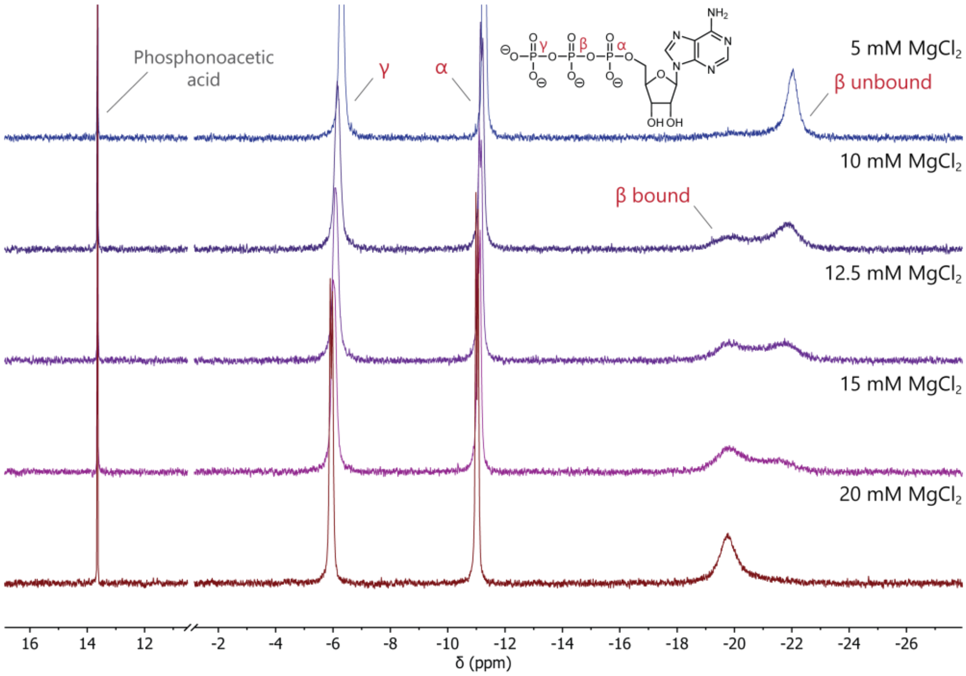

Supporting Figure 8: ^{31}P-NMR-spectra for $MgCl_2$ binding to the phosphates on ATP, showing two distinct peaks of the β-phosphate between 5 and 20 mM. Spectra were obtained for 25 mM ATP in 50 mM Tris pH 8.5 in 9 : 1 H_2O : D_2O, with 20 mM phosphonoacetic acid in 9 : 1 H_2O : D_2O as internal standard in the inner tube of an NMR coaxial tube, measured at 5˚C.

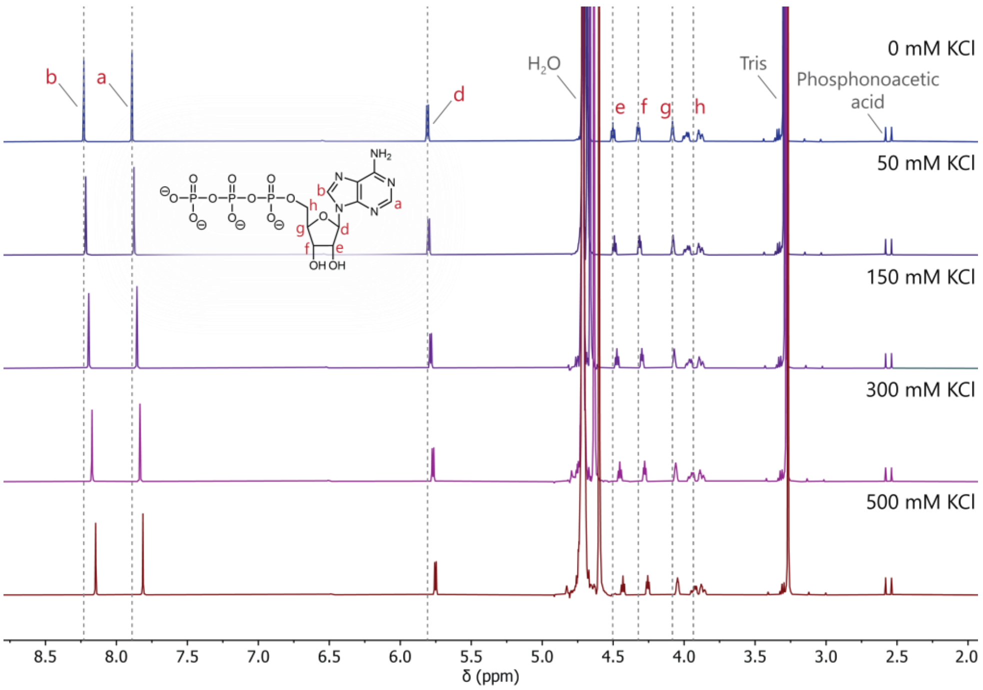

Supporting Figure 9: ^{1}H-NMR-spectra for binding of KCl to the ribose and nucleobase protons on 25 mM ATP in 50 mM Tris pH 8.5 in 9 : 1 H_2O : D_2O, with 20 mM phosphonoacetic acid in 9 : 1 H_2O : D_2O as internal standard in the inner tube of an NMR coaxial tube, measured at 5˚C. Full NMR spectra and spectra for other salts can be found on the Radboud Data Repository (Section 5). Two peaks are observed for H_2O, as the water in the inner coaxial tube has a different environment than the water in the outer coaxial tube. In the analysis for Figure 1g, the $K_{D,app}$'s of the *a* and *b* protons were averaged for binding to the nucleobase, and the $K_{D,app}$'s of the *d*, *e* and *f* protons were averaged for binding to the sugar.

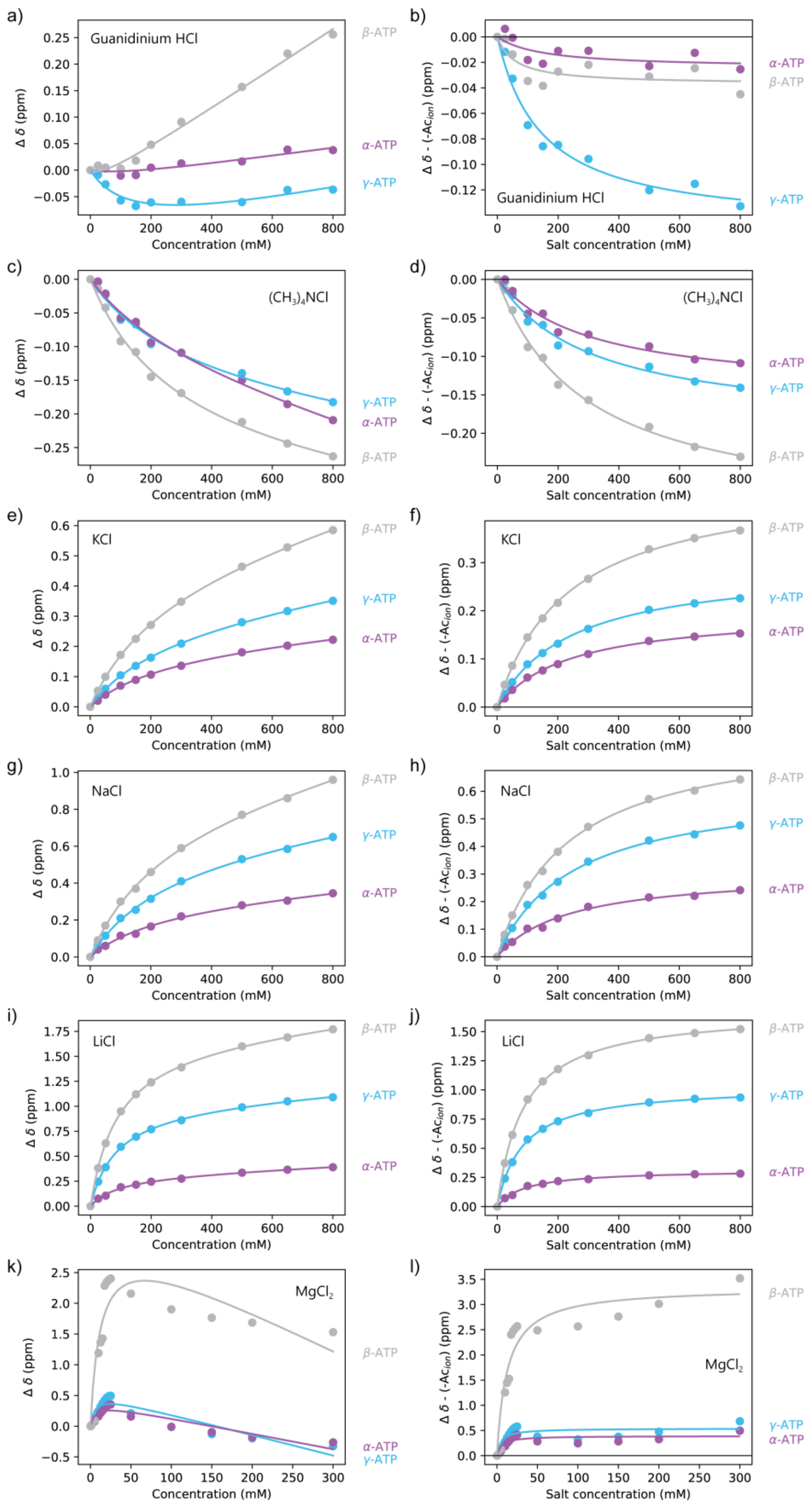

Supporting Figure 10: a,c,e,g,i,k) Fitted binding curves for binding of chloride salts of different cations to the phosphates on 25 mM ATP at 5˚C. **b,d,f,h,j,l)** Fitted binding curves for the non-linear part $\Delta\delta \; - (-Ac_{\text{ion}}) = \frac{\Delta\delta_{\max} c_{\text{ion}}}{K_{\text{D,app}} + c_{\text{ion}}}$ for binding of chloride salts of different cations to the phosphates on 25 mM ATP at 5˚C. The difference in sign of Δδ-(-Ac_{ion}) between different ions likely indicates a different mechanism of binding.

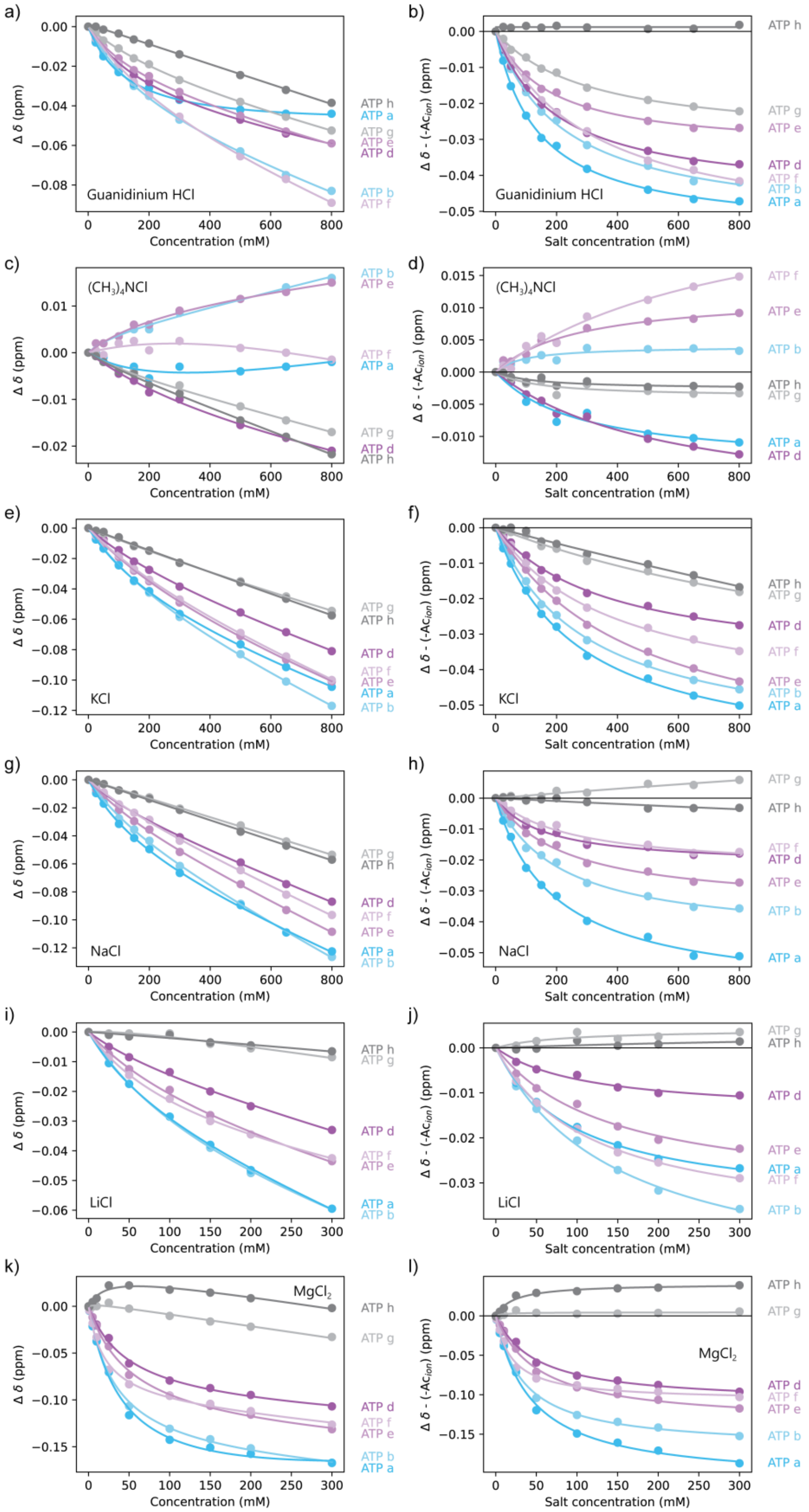

Supporting Figure 11: a,c,e,g,i,k) Fitted binding curves for binding of chloride salts of different cations to the ribose (purple, grey) and nucleobase (blue) on 25 mM ATP at 5˚C. Grey binding curves were not taken into account for the average $K_{D,app}$ of the ribose. **b,d,f,h,j,l)** Fitted binding curves for the non-linear part of the binding curve. The difference in sign of $\Delta\delta$-(-Ac_{ion}) between different ions likely indicates a different mechanism of binding.

Supporting Table 6: Average fitted $K_{D,app}$ of Equation 1 for binding of different cations to the nucleobase, ribose and phosphate on 25 mM ATP at 5˚C. *Because the sample already contains 92.9 mM Na^+ at 0 mM added salt, this amount is included in the $K_{D,app}$.

Salt	Avg. $K_{D,app}$ nucleobase (mM)	Avg. $K_{D,app}$ ribose (mM)	Avg. $K_{D,app}$ phosphate (mM)
Guanidine HCl	180.6	229.2	116.8
$(CH_3)_4NCl$	184.8	548.6	302.8
KCl	286.9	407.2	238.6
NaCl	297.1*	306.9*	331.7*
LiCl	123.7	122.9	87.1
$MgCl_2$	39.1	40.4	7.9

3.2.4. Binding to $(GHGLY)_3$

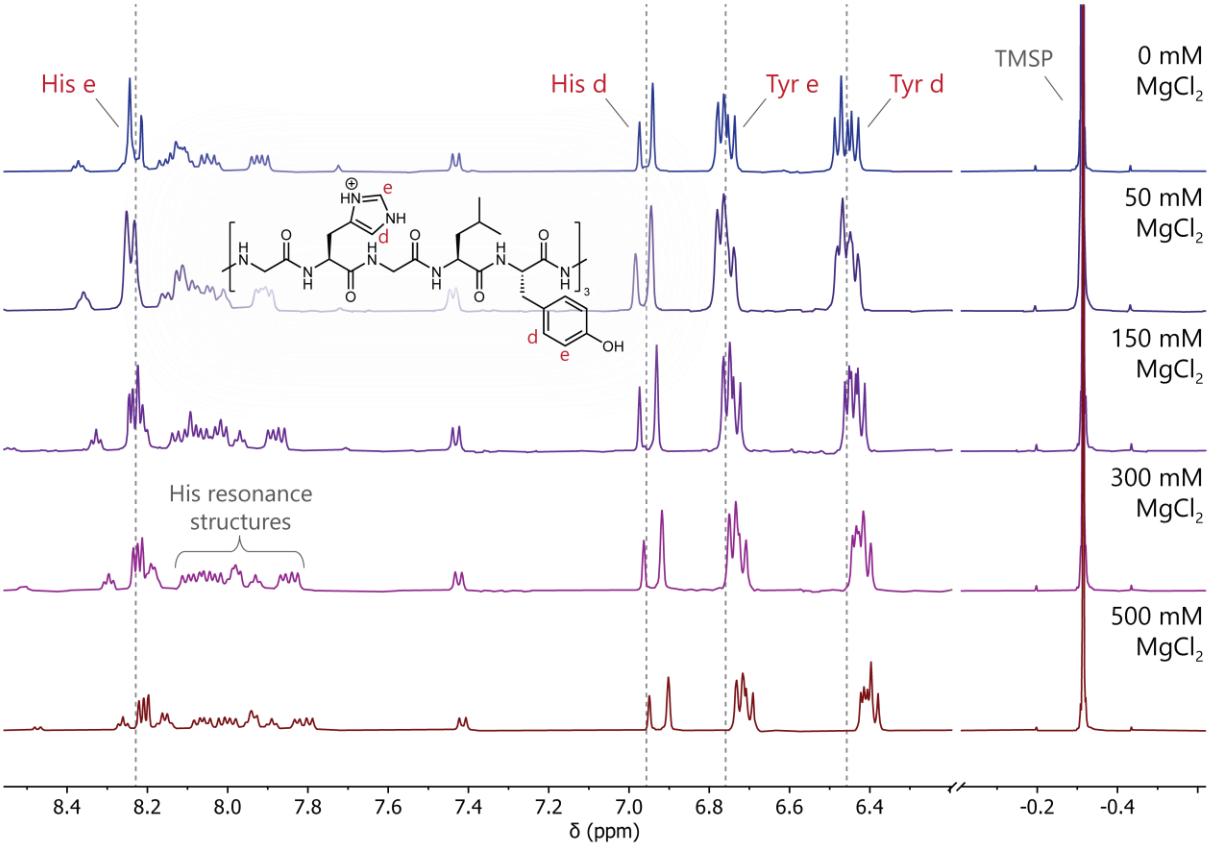

Supporting Figure 12: ^{1}H-NMR-spectra for binding of $MgCl_2$ to the tyrosine and histidine aromatic ring protons on 1 mM $(GHGLY)_3$ in 50 mM acetate pH 5.0 in 9 : 1 H_2O : D_2O, with 5 mM (3-(trimethylsilyl)propionic-2,2,3,3-d_4 acid (TMSP) in D_2O as internal standard in the inner tube of an NMR coaxial tube, measured at 5˚C. The peaks at δ = 7.8 – 8.15 are different resonance structures of histidine. These would not be visible for free histidine in solution, as they would interconvert rapidly. Therefore, this indicates that the peptide forms a hydrophobic cluster in solution. For our binding analysis, we only took the peaks of histidine into account that correspond with the expected peaks for free histidine in solution. Full NMR spectra, full characterization and spectra for other salts can be found on the Radboud Data Repository (Section 5).

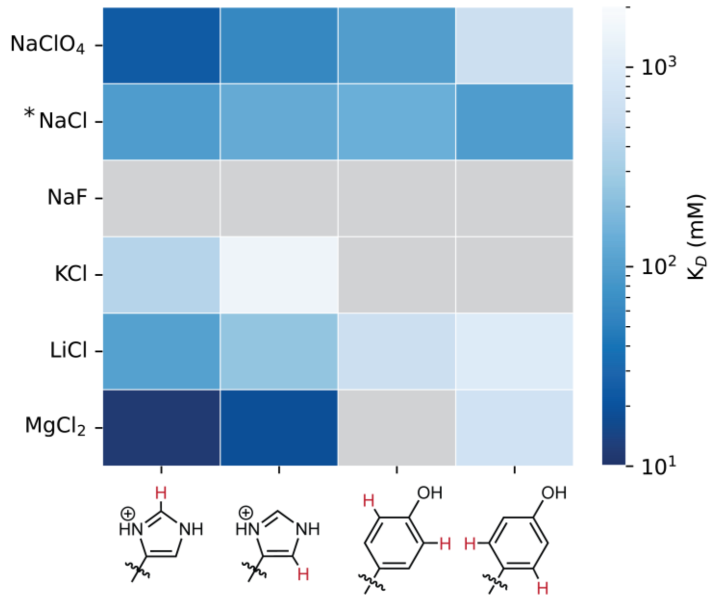

Supporting Figure 13: Apparent dissociation constants of anions and cations for the histidine and tyrosine of $(GHGLY)_3$. Kosmotropic cations and chaotropic anions bind most strongly to histidine. We observe weak binding of cations to tyrosine. For the grey squares the binding curves could not be fit accurately. *Because the sample already contains 14.4 mM Cl^- at 0 mM added salt, this amount was added to the $K_{D,app}$.

Supporting Table 7: Average fitted $K_{D,app}$ of Equation 1 for binding of different cations and anions to the tyrosine and histidine on 1 mM $(GHGLY)_3$ at 5˚C. *Because the sample already contains 50 mM Na^+ and 14.4 mM Cl^- at 0 mM added salt, these amounts were included in the $K_{D,app}$'s.

Salt	$K_{D,app}$ His e (mM)	$K_{D,app}$ His d (mM)	$K_{D,app}$ Tyr e (mM)	$K_{D,app}$ Tyr d (mM)
$NaClO_4$	23.6 ± 13.4	58.8 ± 8.4	97.1 ± 12.9	611.3 ± 555.5
NaCl*	93.5 ± 37.6 (Cl^-)<br>129.8 ± 37.6 (Na^+)	127.9 ± 57.9 (Cl^-)<br>163.5 ± 57.9 (Na^+)	142.7 ± 111.5 (Cl^-)<br>178.3 ± 111.5 (Na^+)	95.3 ± 132.4 (Cl^-)<br>130.9 ± 132.4 (Na^+)
NaF	Poor fit	Poor fit	Poor fit	Poor fit
KCl	410.0 ± 271.3	1467.4 ± 2718.4	Poor fit	Poor fit
LiCl	105.3 ± 64.3	241.1 ± 189.7	608.1 ± 1053.6	991.0 ± 3412.5
$MgCl_2$	11.5 ± 11.5	18.3 ± 20.6	Poor fit	692.0 ± 11781.7

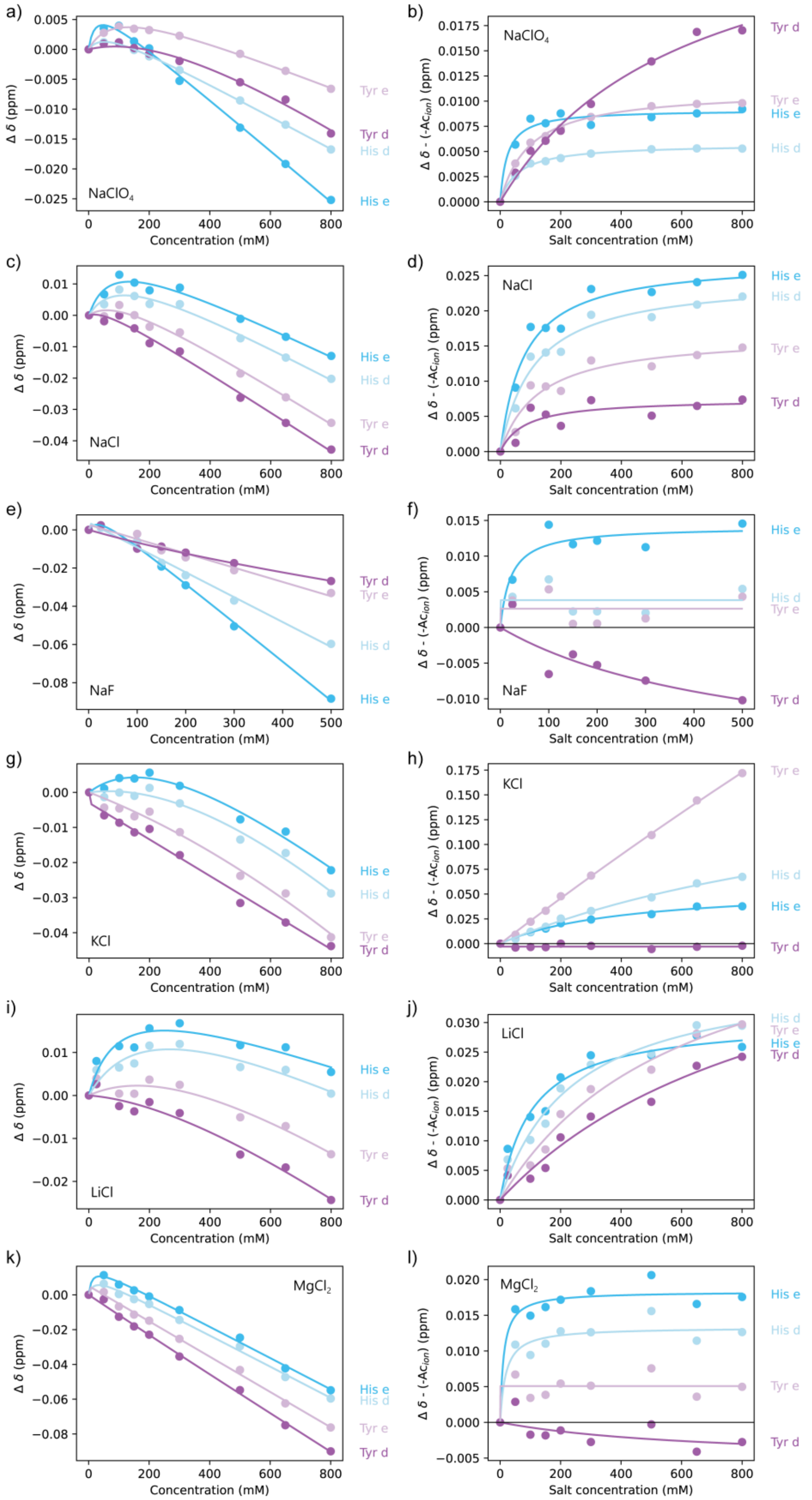

Supporting Figure 14: a,c,e,g,i,k) Fitted binding curves for binding of sodium salts of different anions and chloride salts of different cations to the tyrosine (purple) and histidine (blue) on 1 mM $(GHGLY)_3$ at 5˚C. **b,d,f,h,j,l)** Fitted binding curves for the non-linear part of the binding curve. The difference in sign of Δδ-(-Ac$_{ion}$) between different ions likely indicates a different mechanism of binding. The binding curves of NaF and the binding curves of KCl with Tyr and $MgCl_2$ with Tyr e could not be fit accurately, and were therefore left out of the analysis.

3.3. Supporting data 'Ion binding is sequence specific and compacts protamine'

3.3.1. Supporting data MD-simulations

3.3.1.1. Simulations with unbiased configuration

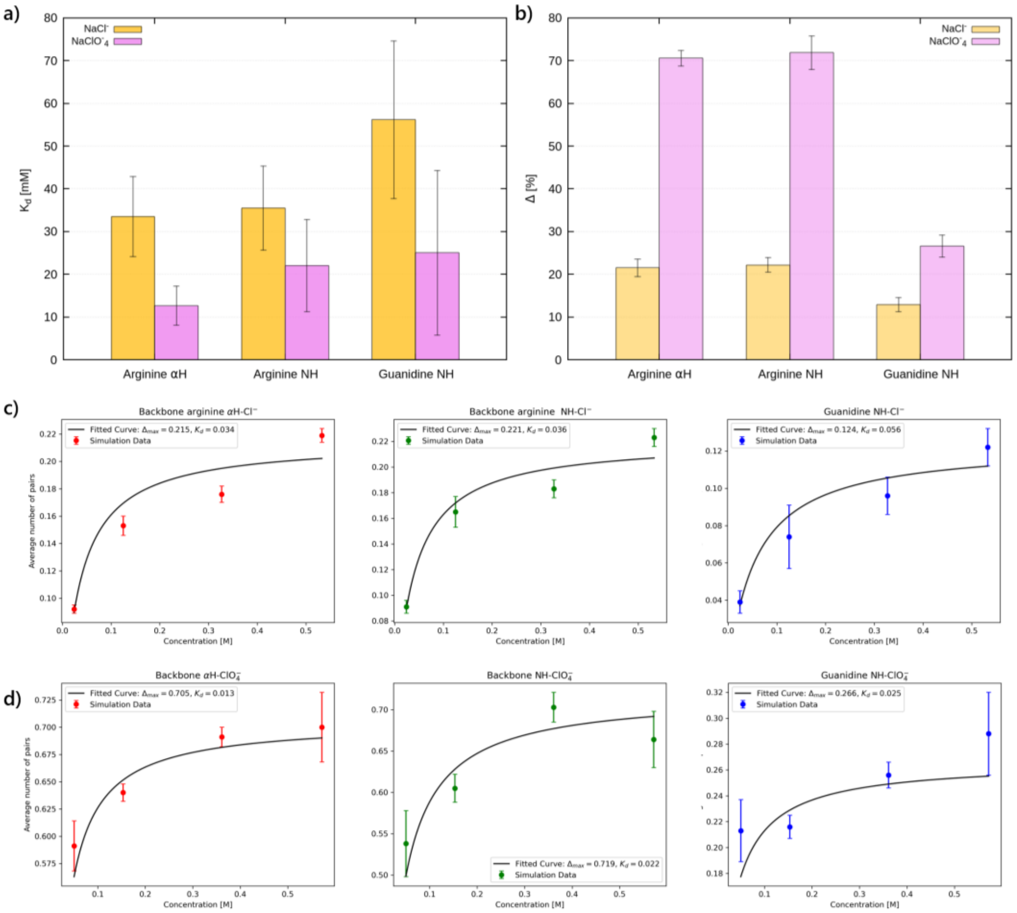

Supporting Figure 15: Determination of dissociation constants from ion-binding MD simulations. **a)** K_D of the protamine hydrogen-ion interactions fitted via the isothermal binding curve from the MD simulations at different concentrations. The obtained K_D's show stronger binding to the backbone than to the arginine guanidinium, in accordance with the NMR binding data. **b)** The second parameter of the fit, Δ, which quantifies the asymptotic occupancy level, i.e. the saturation of binding sites, shows that ClO_4^- ions bind more strongly to the backbone and guanidine compared to the Cl^-. **c, d)** Plots of the fitted binding curves and simulation data for **c)** Cl^- and **d)** ClO_4^-. The binding does not saturate at high salt concentrations. Due to the fluctuation between a predominant open form and less prevalent closed state (Supporting Information Figure 17a), not all binding sites are simultaneously available.

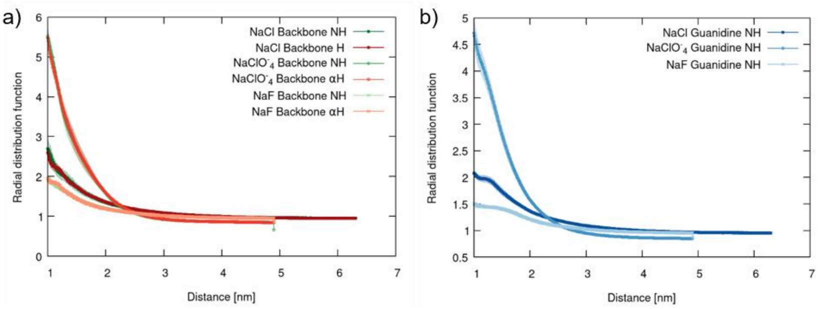

Supporting Figure 16: At large distances the radial distribution functions (RDF's) converge to unity, showing that there is no accumulation of ions at the edge of the simulation box.

3.3.1.2. Simulations with fixed configuration

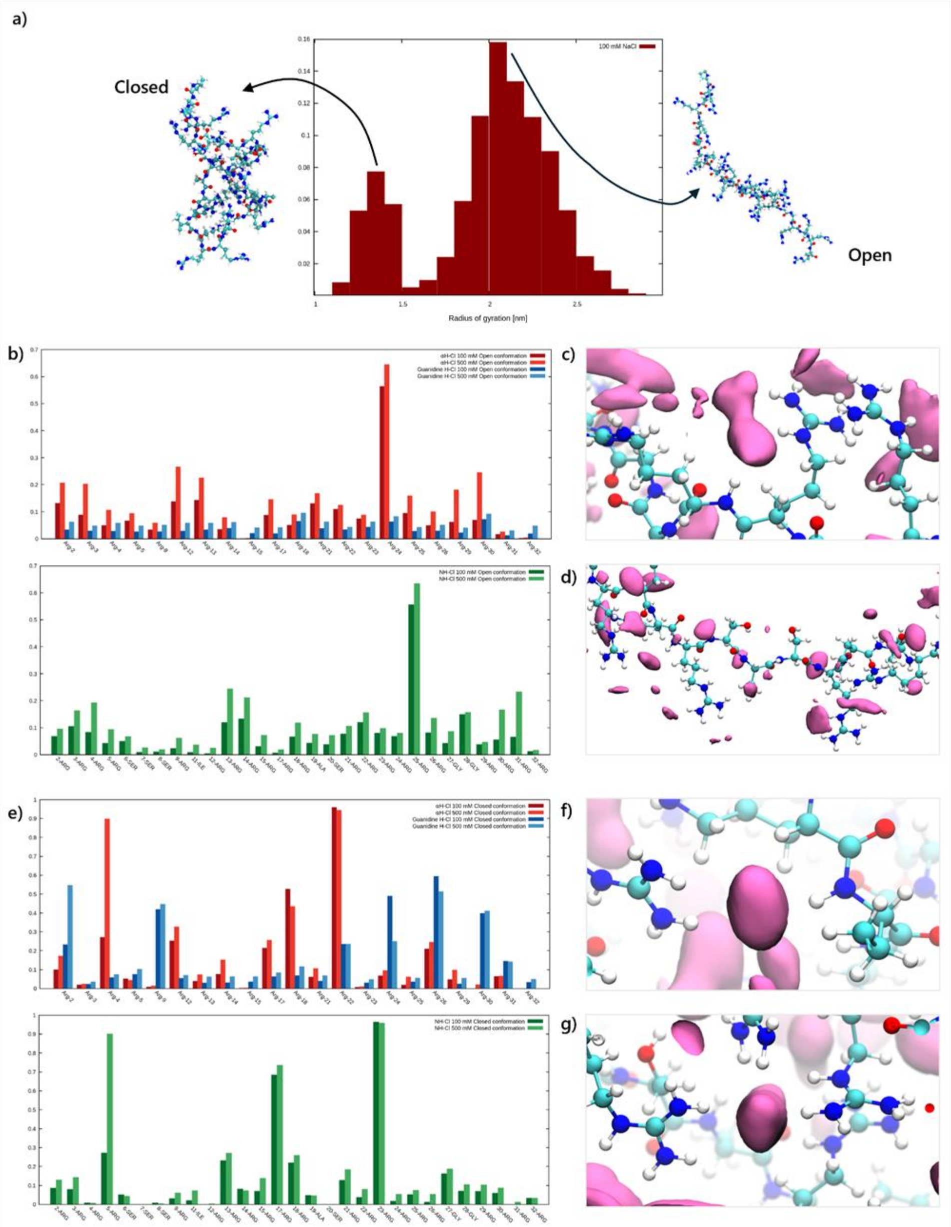

Supporting Figure 17: MD simulations for protamine in a fixed open or closed configuration. **a)** Example distribution of the radius of gyration of protamine, showing a distinct open and closed population. The peptide is represented with white hydrogen, cyan carbon, blue nitrogen and red oxygen atoms. **b)** Per-residue contact analysis for the open configuration: arginine α-protons and guanidine NH (top) and backbone NH (bottom). Contacts are significantly reduced compared to the unbiased simulations for all backbone residues except the α-proton and NH pair of residues 24 and 25. **c)** Snapshot of the open configuration showing the region with the highest ion density, corresponding to arginines 24 and 25, showing that the guanidine of residue 25 is oriented in such a way that the ions interacting with them are also interacting with the backbone hydrogens of residue 24, creating a binding pocket. The mauve solids represent isosurfaces with isovalue 40× the bulk ion concentration. **d)** Snapshot of the open configuration, showing the Ser-rich region of protamine. The serines are depleted of ions compared to the surrounding arginine-rich regions. **e)** Per-residue contact analysis for the closed configuration: arginine α-protons and guanidine NH (top) and backbone NH (bottom). Both the backbone and guanidines show heterogeneous ion affinities. **f)** Snapshot of the closed configuration showing a region of high ion density at the guanidine of residue 9 and the backbone hydrogen pair formed by the α-proton of residue 22 and the NH group of residue 23. **g)** Snapshot of the closed configuration showing a region of high ion density at the guanidines of residues 22, 26 and 30.

3.3.2. Supporting data SAXS measurements

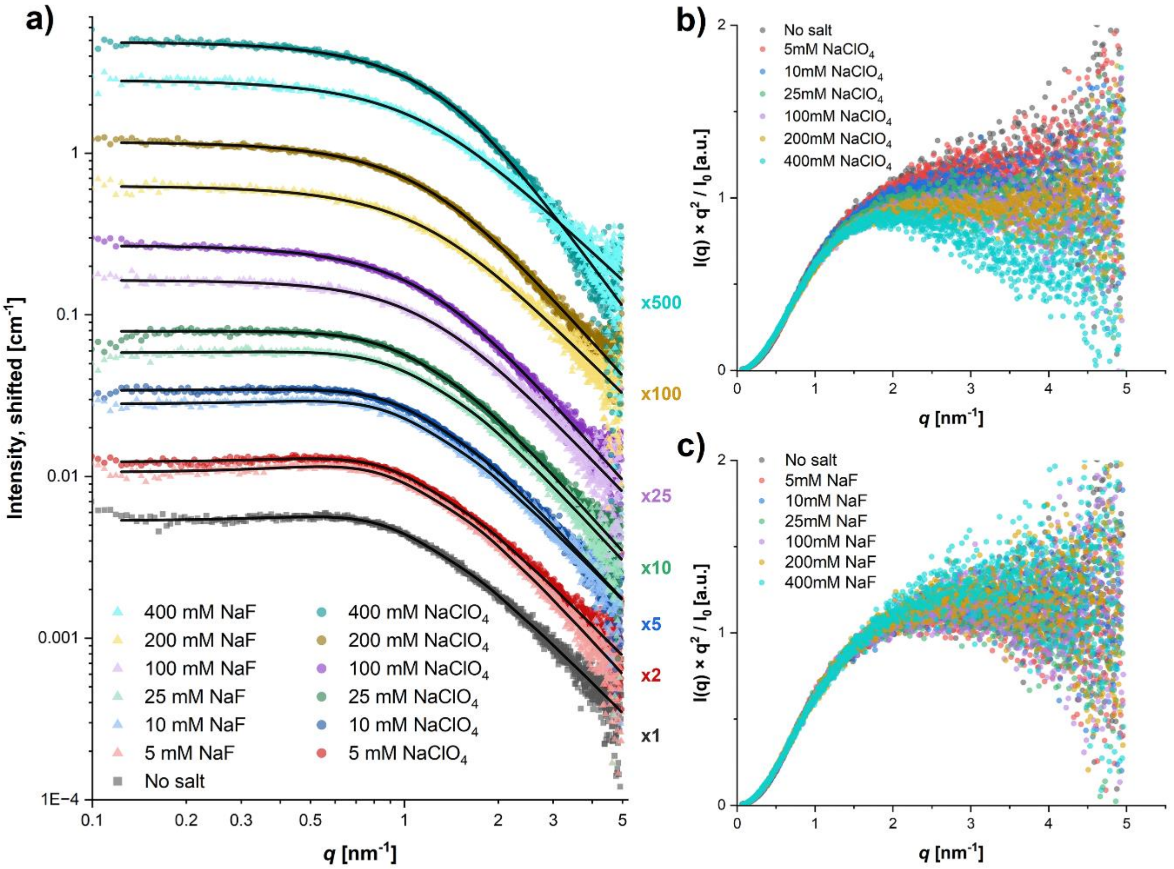

Supporting Figure 18: Obtained SAXS curves for 1 mM protamine with different concentrations of $NaClO_4$ or NaF. **a)** SAXS curves fitted with generalized gaussian coil form factor, including a hard sphere structure factor to account for repulsion between peptides. Curves are displayed shifted by the factors shown on the right to avoid overlap. **b)** Kratky plot for different concentrations of $NaClO_4$ (Figure 2i in main text), showing a clear compaction of the protamine for higher $NaClO_4$ concentrations. **c)** Kratky plot for different concentrations of NaF, showing that the addition of NaF does not have a pronounced effect on the protamine conformation.

Supporting Table 8: Parameters obtained from fitted SAXS curves of 1 mM (4 mg/mL) protamine with different concentrations of $NaClO_4$.

**[$NaClO_4$] (mM)**	**Radius of gyration R_g (nm)**	**Flory excluded volume parameter ν**	**Forward scattering I_0 (cm^{-1})**	**Effective hard sphere radius R_{HS} (nm)**	**Effective volume fraction ϕ_{HS}**
0	1.275 ± 0.002	0.507 ± 0.010	0.0070 ± 0.0013	3.30 ± 0.01	0.033 ± 0.019
5	1.275 ± 0.002	0.504 ± 0.008	0.0080 ± 0.0012	3.24 ± 0.01	0.033 ± 0.016
10	1.263 ± 0.002	0.468 ± 0.008	0.0084 ± 0.0012	3.17 ± 0.01	0.025 ± 0.016
25	1.283 ± 0.002	0.456 ± 0.007	0.0092 ± 0.0011	3.35 ± 0.01	0.018 ± 0.018
100	1.319 ± 0.002	0.454 ± 0.007	0.0109 ± 0.0014	3.08 ± 0.19	0.001 ± 0.061
200	1.323 ± 0.002	0.459 ± 0.008	0.01179 ± 0.00209	2.52 ± 9.02	0.001 ± 0.095
400	1.272 ± 0.002	0.379 ± 0.011	0.00978 ± 0.00207	-	0.000

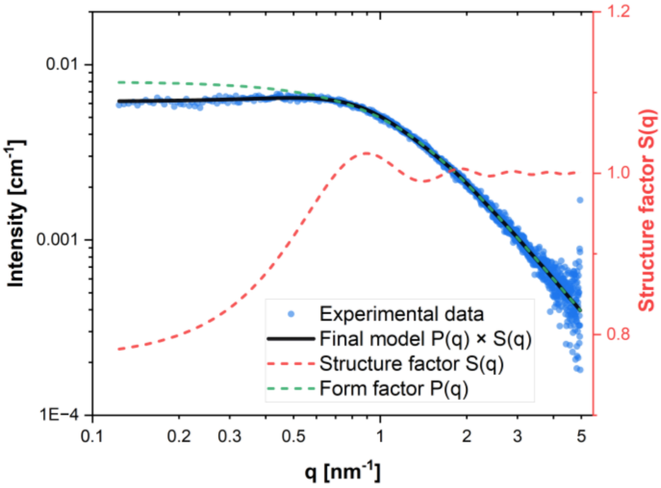

Supporting Figure 19: Influence of the structure factor and form factor on the final model SAXS curve at different q values. We did not observe a change in radius of gyration (Supporting Table 8), which is likely due to the scattering curves being significantly affected by interparticle interactions at lower q values, where also the information about radius of gyration lies. Those interparticle interactions (coming from strong electrostatic repulsion between highly cationic protamine chains) are accounted for by a hard sphere structure factor component in the SAXS model as described in Supporting Information Section 2.5. The SAXS model is a product of this structure factor with the form factor, containing information about the shape and size of the particles. It can be seen that at higher q values (where the information about the scaling exponent is extracted), the structure factor approaches unity, barely affecting the final model. Therefore, we can assume that the values found for the scaling exponent (Flory parameter) are accurate and less prone to distortions due to interparticle repulsion than the radius of gyration is.

3.4. Supporting data ‘Ion partitioning into condensates follows LMWA’

3.4.1. NMR calibration curves

3.4.1.1. NMR Calibration curves $^7Li^+$

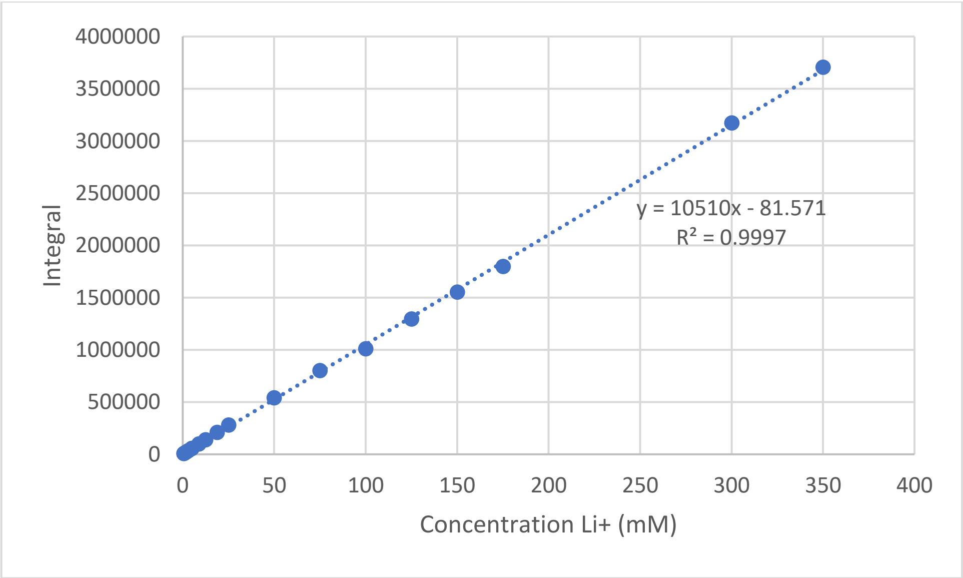

Supporting Figure 20: ^{7}Li-NMR calibration curve for 0.5 – 350 mM lithium chloride, used for determining the dilute phase Li^+ concentration. P1 = 12.15 µs, d1 = 200 s, rg = 1030, ns = 8. See Supporting Information Section 1.3 for full description of settings.

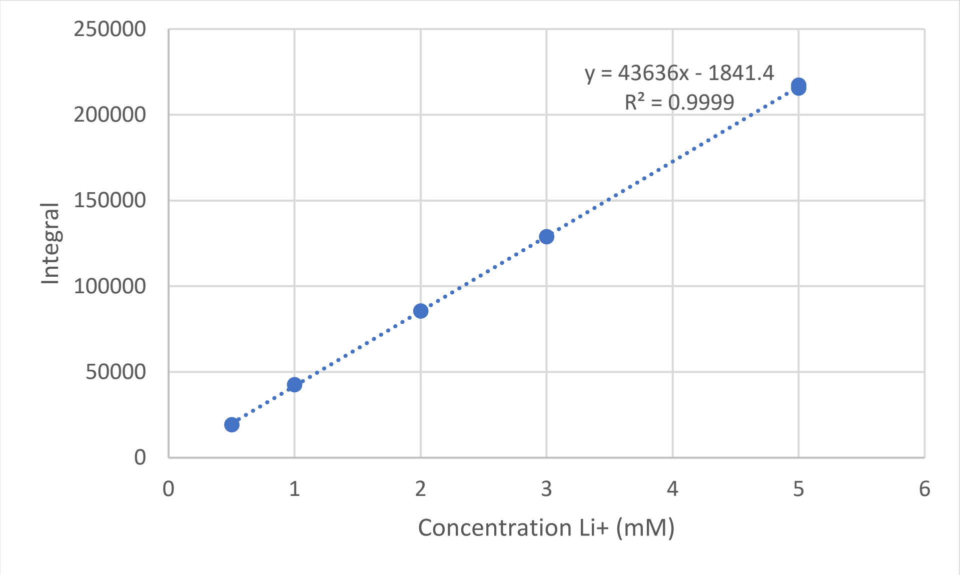

Supporting Figure 21: ^{7}Li-NMR calibration curve for 0.5 – 5 mM lithium chloride, used for determining the condensate phase Li^{+} concentration. P1 = 12.15 µs, d1 = 200 s, rg = 2050, ns = 16. See Supporting Information Section 1.3 for full description of settings.

3.4.1.2. NMR Calibration curves $^{23}Na^{+}$

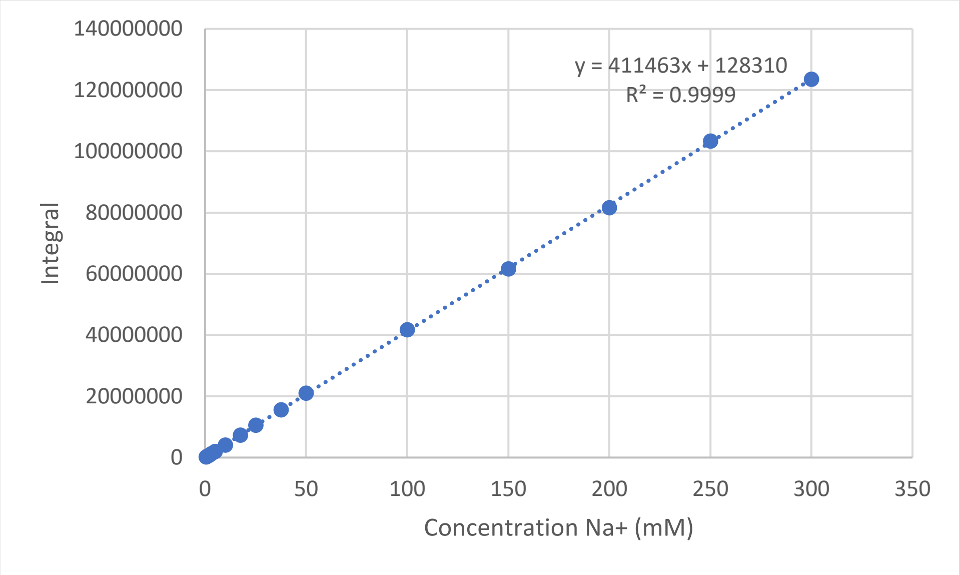

Supporting Figure 22: ^{23}Na-NMR calibration curve for 0.5 – 300 mM sodium chloride, used for determining the dilute phase Na^{+} concentration. P1 = 20.5 µs, d1 = 2 s, rg = 1820, ns = 1024. See Supporting Information Section 1.3 for full description of settings.

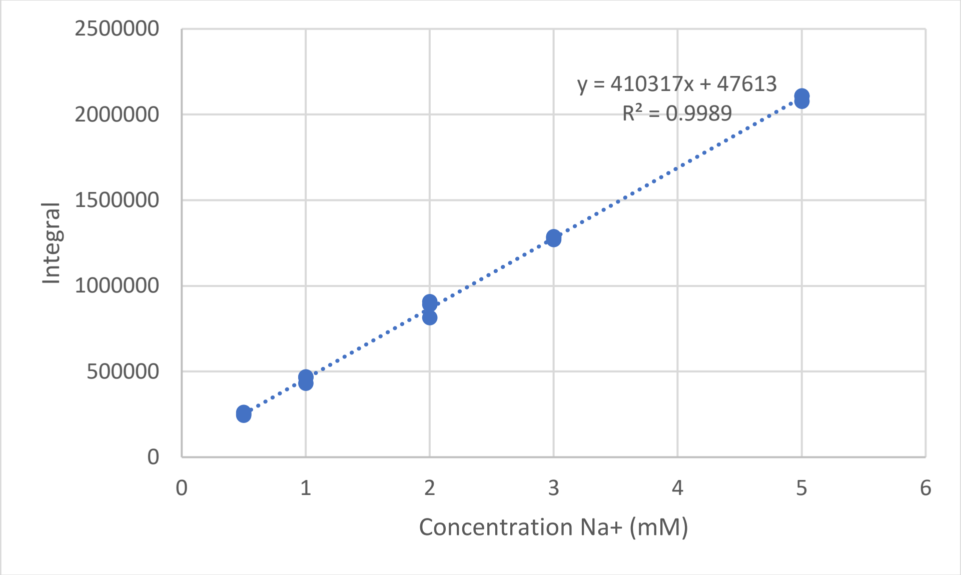

Supporting Figure 23: ^{23}Na-NMR calibration curve for 0.5 – 5 mM sodium chloride, used for determining the condensate phase Na^{+} concentration. P1 = 20.5 µs, d1 = 2 s, rg = 1820, ns = 1024. See Supporting Information Section 1.3 for full description of settings.

3.4.1.3. NMR Calibration curves $^{25}Mg^{2+}$

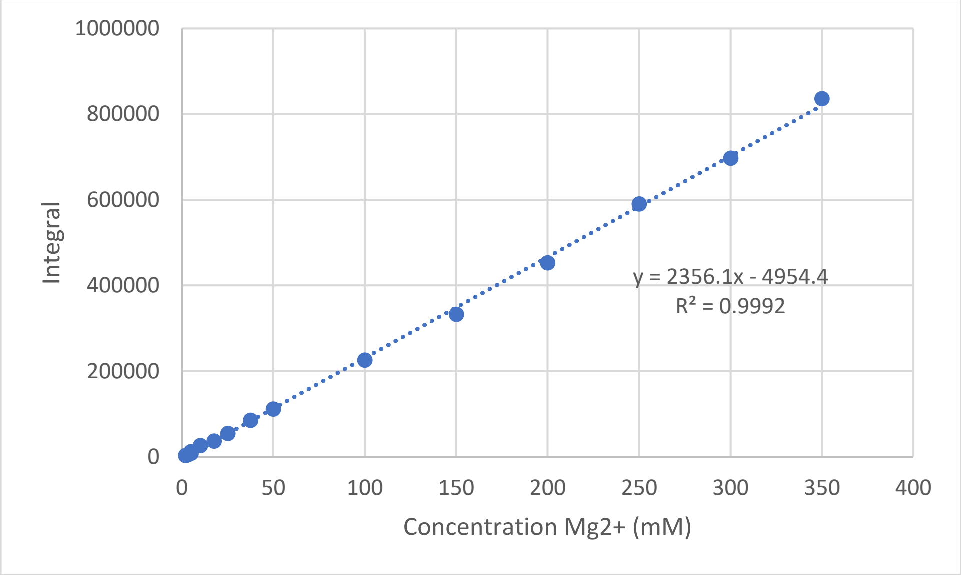

Supporting Figure 24: ^{25}Mg-NMR calibration curve for 0.5 – 350 mM magnesium chloride (molecule-based, see Supporting Information Section 2.1), used for determining the dilute phase Mg^{2+} concentration. P1 = 20.0 µs, d1 = 0.1 s, rg = 203, ns = 4096. See Supporting Information Section 1.3 for full description of settings.

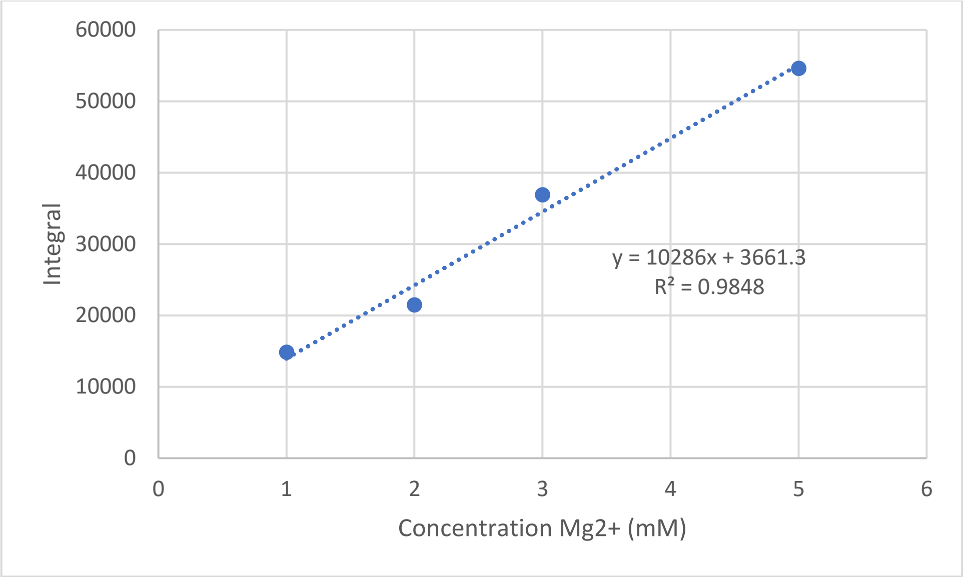

Supporting Figure 25: ^{25}Mg-NMR calibration curve for 1 – 5 mM magnesium chloride (molecule-based, see Supporting Information Section 2.1), used for determining the condensate phase Mg^{2+} concentration. P1 = 20.0 µs, d1 = 0.1 s, rg = 203, ns = 20480. See Supporting Information Section 1.3 for full description of settings.

3.4.1.4. NMR Calibration curves $^{35}Cl^{-}$

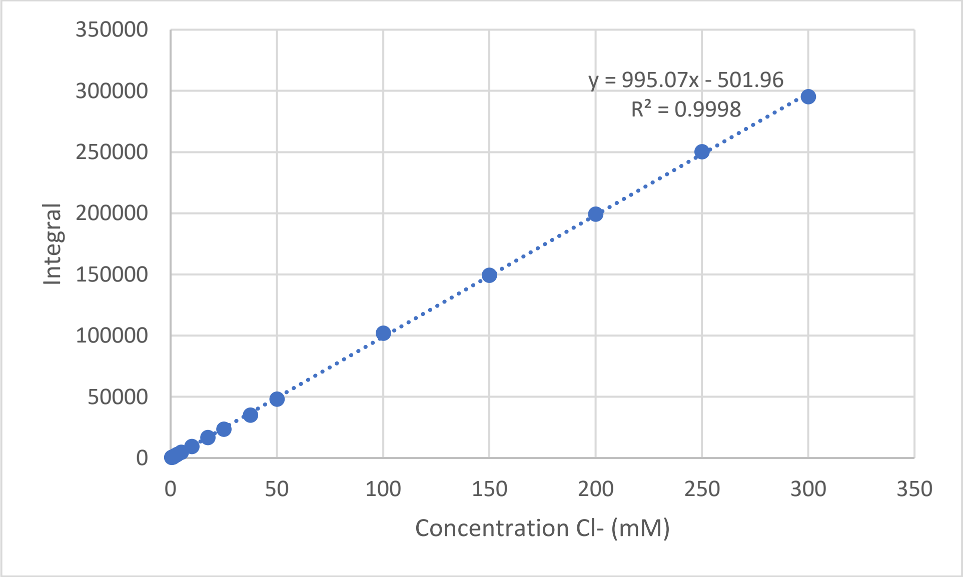

Supporting Figure 26: ^{35}Cl-NMR calibration curve for 0.5 – 300 mM sodium chloride, used for determining the dilute phase Cl^{-} concentration. P1 = 15.5 µs, d1 = 2 s, rg = 50.8, ns = 512. See Supporting Information Section 1.3 for full description of settings.

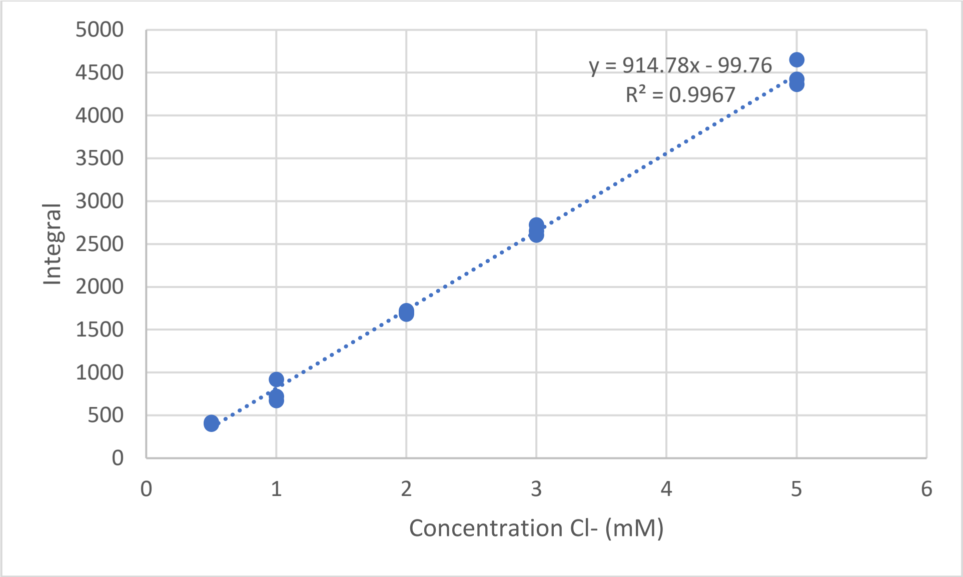

Supporting Figure 27: ^{35}Cl-NMR calibration curve for 0.5 – 5 mM sodium chloride, used for determining the condensate phase Cl^- concentration. P1 = 15.5 µs, d1 = 2 s, rg = 50.8, ns = 512. See Supporting Information Section 1.3 for full description of settings.

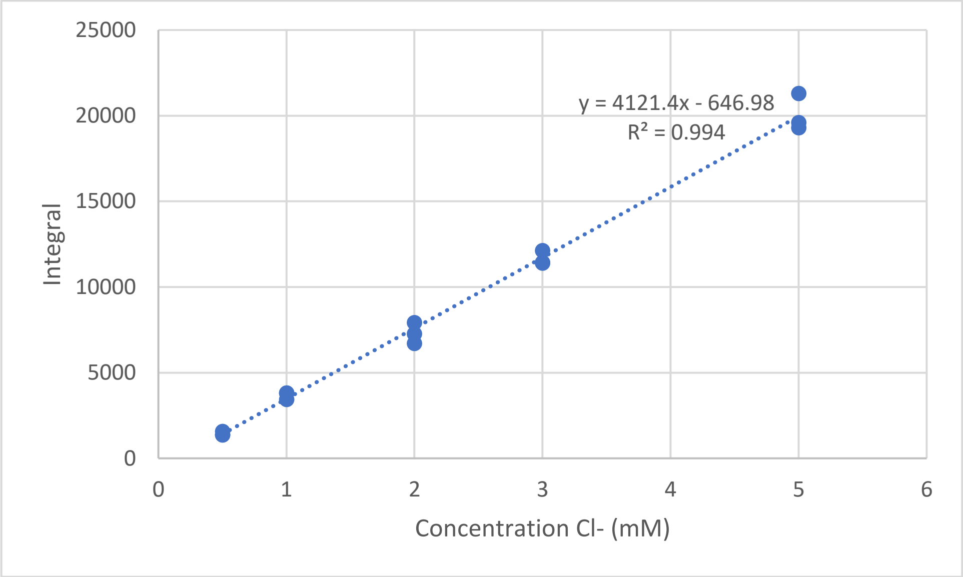

Supporting Figure 28: ^{35}Cl-NMR calibration curve for 0.5 – 5 mM sodium chloride, used for determining the condensate phase Cl^- concentration. P1 = 15.5 µs, d1 = 2 s, rg = 64, ns = 2048. See Supporting Information Section 1.3 for full description of settings.

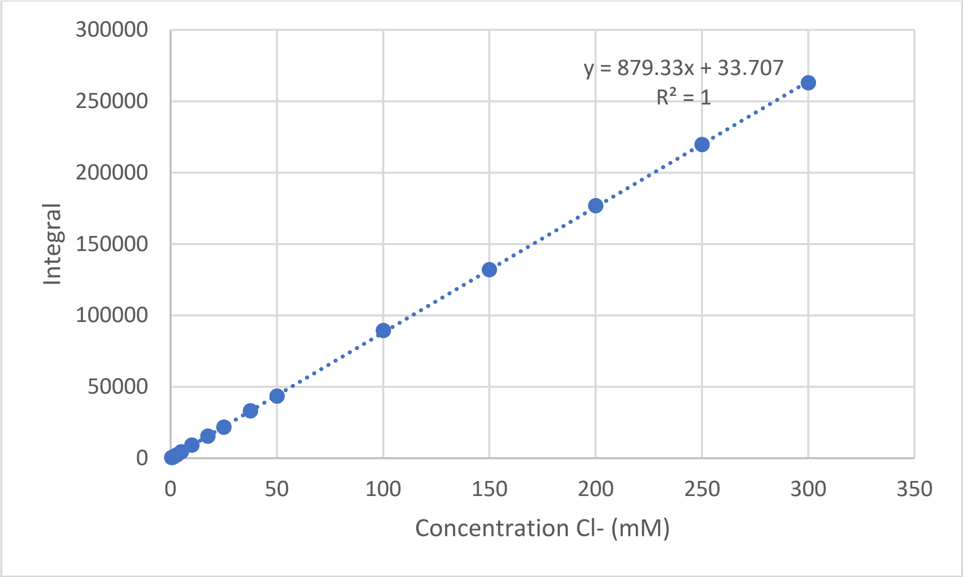

Supporting Figure 29: ^{35}Cl-NMR calibration curve for 0.5 – 300 mM sodium chloride, used for determining the dilute phase Cl^- concentration in samples with ClO_4^-. P1 = 15.5 µs, d1 = 2 s, sw = 1195 ppm, O1P = 500 ppm, rg = 50.8, ns = 1024. See Supporting Information Section 1.3 for full description of settings.

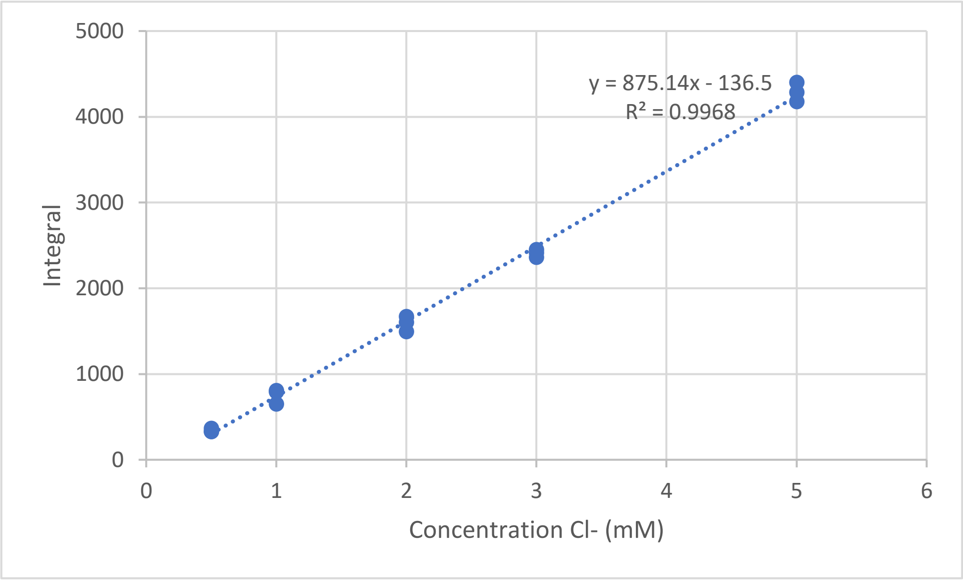

Supporting Figure 30: ^{35}Cl-NMR calibration curve for 0.5 – 5 mM sodium chloride, used for determining the condensate phase Cl^- concentration in samples with ClO_4^-. P1 = 15.5 µs, d1 = 2 s, sw = 1195 ppm, O1P = 500 ppm, rg = 50.8, ns = 1024. See Supporting Information Section 1.3 for full description of settings.

3.4.1.5. NMR Calibration curves $^{35}ClO_4^-$

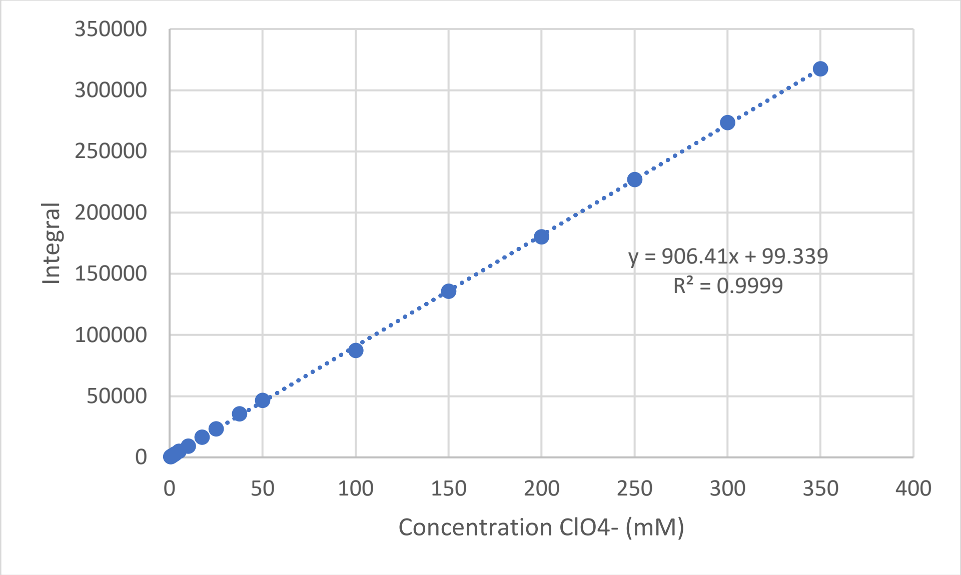

Supporting Figure 31: ^{35}Cl-NMR calibration curve for 0.5 – 350 mM sodium perchlorate, used for determining the dilute phase ClO_4^- concentration. P1 = 15.5 µs, d1 = 2 s, sw = 1195 ppm, O1P = 500 ppm, rg = 50.8, ns = 1024. See Supporting Information Section 1.3 for full description of settings.

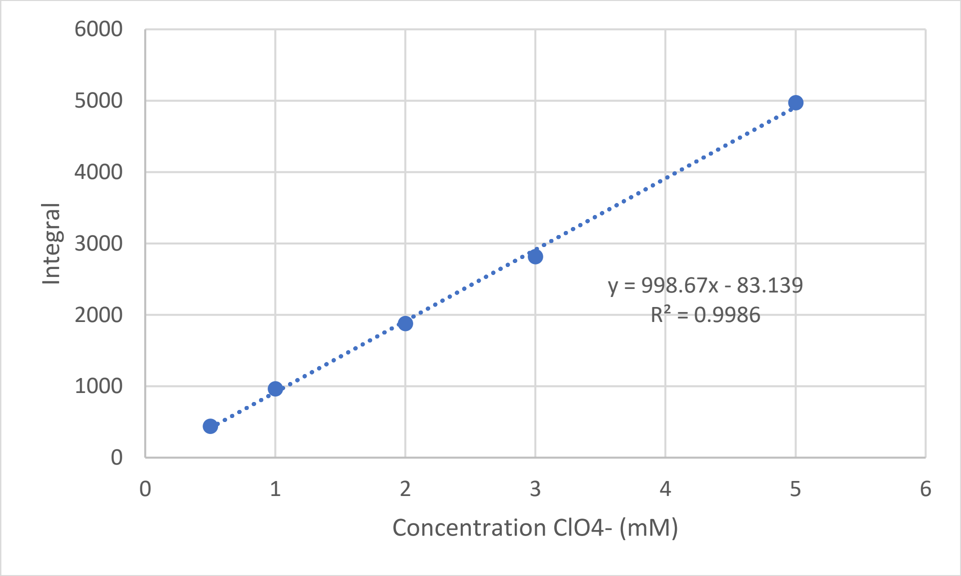

Supporting Figure 32: ^{35}Cl-NMR calibration curve for 0.5 – 5 mM sodium perchlorate, used for determining the condensate phase ClO_4^- concentration. P1 = 15.5 µs, d1 = 2 s, sw = 1195 ppm, O1P = 500 ppm, rg = 50.8, ns = 1024. See Supporting Information Section 1.3 for full description of settings.

3.4.1.6. NMR Calibration curves $^{39}K^+$

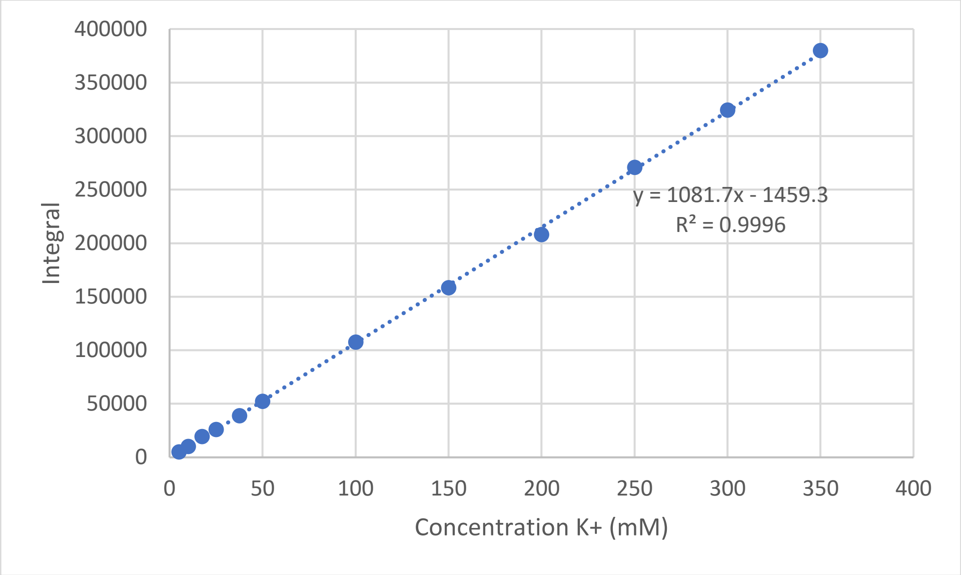

Supporting Figure 33: ^{39}K-NMR calibration curve for 0.5 – 350 mM potassium chloride, used for determining the dilute phase K^+ concentration. P1 = 25.0 µs, d1 = 0.1 s, rg = 101, ns = 4096. See Supporting Information Section 1.3 for full description of settings.

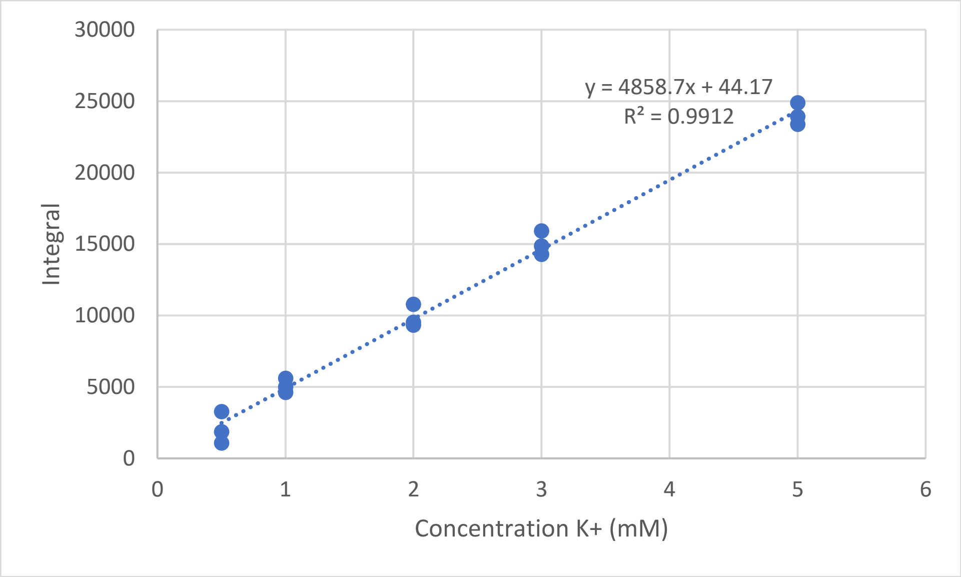

Supporting Figure 34: ^{39}K-NMR calibration curve for 0.5 – 5 mM potassium chloride, used for determining the condensate phase K^+ concentration. P1 = 25.0 µs, d1 = 0.1 s, rg = 203, ns = 20480. See Supporting Information Section 1.3 for full description of settings.

3.4.1.7. NMR Calibration curves $^{81}Br^-$

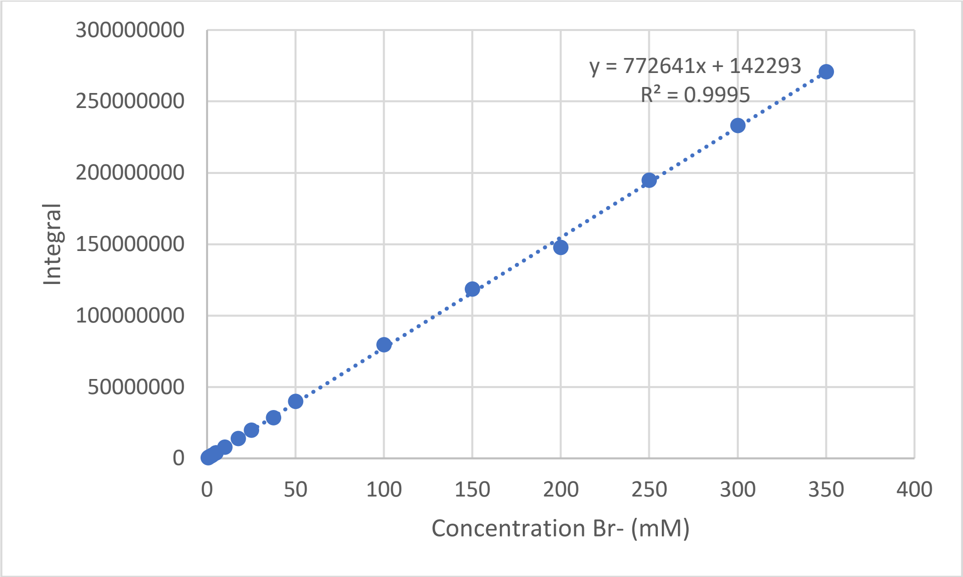

Supporting Figure 35: ^{81}Br-NMR calibration curve for 0.5 – 350 mM sodium bromide, used for determining the dilute phase Br^- concentration. P1 = 12.0 µs, d1 = 1 s, rg = 2050, ns = 2048. See Supporting Information Section 1.3 for full description of settings.

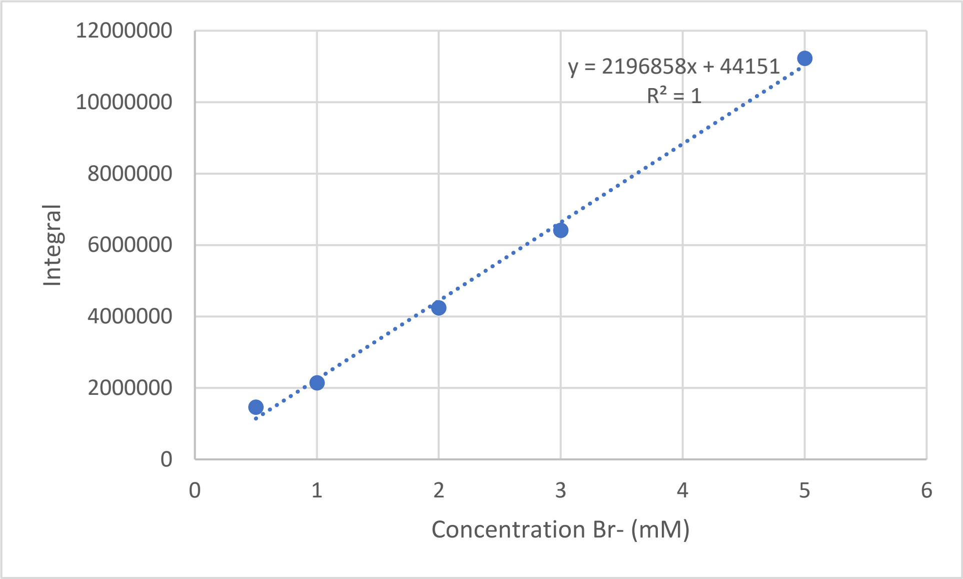

Supporting Figure 36: ^{81}Br-NMR calibration curve for 0.5 – 5 mM sodium bromide, used for determining the condensate phase Br^- concentration. P1 = 12.0 µs, d1 = 1 s, rg = 2050, ns = 6144. See Supporting Information Section 1.3 for full description of settings.

3.4.1.8. NMR Calibration curves $^{133}Cs^{+}$

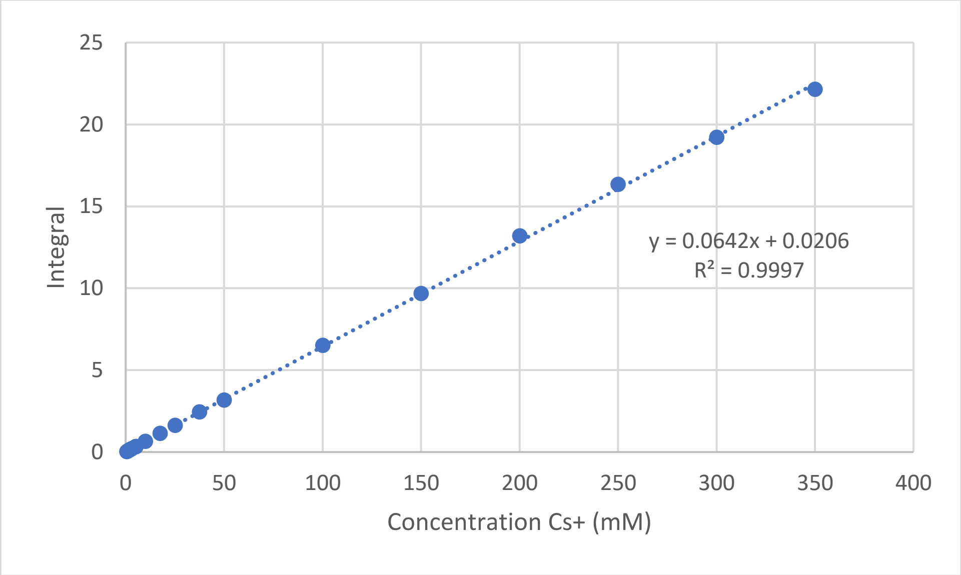

Supporting Figure 37: ^{133}Cs-NMR calibration curve for 0.5 – 350 mM cesium chloride, used for determining the dilute phase Cs^{+} concentration. P1 = 7.5 µs, d1 = 23 s, rg = 50, ns = 32. See Supporting Information Section 1.3 for full description of settings.

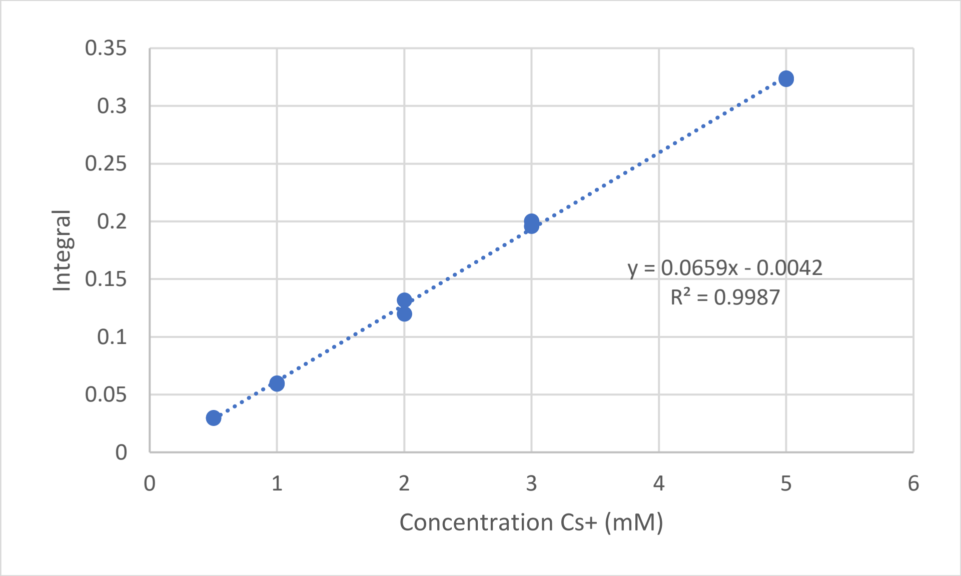

Supporting Figure 38: ^{133}Cs-NMR calibration curve for 0.5 – 5 mM cesium chloride, used for determining the condensate phase Cs^{+} concentration. P1 = 7.5 µs, d1 = 23 s, rg = 50, ns = 32. See Supporting Information Section 1.3 for full description of settings.

3.4.2. NMR spectra and measured concentrations ion partitioning

In this section we show an example of the ^{1}H, ^{7}Li, ^{13}C, ^{19}F, ^{23}Na, ^{31}P, ^{35}Cl, ^{39}K, ^{81}Br and ^{133}Cs-NMR measurements we performed to determine the concentrations of protamine, ATP, Tris and different anions and cations in the condensate and dilute phase. The full set of raw NMR spectra can be found on the Radboud Data repository (Section 5).

3.4.2.1. No salt

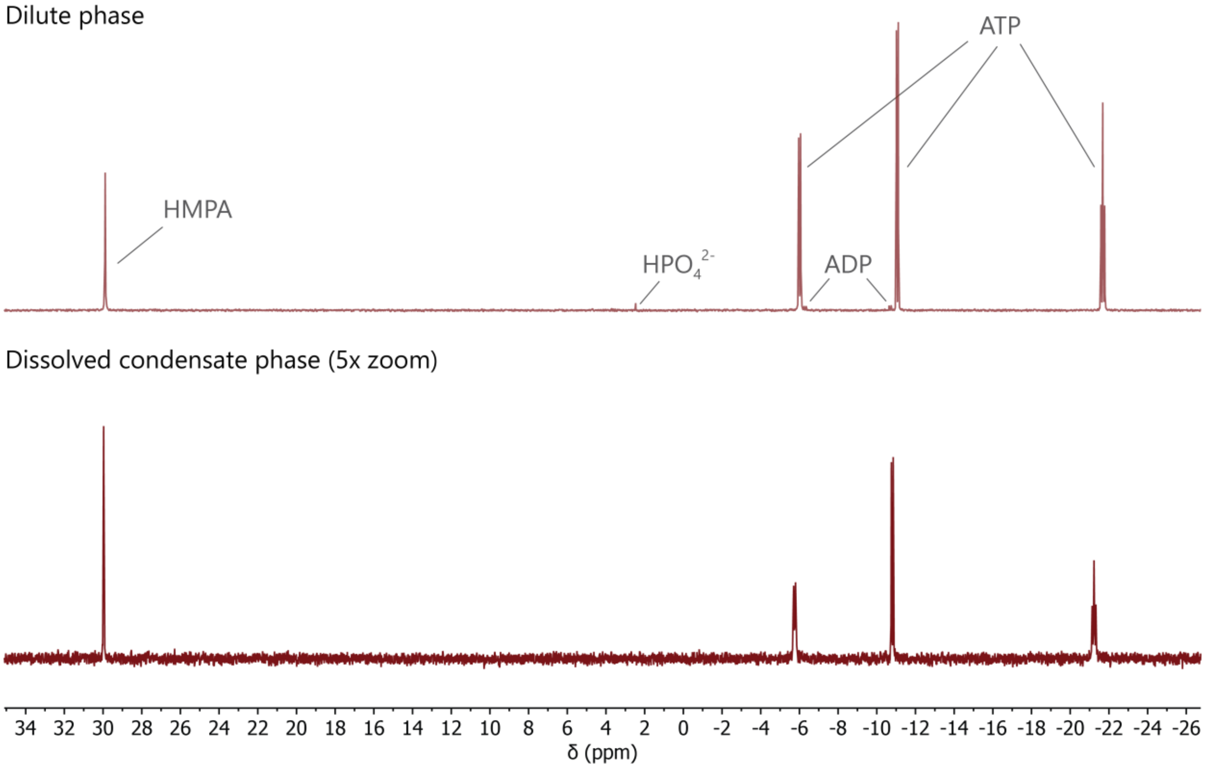

Supporting Figure 39: ^{31}P-NMR-spectra of the dilute phase and dissolved condensate phase for condensates of 1 mM protamine chloride / 25 mM ATP without added salt in 50 mM Tris pH 8.5, used to determine the concentration of ATP. HMPA is the hexamethylphosphoramide internal standard. Only very minor peaks for ADP and phosphate are observed, showing that no significant hydrolysis of ATP takes place during the sample preparation.

Supporting Table 9: Measured charge-based concentrations in the condensate phase and dilute phase and K_P for condensates without added salt. Charge-based means that the protamine concentration represents the concentration of arginines (21 per protamine), rather than the concentration of peptide. Similarly, for ATP the charge-based concentration should be divided by four to obtain the molecule concentration. For monovalent ions the charge-based concentration equals the molecule concentration.

**Compound**	$c_{condensate}$ **(mM)**	c_{dilute} **(mM)**	K_P
Protamine	2191.3 ± 36.3	6.65 ± 0.05	329.5 ± 5.9
ATP	2340.1 ± 57.8	82.5 ± 0.9	28.4 ± 0.8
Tris	65.1 ± 0.7	52.5 ± 0.4	1.24 ± 0.02
Na^+	183.3 ± 5.8	94.2 ± 0.6	1.95 ± 0.06
Cl^-	161.0 ± 15.1	41.7 ± 0.8	3.86 ± 0.37

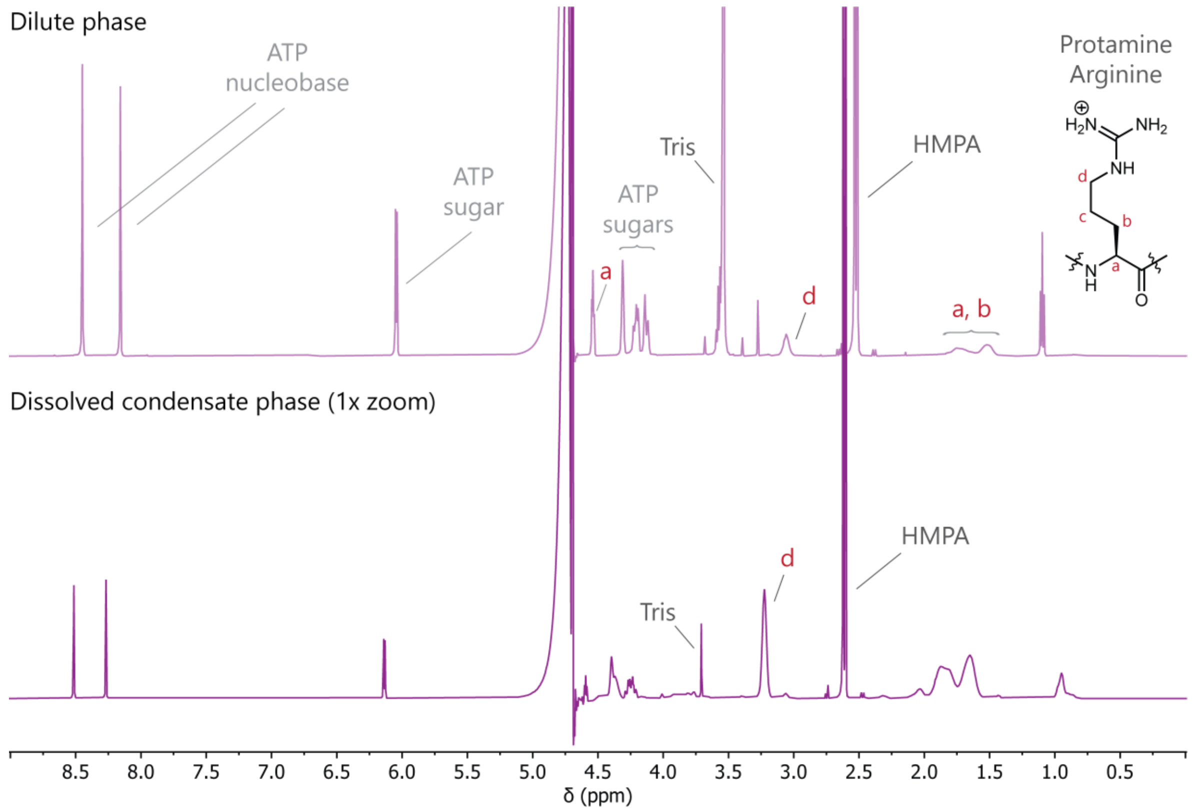

Supporting Figure 40: ^{1}H-NMR-spectra of the dilute phase and dissolved condensate phase for condensates of 1 mM protamine chloride / 25 mM ATP without added salt in 50 mM Tris pH 8.5, used to determine the concentration of protamine and Tris. For the quantification of protamine, the peak of the *d* protons was used.

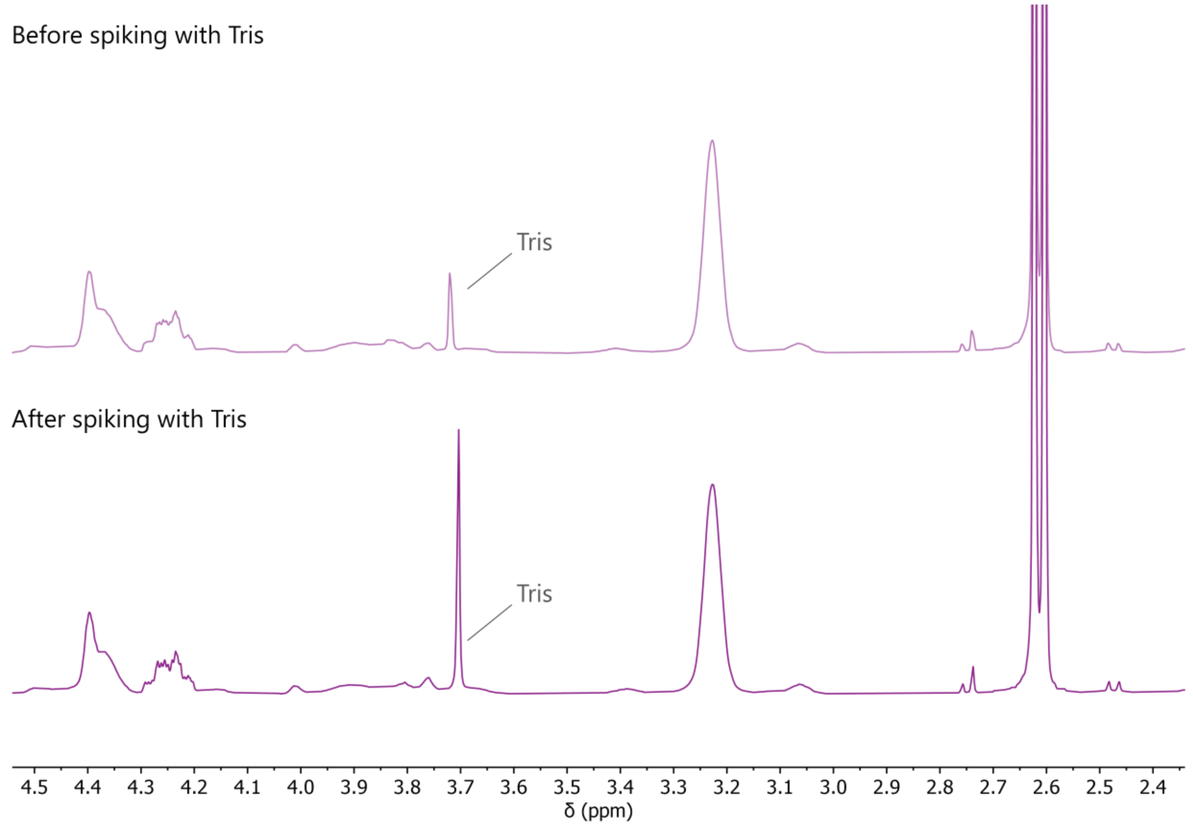

Supporting Figure 41: Spiking with Tris to confirm the chemical shift of its peak in the condensate phase. ^{1}H-NMR-spectra of the dissolved condensate phase of 1 mM protamine chloride / 25 mM ATP condensates prepared with 50 mM LiCl in 50 mM Tris pH 8.5, before and after spiking with an additional 0.5 mM Tris pH 8.5.

3.4.2.2. Na_2HPO_4

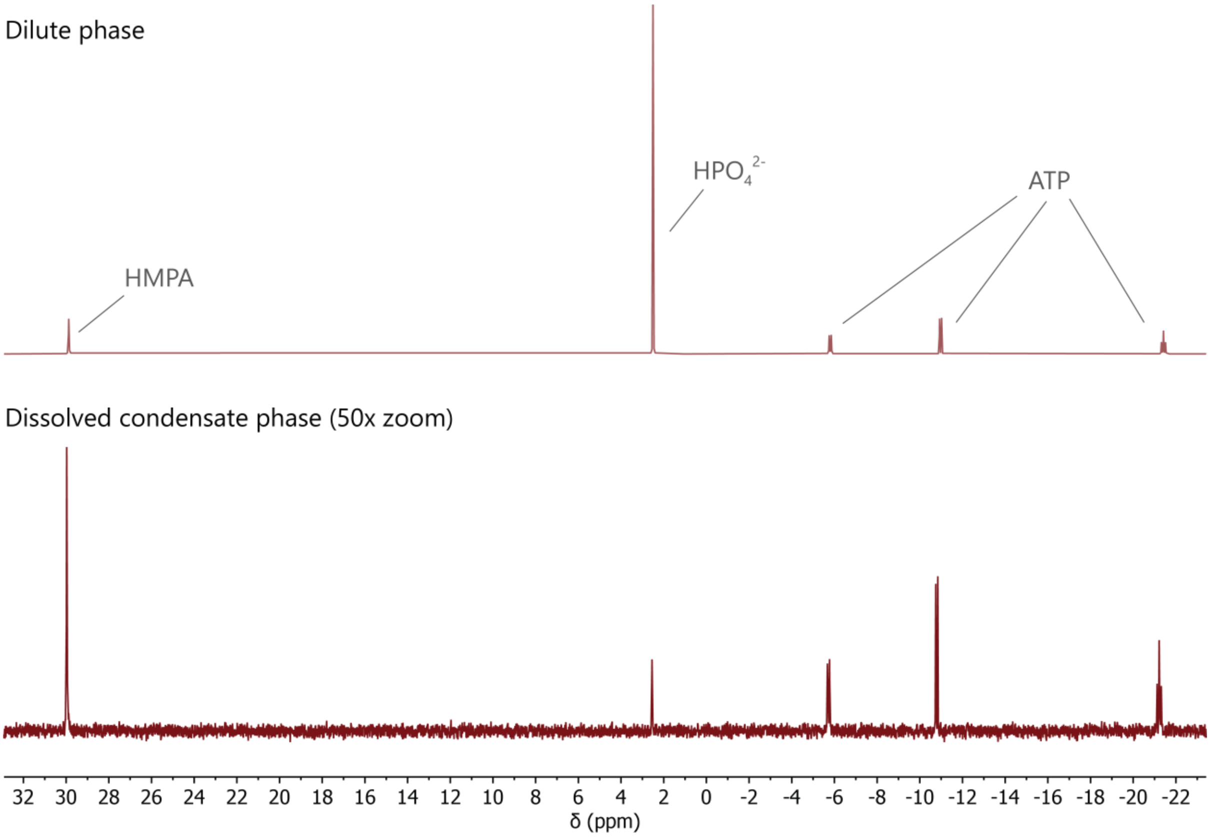

Supporting Figure 42: ^{31}P-NMR-spectra of the dilute phase and dissolved condensate phase for partitioning of 100 mM Na_2HPO_4 (molecule-based, equals 200 mM charge-based, see Supporting Information Section 2.1) in condensates of 1 mM protamine chloride / 25 mM ATP in 50 mM Tris pH 8.5, used to determine the concentration of HPO_4^{2-} and ATP.

Supporting Table 10: Measured charge-based concentrations in the condensate phase and dilute phase and K_P for Na_2HPO_4 partitioning. Charge-based means that the protamine concentration represents the concentration of arginines (21 per protamine), rather than the concentration of peptide. Similarly, for ATP the charge-based concentration should be divided by four to obtain the molecule concentration, while that of HPO_4^{2-} should be divided by 2 to obtain molecule-based concentrations. For monovalent ions the charge-based concentration equals the molecule concentration.

**Compound**	$c_{condensate}$ **(mM)**	c_{dilute} **(mM)**	K_P
Protamine	2200.5 ± 38.6	10.8 ± 0.1	202.9 ± 3.7
ATP	2095.1 ± 113.1	88.1 ± 0.3	23.8 ± 1.3
Tris	59.1 ± 4.1	53.7 ± 0.2	1.10 ± 0.08
Na^+	317.9 ± 21.6	279.6 ± 0.9	1.14 ± 0.08
Cl^-	276.9 ± 12.6	40.7 ± 0.3	6.80 ± 0.31
HPO_4^{2-}	380.1 ± 15.1	211.0 ± 0.6	1.80 ± 0.07

3.4.2.3. NaF

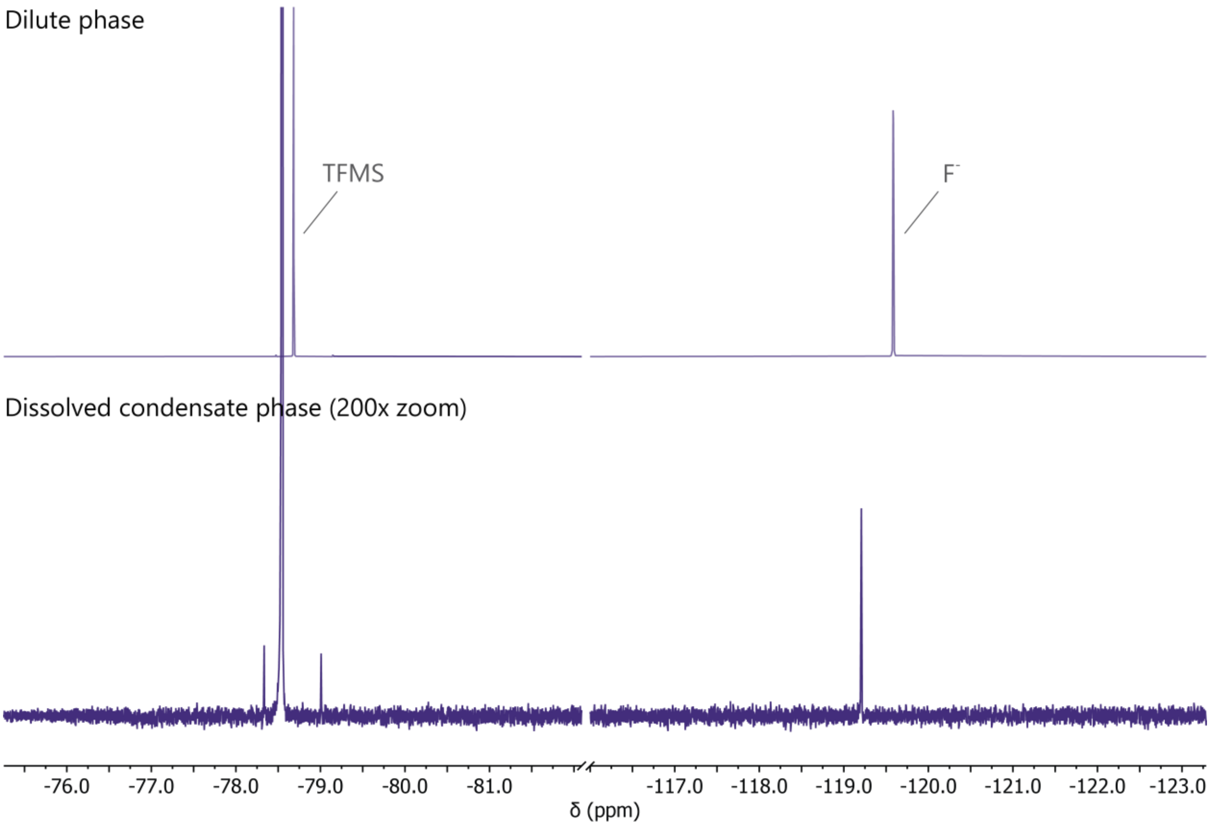

Supporting Figure 43: ^{19}F-NMR-spectra of the dilute phase and dissolved condensate phase for partitioning of 100 mM NaF in condensates of 1 mM protamine chloride / 25 mM ATP in 50 mM Tris pH 8.5, used to determine the concentration of F^- in reference to a sodium trifluoromethanesulfonate (TFMS) internal standard.

Supporting Table 11: Measured charge-based concentrations in the condensate phase and dilute phase and K_P for NaF partitioning. For the Na^+ concentration the added amount of Na^+ from the sodium trifluoromethanesulfonate internal standard was subtracted from the data. Charge-based means that the protamine concentration represents the concentration of arginines (21 per protamine), rather than the concentration of peptide. Similarly, for ATP the charge-based concentration should be divided by four to obtain the molecule concentration. For monovalent ions the charge-based concentration equals the molecule concentration.

**Compound**	$c_{condensate}$ **(mM)**	c_{dilute} **(mM)**	K_P
Protamine	1979.9 ± 79.1	7.41 ± 0.03	267.0 ± 10.7
ATP	2098.0 ± 112.2	82.9 ± 1.1	25.3 ± 1.4
Tris	54.9 ± 3.8	52.2 ± 0.3	1.05 ± 0.07
Na^+	136.9 ± 33.2	184.9 ± 1.7	0.74 ± 0.18
Cl^-	182.7 ± 27.2	41.8 ± 0.4	4.37 ± 0.65
F^-	47.2 ± 1.9	104.9 ± 0.2	0.45 ± 0.02

3.4.2.4. NaBr

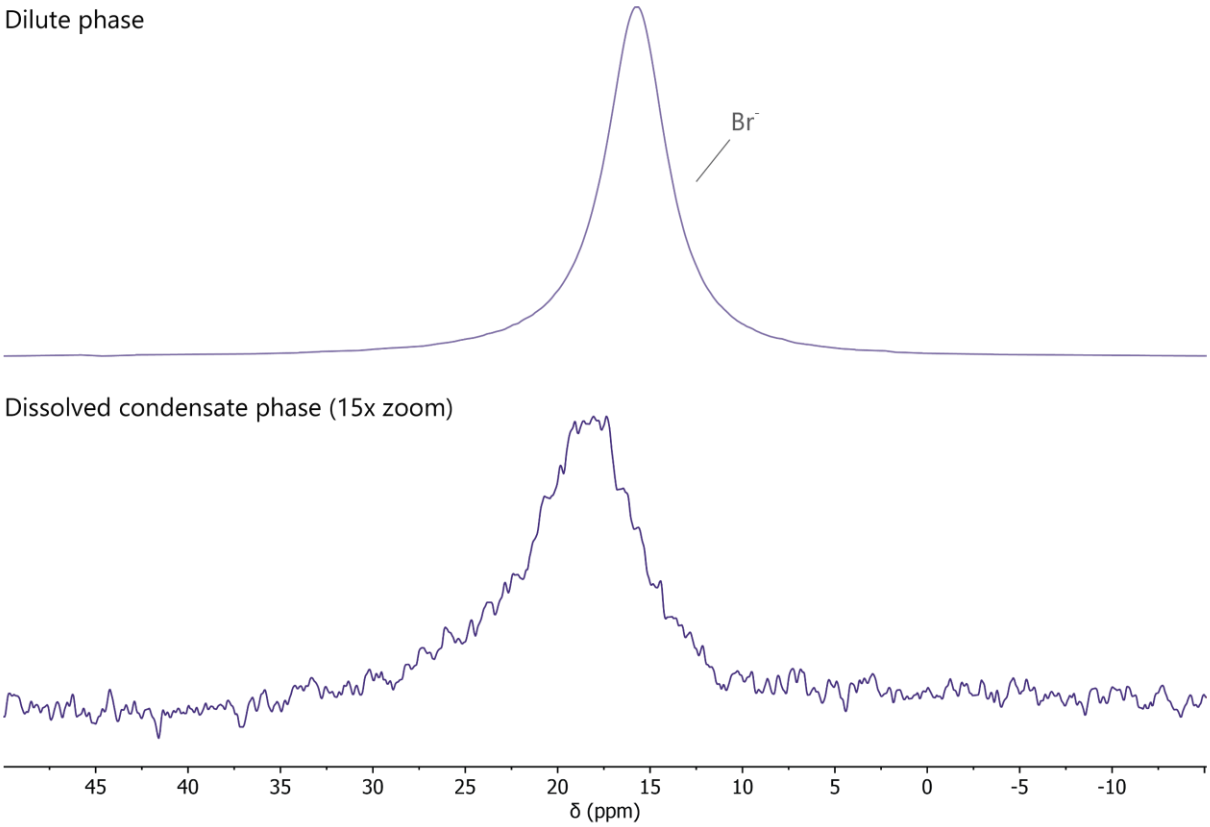

Supporting Figure 44: ^{81}Br-NMR-spectra of the dilute phase and dissolved condensate phase for partitioning of 100 mM NaBr in condensates of 1 mM protamine chloride / 25 mM ATP in 50 mM Tris pH 8.5, used to determine the concentration of Br^-.

Supporting Table 12: Measured charge-based concentrations in the condensate phase and dilute phase and K_P for NaF partitioning. The concentration of Cl^- was not determined because the condensates were dissolved in 1 M KCl. Charge-based means that the protamine concentration represents the concentration of arginines (21 per protamine), rather than the concentration of peptide. Similarly, for ATP the charge-based concentration should be divided by four to obtain the molecule concentration. For monovalent ions the charge-based concentration equals the molecule concentration.

Compound	$c_{condensate}$ (mM)	c_{dilute} (mM)	K_P
Protamine	2135.5 ± 18.4	7.87 ± 0.1	271.4 ± 4.0
ATP	2325.0 ± 60.1	86.1 ± 1.3	27.0 ± 0.8
Tris	55.4 ± 0.07	53.3 ± 0.5	1.04 ± 0.01
Na+	154.2 ± 3.0	186.7 ± 0.7	0.83 ± 0.02
Br^-	125.2 ± 4.6	99.8 ± 0.8	1.26 ± 0.05

3.4.2.5. $NaClO_4$

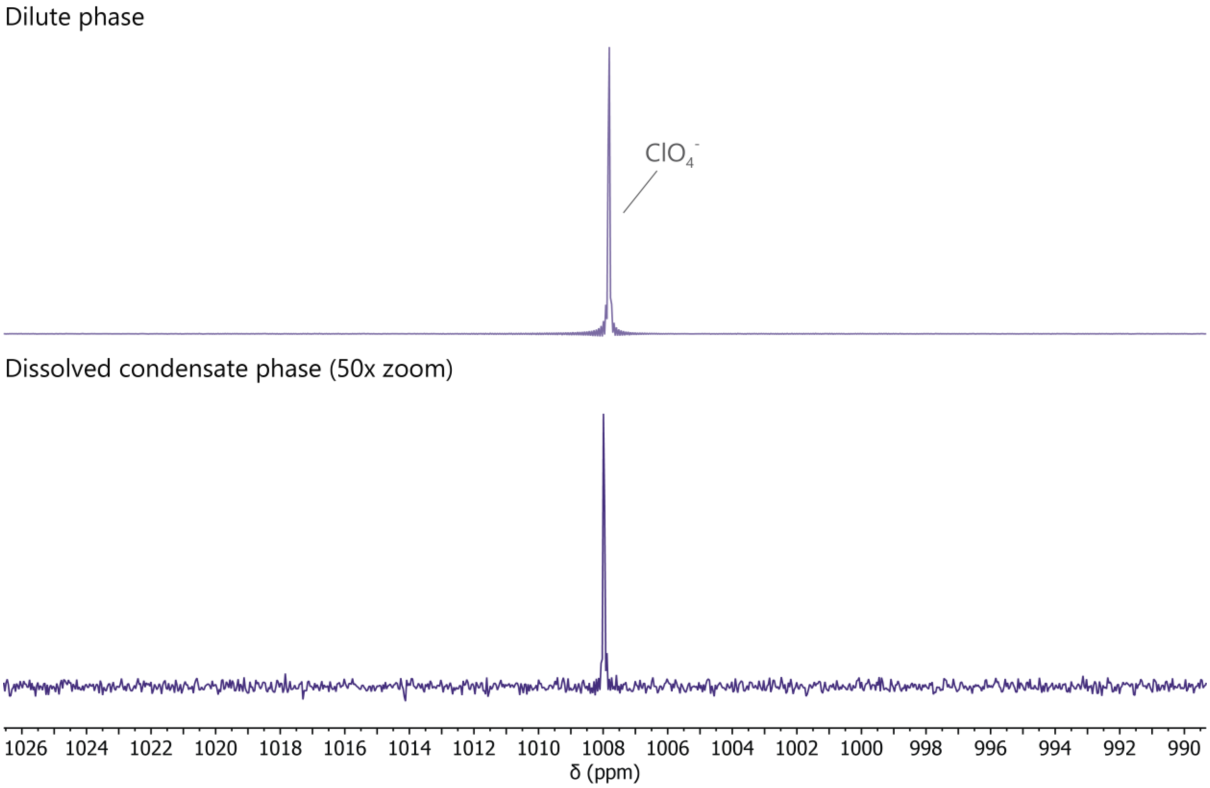

Supporting Figure 45: ^{35}Cl-NMR-spectra of the dilute phase and dissolved condensate phase for partitioning of 100 mM $NaClO_4$ in condensates of 1 mM protamine chloride / 25 mM ATP in 50 mM Tris pH 8.5, used to determine the concentration of ClO_4^-.

Supporting Table 13: Measured charge-based concentrations in the condensate phase and dilute phase and K_P for $NaClO_4$ partitioning. Charge-based means that the protamine concentration represents the concentration of arginines (21 per protamine), rather than the concentration of peptide. Similarly, for ATP the charge-based concentration should be divided by four to obtain the molecule concentration. For monovalent ions the charge-based concentration equals the molecule concentration.

**Compound**	$c_{condensate}$ **(mM)**	c_{dilute} **(mM)**	K_P
Protamine	2134.9 ± 11.2	7.63 ± 0.04	279.7 ± 2.0
ATP	2130.2 ± 18.8	85.5 ± 1.8	24.9 ± 0.6
Tris	53.3 ± 1.8	52.3 ± 0.2	1.02 ± 0.03
Na^+	213.8 ± 1.4	184.5 ± 1.1	1.16 ± 0.01
Cl^-	203.5 ± 4.9	40.6 ± 0.6	5.02 ± 0.14
ClO_4^-	248.6 ± 7.1	96.9 ± 0.2	2.57 ± 0.07

3.4.2.6. $KS^{13}CN$

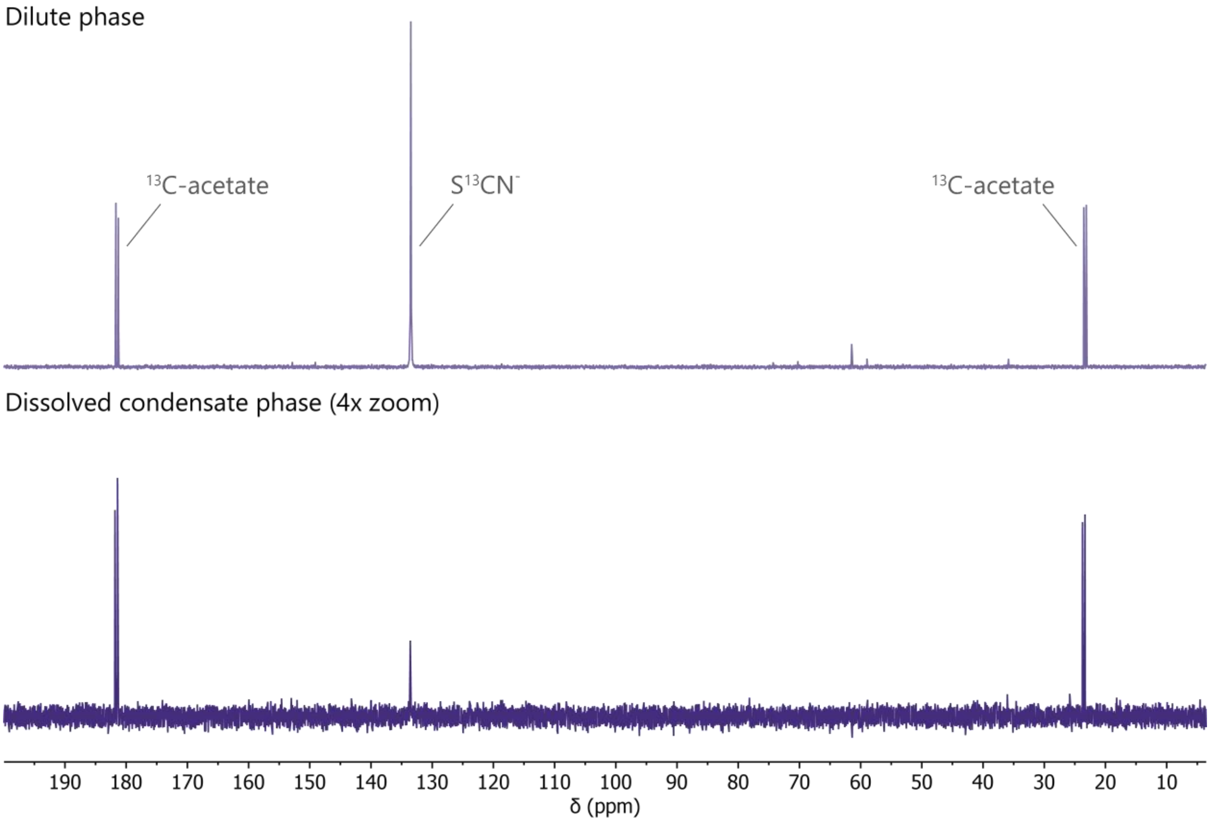

Supporting Figure 46: ^{13}C-NMR-spectra of the dilute phase and dissolved condensate phase for partitioning of 100 mM $KS^{13}CN$ in condensates of 1 mM protamine chloride / 25 mM ATP in 50 mM Tris pH 8.5, used to determine the concentration of $S^{13}CN^-$ in reference to a sodium acetate-1,2-$^{13}C_2$ internal standard.

Supporting Table 14: Measured charge-based concentrations in the condensate phase and dilute phase and K_P for $KS^{13}CN$ partitioning. The concentration of Na^+ was not determined because the condensates were dissolved in 1 M NaBr. Charge-based means that the protamine concentration represents the concentration of arginines (21 per protamine), rather than the concentration of peptide. Similarly, for ATP the charge-based concentration should be divided by four to obtain the molecule concentration. For monovalent ions the charge-based concentration equals the molecule concentration.

Compound	$c_{condensate}$ (mM)	c_{dilute} (mM)	K_P
Protamine	2334.0 ± 24.4	7.71 ± 0.05	302.6 ± 3.8
ATP	2292.0 ± 23.9	84.8 ± 1.3	27.0 ± 0.5
Tris	58.2 ± 0.9	52.3 ± 0.6	1.11 ± 0.02
K^+	79.2 ± 14.3	100.3 ± 0.7	0.79 ± 0.14
Cl^-	54.8 ± 8.3	39.3 ± 0.9	1.39 ± 0.21
$S^{13}CN^-$	245.0 ± 3.6	95.4 ± 2.7	2.57 ± 0.08

3.4.2.7. $MgCl_2$

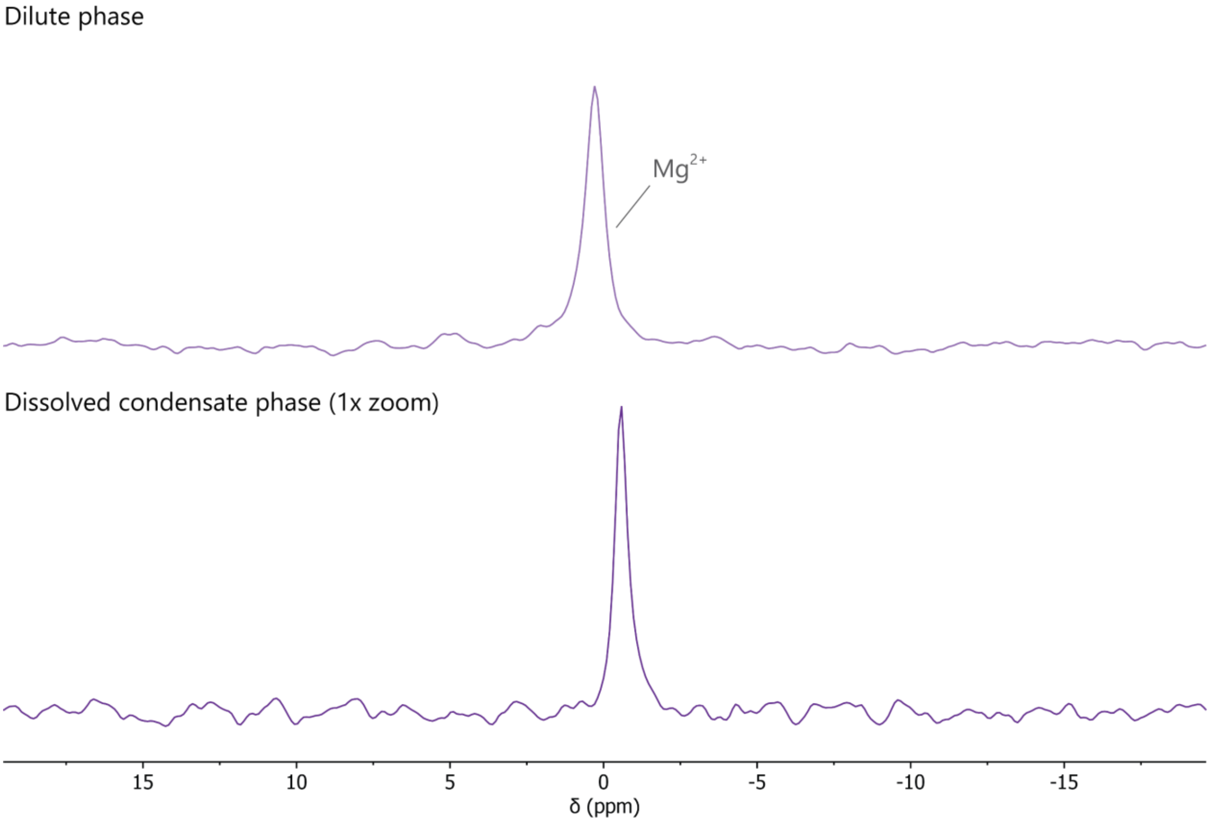

Supporting Figure 47: ^{25}Mg-NMR-spectra of the dilute phase and dissolved condensate phase for partitioning of 50 mM $MgCl_2$ (molecule-based, equals 100 mM charge-based, see Supporting Information Section 2.1) in condensates of 1 mM protamine chloride / 25 mM ATP in 50 mM Tris pH 8.5, used to determine the concentration of Mg^{2+}.

Supporting Table 15: Measured charge-based concentrations in the condensate phase and dilute phase and K_P for $MgCl_2$ partitioning. The concentrations of Mg^{2+} are from a separate sample made with different stock solutions. The protamine, ATP, Tris, Na^+ and Cl^- concentrations were determined for a sample where the condensate phase was dissolved in 1 M KBr; the Mg^{2+} concentration for a sample where the condensate phase was dissolved in 5 M HCl. Charge-based means that the protamine concentration represents the concentration of arginines (21 per protamine), rather than the concentration of peptide. Similarly, for ATP the charge-based concentration should be divided by four to obtain the molecule concentration, while the charge-based concentration of Mg^{2+} should be divided by two to obtain molecule-based concentrations. For monovalent ions the charge-based concentration equals the molecule concentration.

**Compound**	$c_{condensate}$ **(mM)**	c_{dilute} **(mM)**	K_P
Protamine	1553.7 ± 12.8	11.44 ± 0.06	135.8 ± 1.3
ATP	2952.1 ± 31.5	81.2 ± 0.6	36.4 ± 0.5
Tris	47.6 ± 1.04	52.1 ± 0.2	0.91 ± 0.02
Na^+	119.7 ± 1.5	94.1 ± 1.1	1.27 ± 0.02
Cl^-	304.7 ± 11.4	140.7 ±1.3	2.17 ± 0.08
Mg^{2+}	2023.9 ± 57.2	118.0 ± 1.6	17.1 ± 0.5

3.4.2.8. LiCl

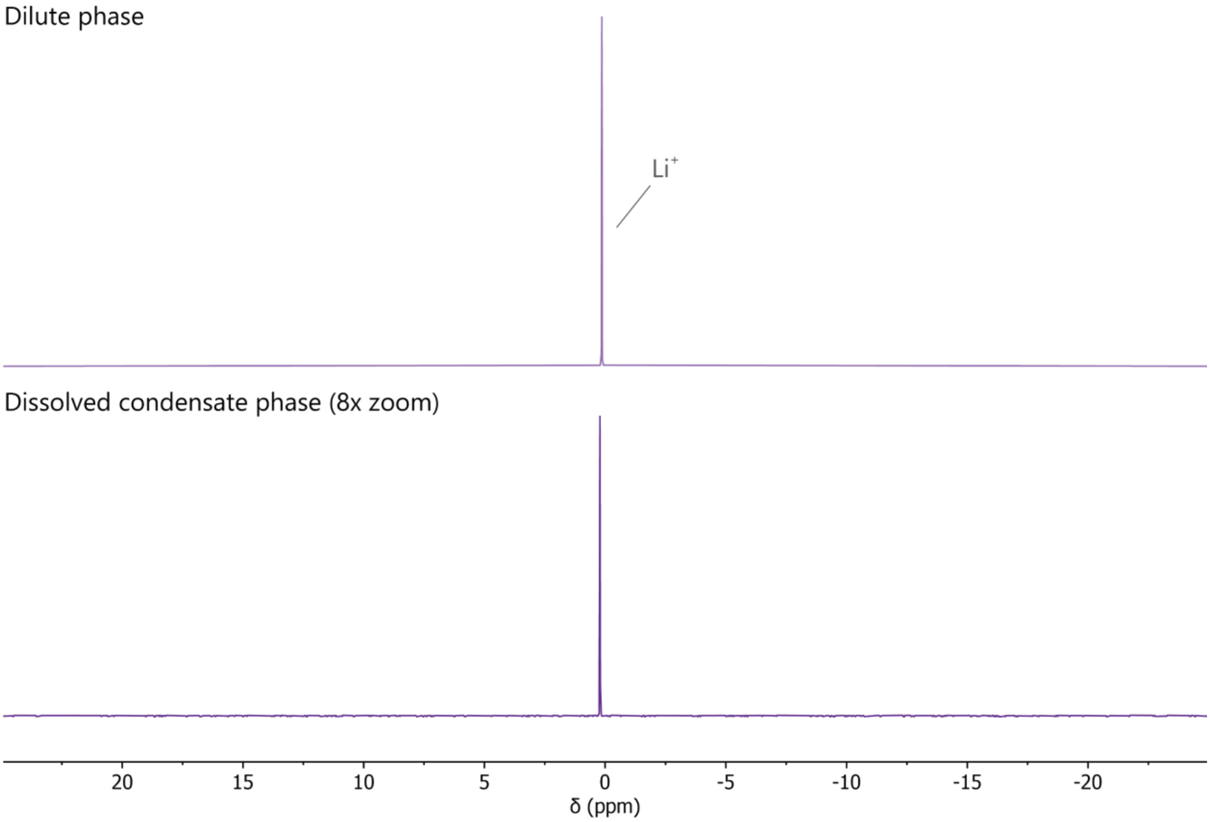

Supporting Figure 48: ^{7}Li-NMR-spectra of the dilute phase and dissolved condensate phase for partitioning of 100 mM LiCl in condensates of 1 mM protamine chloride / 25 mM ATP in 50 mM Tris pH 8.5, used to determine the concentration of Li^+.

Supporting Table 16: Measured charge-based concentrations in the condensate phase and dilute phase and K_P for LiCl partitioning. Charge-based means that the protamine concentration represents the concentration of arginines (21 per protamine), rather than the concentration of peptide. Similarly, for ATP the charge-based concentration should be divided by four to obtain the molecule concentration. For monovalent ions the charge-based concentration equals the molecule concentration.

**Compound**	$c_{condensate}$ **(mM)**	c_{dilute} **(mM)**	K_P
Protamine	2004.4 ± 9.3	8.04 ± 0.02	249.2 ± 1.3
ATP	2239.7 ± 98.7	83.8 ± 1.3	26.7 ± 1.3
Tris	56.5 ± 0.8	52.1 ± 0.2	1.09 ± 0.01
Na^+	149.5 ± 2.4	96.0 ± 1.6	1.56 ± 0.04
Cl^-	299.6 ± 5.8	143.2 ± 0.6	2.09 ± 0.04
Li^+	189.2 ± 2.4	99.8 ± 0.3	1.90 ± 0.02

3.4.2.9. NaCl

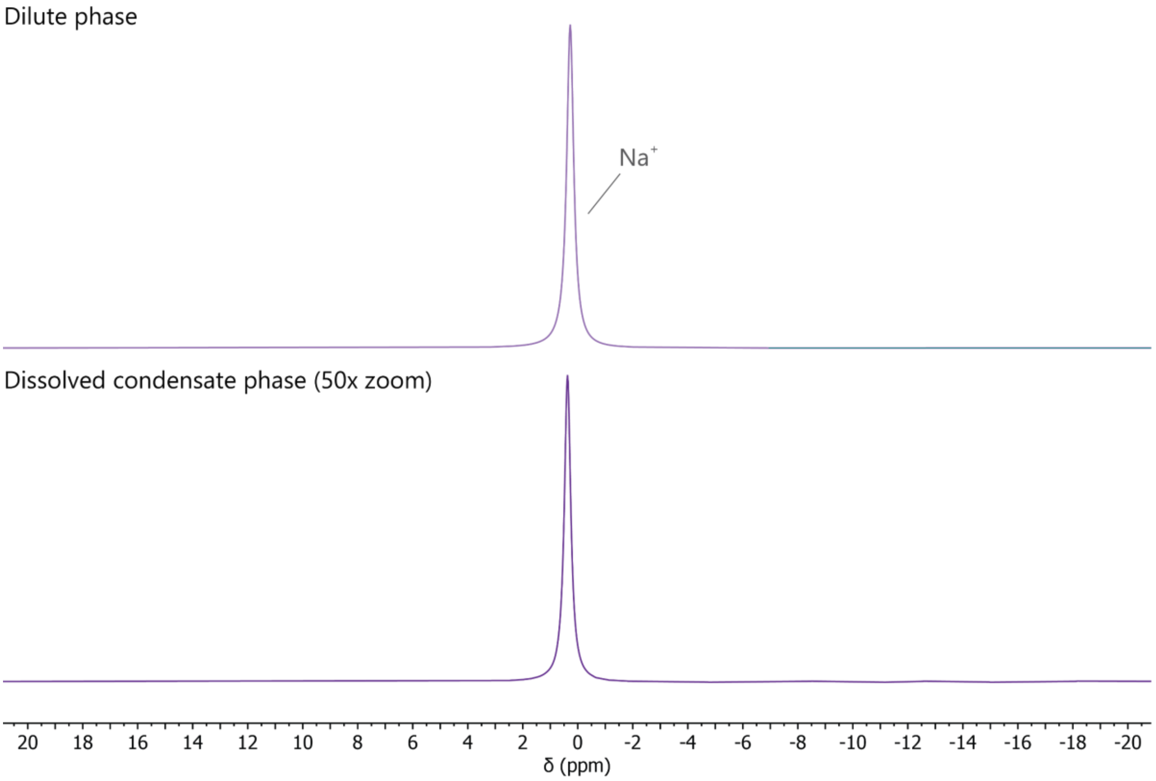

Supporting Figure 49: ^{23}Na-NMR-spectra of the dilute phase and dissolved condensate phase for partitioning of 100 mM NaCl in condensates of 1 mM protamine chloride / 25 mM ATP in 50 mM Tris pH 8.5, used to determine the concentration of Na^+.

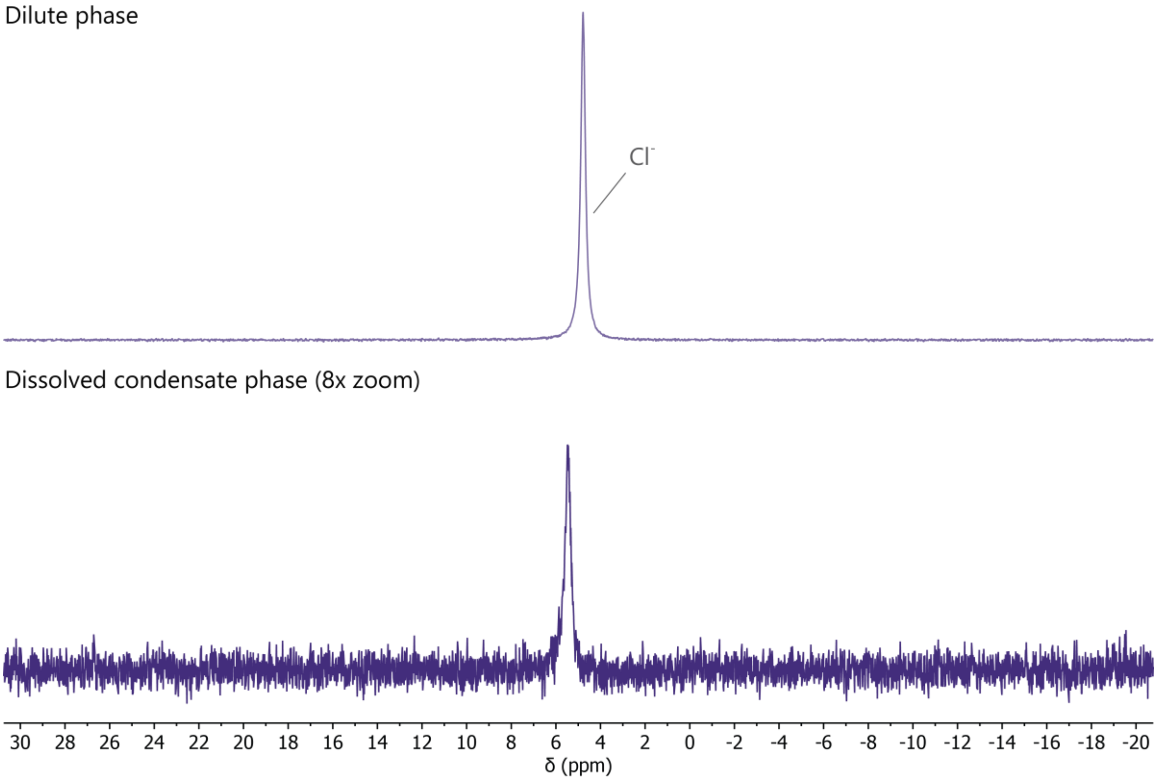

Supporting Figure 50: ^{35}Cl-NMR-spectra of the dilute phase and dissolved condensate phase for partitioning of 100 mM NaCl in condensates of 1 mM protamine chloride / 25 mM ATP in 50 mM Tris pH 8.5, used to determine the concentration of Cl^-.

Supporting Table 17: Measured charge-based concentrations in the condensate phase and dilute phase and K_P for NaCl partitioning for 1st repeat. Charge-based means that the protamine concentration represents the concentration of arginines (21 per protamine), rather than the concentration of peptide. Similarly, for ATP the charge-based concentration should be divided by four to obtain the molecule concentration. For monovalent ions the charge-based concentration equals the molecule concentration.

Compound	$c_{condensate}$ (mM)	c_{dilute} (mM)	K_P
Protamine	1793.8 ± 3.1	7.69 ± 0.06	233.3 ± 2.0
ATP	1908.7 ± 83.6	85.2 ± 1.2	22.4 ± 1.0
Tris	46.7 ± 0.5	52.5 ± 0.2	0.89 ± 0.01
Na^+	194.5 ± 16.9	181.1 ± 2.2	1.07 ± 0.09
Cl^-	292.4 ± 22.0	136.7 ± 4.3	2.14 ± 0.17

Supporting Table 18: Measured charge-based concentrations in the condensate phase and dilute phase and K_P for NaCl partitioning for 2nd repeat (with new protamine & ATP stocks).

Compound	$c_{condensate}$ (mM)	c_{dilute} (mM)	K_P
Protamine	2131.5 ± 18.9	7.78 ± 0.33	273.9 ± 12.0
ATP	2335.8 ± 55.4	84.9 ± 1.1	27.5 ± 0.7
Tris	57.2 ± 2.2	52.6 ± 1.7	1.09 ± 0.05
Na^+	237.0 ± 5.2	198.6 ± 4.3	1.19 ± 0.03
Cl^-	270.2 ± 4.7	141.2 ± 2.4	1.91 ± 0.04

3.4.2.10. KCl

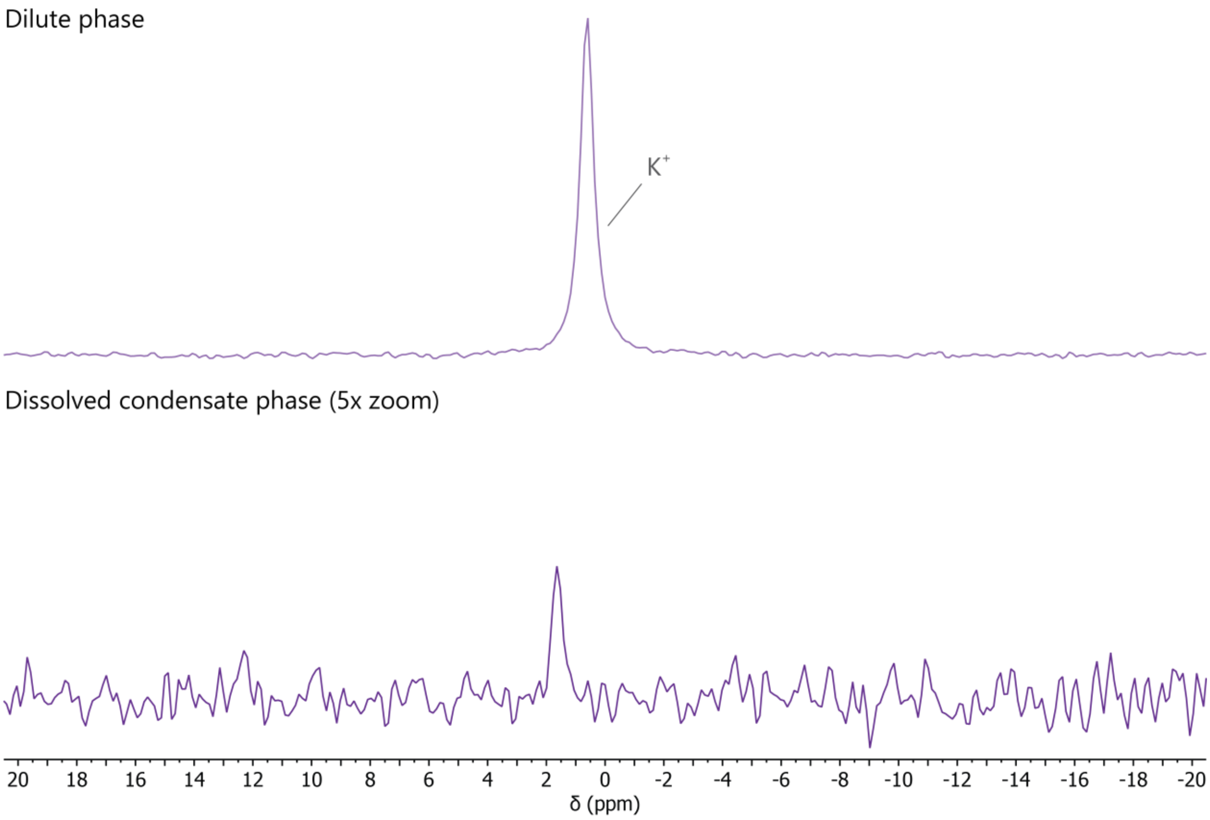

Supporting Figure 51: ^{39}K-NMR-spectra of the dilute phase and dissolved condensate phase for partitioning of 100 mM KCl in condensates of 1 mM protamine chloride / 25 mM ATP in 50 mM Tris pH 8.5, used to determine the concentration of K^+.

Supporting Table 19: Measured charge-based concentrations in the condensate phase and dilute phase and K_P for KCl partitioning. The concentration of Na^+ was not determined because the condensates were dissolved in 1 M NaBr. Charge-based means that the protamine concentration represents the concentration of arginines (21 per protamine), rather than the concentration of peptide. Similarly, for ATP the charge-based concentration should be divided by four to obtain the molecule concentration.

**Compound**	$c_{condensate}$ **(mM)**	c_{dilute} **(mM)**	K_P
Protamine	2184.6 ± 3.3	7.68 ± 0.03	284.3 ± 1.4
ATP	2388.6 ± 61.1	83.9 ± 1.1	28.5 ± 0.8
Tris	61.1 ± 1.8	52.0 ± 0.3	1.17 ± 0.03
K^+	91.7 ± 15.3	100.3 ± 2.9	0.91 ± 0.16
Cl^-	166.3 ± 4.6	139.8 ± 2.7	1.19 ± 0.04

3.4.2.11. CsCl

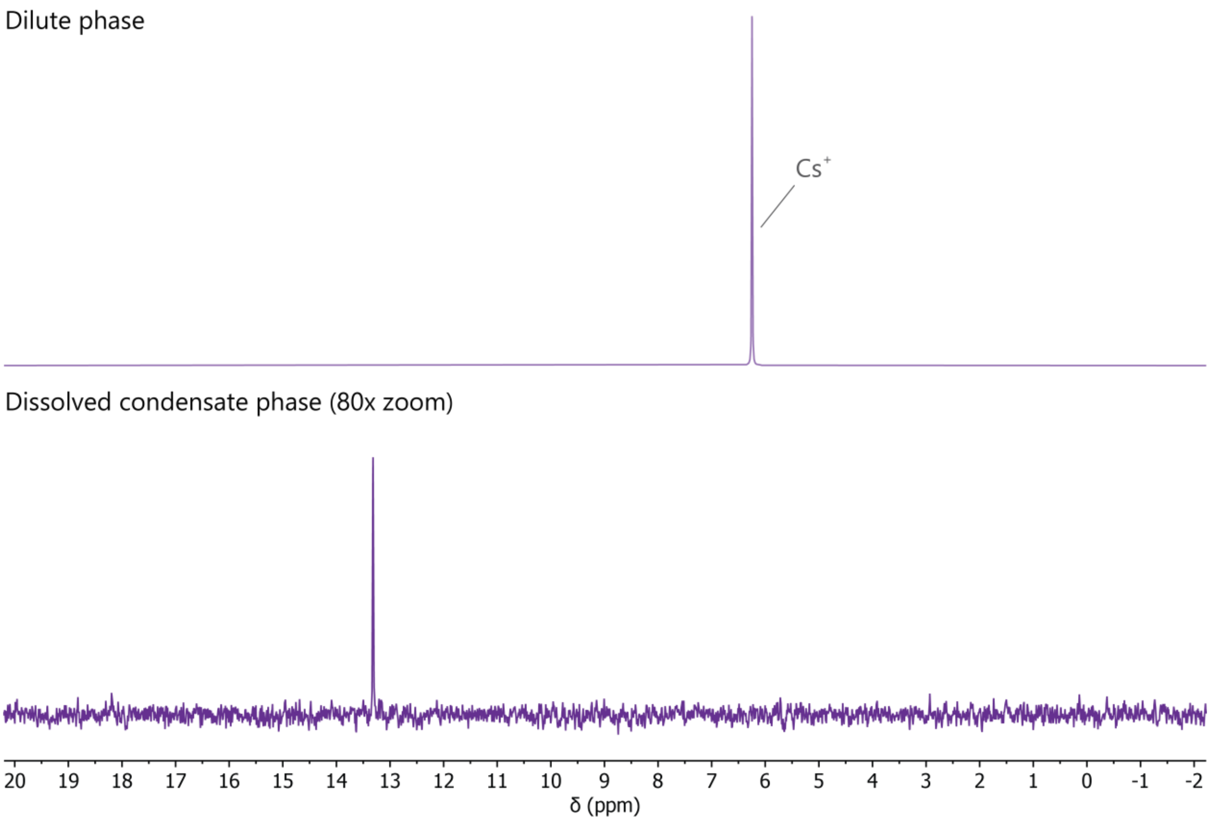

Supporting Figure 52: ^{133}Cs-NMR-spectra of the dilute phase and dissolved condensate phase for partitioning of 100 mM CsCl in condensates of 1 mM protamine chloride / 25 mM ATP in 50 mM Tris pH 8.5, used to determine the concentration of Cs^+.

Supporting Table 20: Measured charge-based concentrations in the condensate phase and dilute phase and K_P for CsCl partitioning. For an explanation of charge-based concentrations, see Supporting Table 17.

**Compound**	$c_{condensate}$ **(mM)**	c_{dilute} **(mM)**	K_P
Protamine	2093.0 ± 3.2	7.34 ± 0.02	284.9 ± 1.0
ATP	2329.8 ± 33.5	78.3 ± 0.4	29.7 ± 0.5
Tris	58.4 ± 1.5	48.8 ± 0.1	1.20 ± 0.03
Na^+	155.2 ± 2.6	92.2 ± 3.6	1.68 ± 0.07
Cl^-	280.0 ± 1.0	140.2 ± 3.2	2.00 ± 0.05
Cs^+	90.8 ± 1.4	103.1 ± 1.8	0.88 ± 0.02

3.4.3. Supporting data ion partitioning

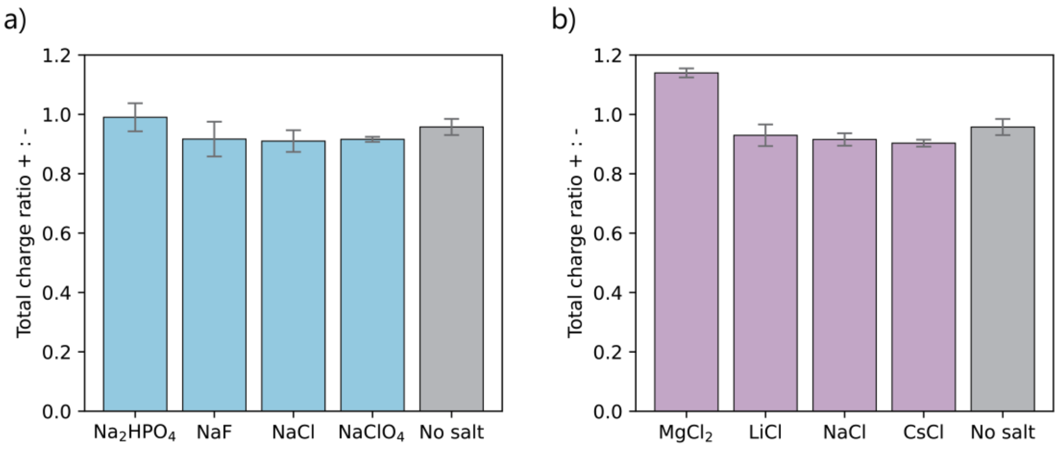

Supporting Figure 53: Total charge ratio of the protamine/ATP condensates $\sum(c_{protamine} + c_{cations} + c_{TrisH^+})/\sum(c_{ATP} + c_{anions})$, charge-based concentrations, showing that only divalent ions give a significant change in the total charge ratio of the condensates. NaBr, $KS^{13}CN$ and KCl were left out of the comparison because these condensates were dissolved in a salt that contained either Na^+ or Cl^-, because of which the total charge balance could not be determined. For $MgCl_2$, the concentration of Mg^{2+} was determined for another sample than the concentration of the other condensate components, which may slightly skew the data.

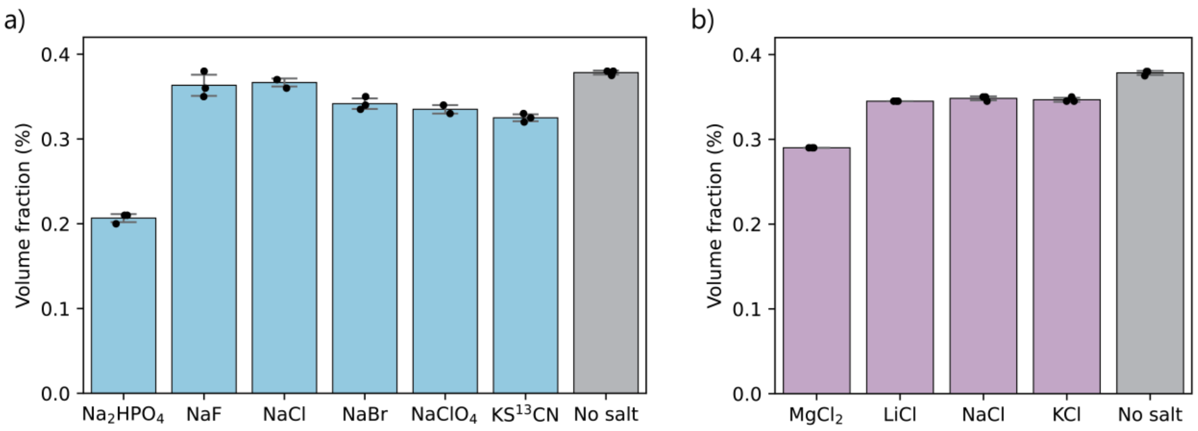

Supporting Figure 54: Volume fraction of protamine/ATP condensates with 100 mM salt (molecule-based, except $MgCl_2$ which is at 50 mM molecule-based, see Supporting Information Section 2.1) as determined by cell counting tubes. **a)** Strong-binding chaotropic anions give a reduction in the volume fraction, as does the divalent phosphate. **b)** Strong-binding cations give a reduction in the volume fraction. CsCl was left out of this comparison because the sample was made with a different protamine and ATP stock.

3.4.4. SCN⁻ partitioning by Raman microscopy

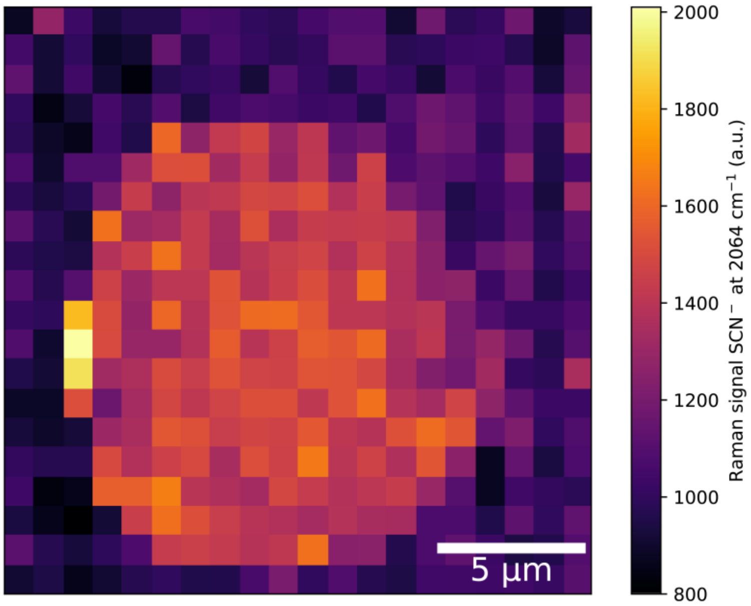

Supporting Figure 55: SCN^- distribution in the protamine/ATP condensate as measured by Raman microscopy, showing that SCN^- is evenly distributed inside the condensate. The peak at $\tilde{\nu}$ = 2064 cm^{-1} was fit with a Gaussian and integrated. Using this procedure, the K_P is determined to be 1.64.

3.5. Supporting data 'Selective ion binding remodels condensate phase diagrams'

3.5.1. Phase diagrams

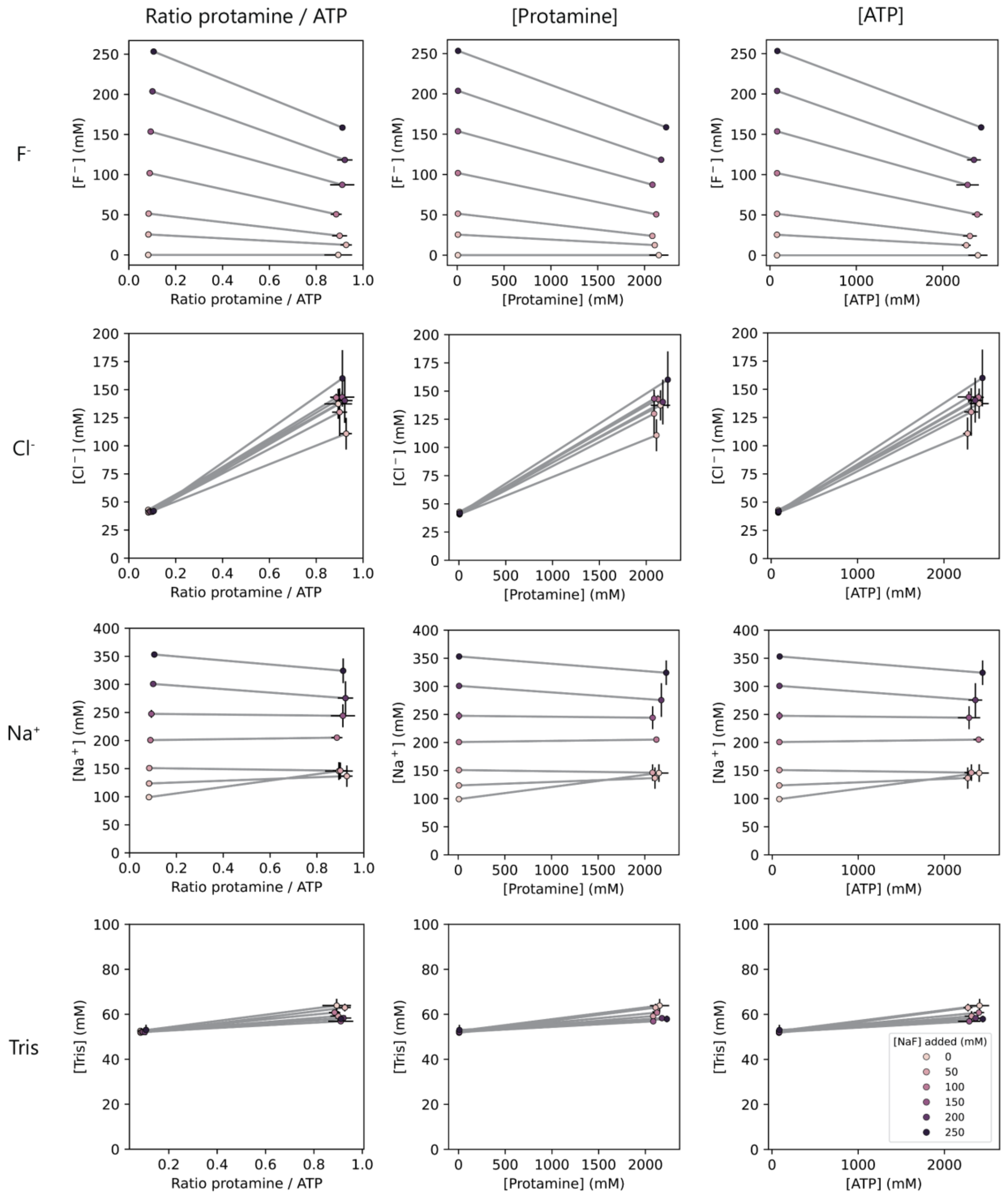

Supporting Figure 56: Phase diagrams for 1 mM protamine / 25 mM ATP condensates with increasing concentrations of NaF. The plots are different representations of the same 6-dimensional dataset. Concentrations represent the charge-based concentrations of each species. Charge-based means that the protamine concentration represents the concentration of arginines (21 per protamine), rather than the concentration of peptide. Similarly, for ATP the charge-based concentration should be divided by four to obtain the molecule concentration. The color of the markers represents the total concentration of NaF that was added to the sample. According to Henderson-Hasselbalch, 30.9% of the Tris is protonated $TrisH^+$ at pH 8.5. Inside the condensate phase this protonation state may be shifted.

Supporting Table 21: Measured charge-based concentrations in the condensate phase for increasing concentrations of NaF. Charge-based means that the protamine concentration represents the concentration of arginines (21 per protamine), rather than the concentration of peptide. Similarly, for ATP the charge-based concentration should be divided by four to obtain the molecule concentration. For monovalent ions the charge-based concentration equals the molecule concentration.

[NaF] added (mM)	$[Protamine]_{cond}$ (mM)	$[ATP]_{cond}$ (mM)	$[Tris]_{cond}$ (mM)	$[Na^+]_{cond}$ (mM)	$[Cl^-]_{cond}$ (mM)	$[F^-]_{cond}$ (mM)
0	2152.0 ± 102.5	2407.3 ± 111.0	63.9 ± 3.0	145.7 ± 15.7	137.4 ± 13.3	0.0 ± 0.0
25	2108.7 ± 27.4	2273.1 ± 52.0	63.0 ± 1.7	136.7 ± 19.0	110.8 ± 14.1	12.4 ± 1.2
50	2083.5 ± 18.1	2315.2 ± 79.9	59.2 ± 2.2	146.3 ± 14.7	129.9 ± 21.1	23.8 ± 0.4
100	2124.7 ± 17.6	2400.2 ± 60.5	60.8 ± 1.3	205.2 ± 2.7	142.9 ± 3.4	50.6 ± 2.7
150	2084.4 ± 6.8	2288.2 ± 129.7	56.9 ± 0.7	244.1 ± 20.4	143.3 ± 4.9	87.3 ± 0.9
200	2177.3 ± 23.2	2361.9 ± 80.2	58.3 ± 0.5	275.5 ± 30.1	140.3 ± 19.8	118.3 ± 1.7
250	2230.1 ± 12.7	2445.7 ± 22.9	57.9 ± 1.5	324.3 ± 21.9	160.1 ± 25.2	158.6 ± 2.2

Supporting Table 22: Measured charge-based concentrations in the dilute phase for increasing concentrations of NaF. Charge-based means that the protamine concentration represents the concentration of arginines (21 per protamine), rather than the concentration of peptide. Similarly, for ATP the charge-based concentration should be divided by four to obtain the molecule concentration. For monovalent ions the charge-based concentration equals the molecule concentration.

[NaF] added (mM)	$[Protamine]_{dil}$ (mM)	$[ATP]_{dil}$ (mM)	$[Tris]_{dil}$ (mM)	$[Na^+]_{dil}$ (mM)	$[Cl^-]_{dil}$ (mM)	$[F^-]_{dil}$ (mM)
0	6.8 ± 0.08	81.8 ± 0.4	52.6 ± 0.4	99.2 ± 0.4	43.0 ± 0.5	0.0 ± 0.0
25	6.8 ± 0.04	81.3 ± 0.6	51.8 ± 0.1	123.5 ± 0.7	40.7 ± 1.3	25.5 ± 0.03
50	6.9 ± 0.02	82.4 ± 1.1	51.9 ± 0.2	150.9 ± 1.9	40.8 ± 0.9	51.4 ± 0.1
100	7.4 ± 0.04	82.6 ± 0.2	52.3 ± 0.2	200.8 ± 1.8	41.6 ± 0.6	101.8 ± 0.2
150	7.8 ± 0.04	83.7 ± 1.1	52.1 ± 0.2	247.5 ± 7.1	41.2 ± 0.4	153.6 ± 0.4
200	8.4 ± 0.07	83.3 ± 0.7	52.1 ± 0.2	300.8 ± 5.2	41.3 ± 1.0	203.7 ± 0.3
250	9.1 ± 0.4	86.2 ± 4.0	53.1 ± 2.3	353.2 ± 0.9	41.9 ± 0.6	253.3 ± 1.0

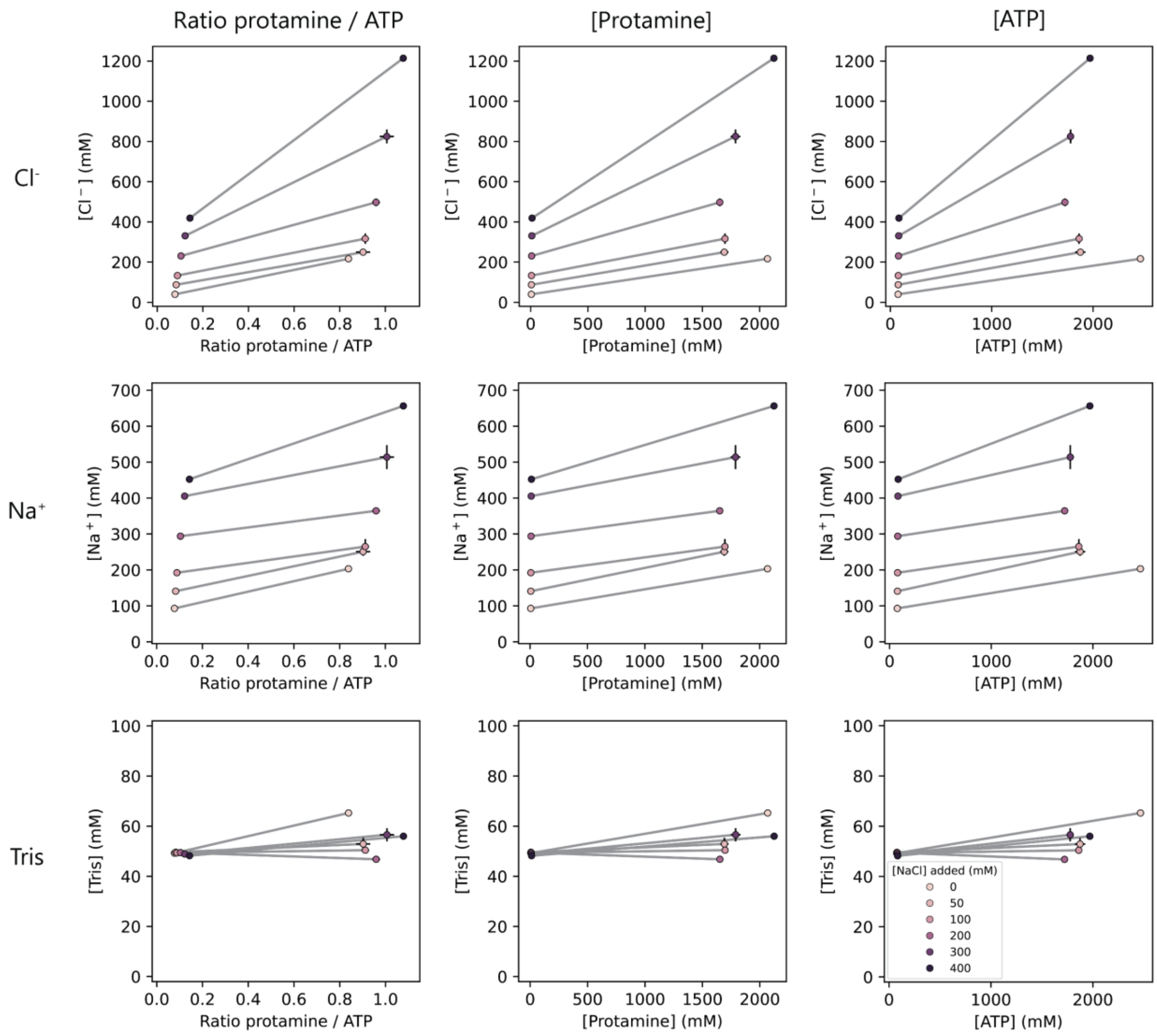

Supporting Figure 57: Phase diagrams for 1 mM protamine / 25 mM ATP condensates with increasing concentrations of NaCl. The plots are different representations of the same 5-dimensional dataset. Concentrations represent the charge-based concentrations of each species. Charge-based means that the protamine concentration represents the concentration of arginines (21 per protamine), rather than the concentration of peptide. Similarly, for ATP the charge-based concentration should be divided by four to obtain the molecule concentration. The color of the markers represents the total concentration of NaCl that was added to the sample. According to Henderson-Hasselbalch, 30.9% of the Tris is protonated $TrisH^+$ at pH 8.5. Inside the condensate phase this protonation state may be shifted.

Supporting Table 23: Measured charge-based concentrations in the condensate phase for increasing concentrations of NaCl. Charge-based means that the protamine concentration represents the concentration of arginines (21 per protamine), rather than the concentration of peptide. Similarly, for ATP the charge-based concentration should be divided by four to obtain the molecule concentration. For monovalent ions the charge-based concentration equals the molecule concentration.

[NaCl] added (mM)	$[Protamine]_{cond}$ (mM)	$[ATP]_{cond}$ (mM)	$[Tris]_{cond}$ (mM)	$[Na^+]_{cond}$ (mM)	$[Cl^-]_{cond}$ (mM)
0	2068.8 ± 9.0	2467.2 ± 35.9	65.3 ± 0.5	203.3 ± 6.5	216.4 ± 10.1
50	1691.9 ± 36.0	1874.6 ± 51.0	52.9 ± 2.4	250.9 ± 11.7	249.4 ± 3.9
100	1697.7 ± 20.8	1861.7 ± 24.1	50.4 ± 1.0	265.0 ± 20.9	316.4 ± 25.6
200	1652.3 ± 6.1	1722.4 ± 30.0	46.8 ± 0.1	364.5 ± 2.1	497.4 ± 19.0
300	1789.8 ± 42.2	1778.0 ± 35.3	56.6 ± 2.6	513.9 ± 33.4	825.1 ± 34.3
400	2125.1	1970.3	56.0	656.3	1214.0

Supporting Table 24: Measured charge-based concentrations in the dilute phase for increasing concentrations of NaCl. Charge-based means that the protamine concentration represents the concentration of arginines (21 per protamine), rather than the concentration of peptide. Similarly, for ATP the charge-based concentration should be divided by four to obtain the molecule concentration. For monovalent ions the charge-based concentration equals the molecule concentration.

[NaCl] added (mM)	$[Protamine]_{dil}$ (mM)	$[ATP]_{dil}$ (mM)	$[Tris]_{dil}$ (mM)	$[Na^+]_{dil}$ (mM)	$[Cl^-]_{dil}$ (mM)
0	5.9 ± 0.05	75.9 ± 1.8	49.2 ± 0.3	93.0 ± 0.8	39.6 ± 0.8
50	6.4 ± 0.03	77.4 ± 1.0	49.6 ± 0.03	140.9 ± 3.5	87.2 ± 1.6
100	7.0 ± 0.05	78.8 ± 0.4	49.3 ± 0.03	192.3 ± 8.0	132.9 ± 4.9
200	8.4 ± 0.1	80.6 ± 0.8	49.5 ± 0.2	293.9 ± 6.1	230.5 ± 8.6
300	10.1 ± 0.04	82.5 ± 0.3	48.9 ± 0.2	405.4 ± 0.9	330.7 ± 0.02
400	12.1 ± 0.05	84.4 ± 0.1	48.2 ± 0.2	452.3 ± 7.5	418.7 ± 1.5

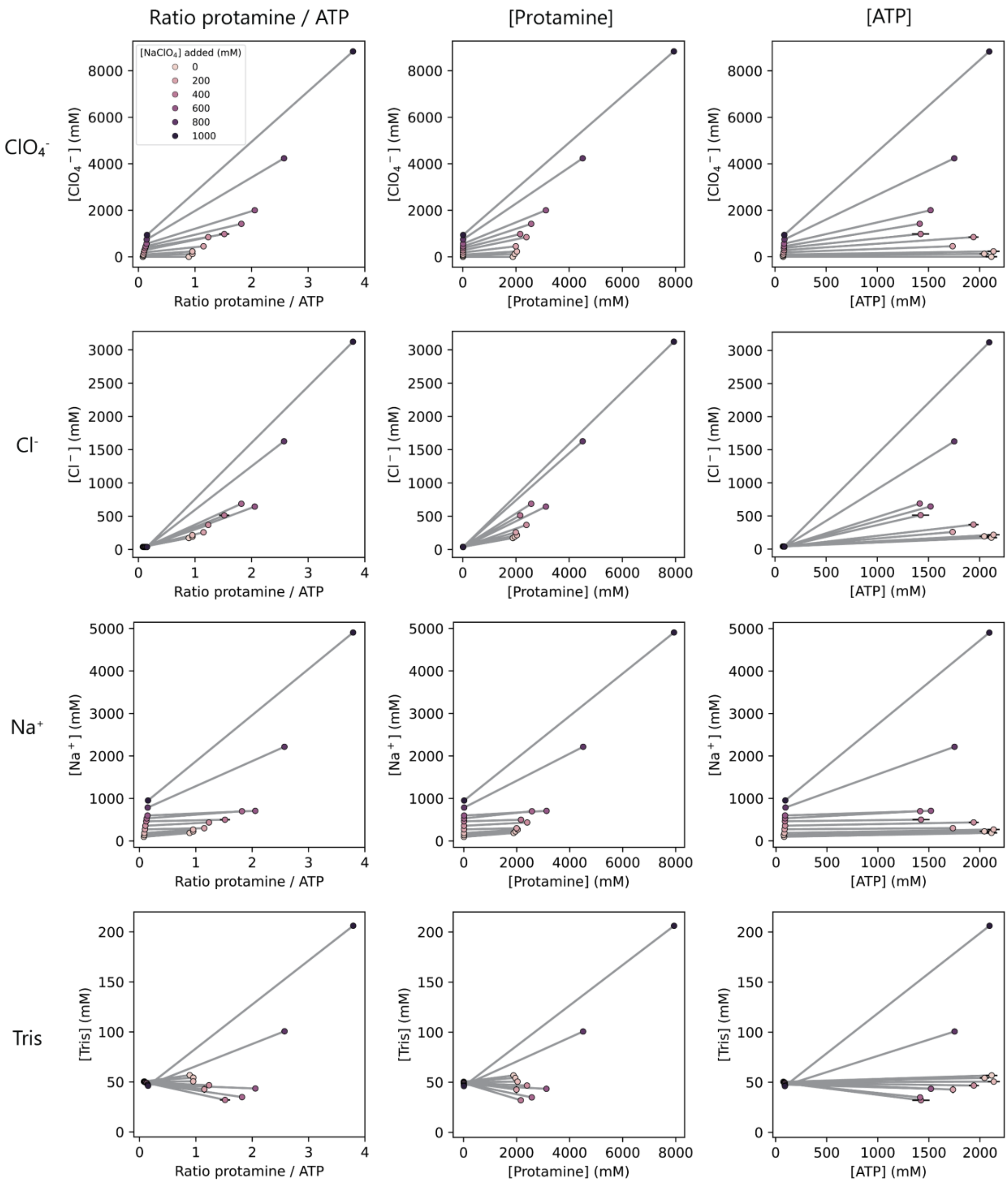

Supporting Figure 58: Phase diagrams for 1 mM protamine / 25 mM ATP condensates with increasing concentrations of $NaClO_4$. The plots are different representations of the same 6-dimensional dataset. Concentrations represent the charge-based concentrations of each species. Charge-based means that the protamine concentration represents the concentration of arginines (21 per protamine), rather than the concentration of peptide. Similarly, for ATP the charge-based concentration should be divided by four to obtain the molecule concentration. The color of the markers represents the total concentration of $NaClO_4$ that was added to the sample. According to Henderson-Hasselbalch, 30.9% of the Tris is protonated $TrisH^+$ at pH 8.5. Inside the condensate phase this protonation state may be shifted.

Supporting Table 25: Measured charge-based concentrations in the condensate phase for increasing concentrations of $NaClO_4$. Charge-based means that the protamine concentration represents the concentration of arginines (21 per protamine), rather than the concentration of peptide. Similarly, for ATP the charge-based concentration should be divided by four to obtain the molecule concentration. For monovalent ions the charge-based concentration equals the molecule concentration.

$[NaClO_4]$ added (mM)	$[Protamine]_{cond}$ (mM)	$[ATP]_{cond}$ (mM)	$[Tris]_{cond}$ (mM)	$[Na^+]_{cond}$ (mM)	$[Cl^-]_{cond}$ (mM)	$[ClO_4^-]_{cond}$ (mM)
0	1883.1 ± 3.7	2114.7 ± 56.2	56.8 ± 0.5	188.2 ± 3.1	172.3 ± 12.2	0.0 ± 0.0
50	1952.1 ± 35.0	2044.6 ± 45.1	54.3 ± 0.9	224.5 ± 6.8	192.5 ± 18.7	124.1 ± 0.3
100	2034.9 ± 16.6	2135.0 ± 59.4	50.6 ± 1.7	267.2 ± 7.1	215.8 ± 9.6	232.6 ± 2.9
200	1995.4 ± 4.3	1735.8 ± 17.9	42.7 ± 3.6	300.1 ± 18.4	258.3 ± 1.7	453.3 ± 14.2
300	2392.7 ± 9.4	1939.9 ± 49.3	46.7 ± 1.7	435.3 ± 14.1	369.2 ± 6.3	846.2 ± 20.4
400	2159.3 ± 14.4	1422.9 ± 84.0	32.0 ± 1.2	500.0 ± 22.3	512.6 ± 11.9	980.1 ± 3.1
500	2572.2 ± 27.7	1414.1 ± 16.6	34.9 ± 0.1	699.7 ± 15.6	687.2 ± 38.9	1418.6 ± 9.5
600	3124.8	1520.3	43.4	708.2	644.3	2001.9
800	4507.5	1751.7	100.7	2215.9	1626.6	4236.3
1000	7937.5	2093.3	206.2	4904.5	3122.9	8831.0

Supporting Table 26: Measured charge-based concentrations in the dilute phase for increasing concentrations of $NaClO_4$. Charge-based means that the protamine concentration represents the concentration of arginines (21 per protamine), rather than the concentration of peptide. Similarly, for ATP the charge-based concentration should be divided by four to obtain the molecule concentration. For monovalent ions the charge-based concentration equals the molecule concentration.

**$[NaClO_4]$ added (mM)**	**$[Protamine]_{dil}$ (mM)**	**$[ATP]_{dil}$ (mM)**	**$[Tris]_{dil}$ (mM)**	**$[Na^+]_{dil}$ (mM)**	**$[Cl^-]_{dil}$ (mM)**	**$[ClO_4^-]_{dil}$ (mM)**
0	6.2 ± 0.02	79.7 ± 0.9	50.3 ± 0.03	96.6 ± 4.6	39.2 ± 0.5	0.0 ± 0.0
50	6.6 ± 0.09	76.2 ± 0.1	50.5 ± 0.3	137.3 ± 2.9	39.5 ± 0.2	48.9 ± 1.2
100	7.1 ± 0.03	82.3 ± 0.6	50.1 ± 0.4	186.2 ± 2.5	39.3 ± 0.2	95.7 ± 0.4
200	8.2 ± 0.02	83.9 ± 0.9	49.9 ± 0.01	272.8 ± 7.3	37.8 ± 1.3	188.5 ± 5.5
300	9.3 ± 0.00	88.9 ± 1.7	49.5 ± 0.07	357.6 ± 8.2	37.3 ± 1.4	281.5 ± 7.1
400	10.4 ± 0.06	86.9 ± 0.2	49.2 ± 0.07	461.7 ± 16.2	38.5 ± 1.0	375.4 ± 3.3
500	11.4 ± 0.01	88.7 ± 2.4	48.9 ± 0.4	530.6 ± 24.6	37.5 ± 2.1	451.1 ± 17.1
600	12.7 ± 0.03	88.9 ± 2.0	47.7 ± 0.3	598.1 ± 32.2	36.8 ± 1.1	561.8 ± 12.9
800	13.5 ± 0.16	92.7 ± 4.2	46.9 ± 0.2	785.7 ± 34.2	36.2 ± 0.9	746.7 ± 24.4
1000	13.7 ± 0.16	90.9 ± 1.5	46.2 ± 0.06	952.6 ± 10.2	37.1 ± 0.4	939.1 ± 0.5

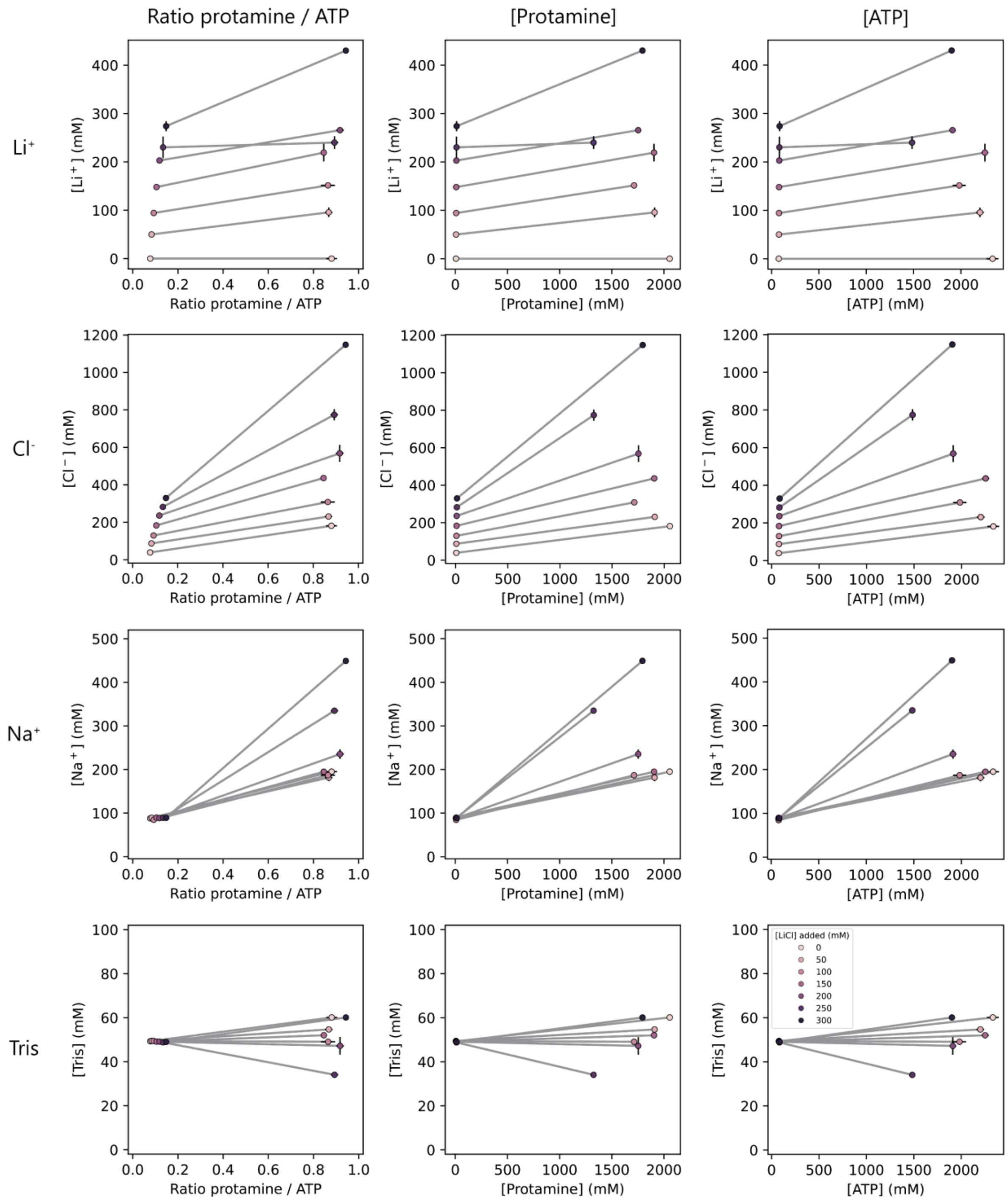

Supporting Figure 59: Phase diagrams for 1 mM protamine / 25 mM ATP condensates with increasing concentrations of LiCl. The plots are different representations of the same 6-dimensional dataset. Concentrations represent the charge-based concentrations of each species. Charge-based means that the protamine concentration represents the concentration of arginines (21 per protamine), rather than the concentration of peptide. Similarly, for ATP the charge-based concentration should be divided by four to obtain the molecule concentration. The color of the markers represents the total concentration of LiCl that was added to the sample. According to Henderson-Hasselbalch, 30.9% of the Tris is protonated $TrisH^+$ at pH 8.5. Inside the condensate phase this protonation state may be shifted.

Supporting Table 27: Measured charge-based concentrations in the condensate phase for increasing concentrations of LiCl. For an explanation of charge-based concentrations see tables above.

[LiCl] added (mM)	$[Protamine]_{cond}$ (mM)	$[ATP]_{cond}$ (mM)	$[Tris]_{cond}$ (mM)	$[Na^+]_{cond}$ (mM)	$[Cl^-]_{cond}$ (mM)	$[Li^+]_{cond}$ (mM)
0	2054.9 ± 10.8	2333.9 ± 62.3	60.1 ± 0.1	195.0 ± 7.4	181.4 ± 6.9	0.0 ± 0.0
50	1910.3 ± 9.9	2201.6 ± 40.5	54.7 ± 1.0	181.4 ± 8.3	230.9 ± 8.9	95.7 ± 10.1
100	1713.6 ± 15.8	1982.4 ± 69.0	49.1 ± 0.6	186.9 ± 7.2	308.4 ± 2.0	151.4 ± 1.3
150	1904.9 ± 4.1	2252.8 ± 37.0	52.0 ± 1.3	194.9 ± 3.2	436.3 ± 12.7	219.2 ± 18.2
200	1753.6 ± 0.4	1911.5 ± 34.8	47.2 ± 4.0	235.3 ± 10.8	568.2 ± 45.1	265.6 ± 0.2
250	1325.4 ± 5.3	1484.2 ± 26.8	34.1 ± 0.3	334.9 ± 3.0	773.9 ± 29.9	239.9 ± 13.2
300	1794.4	1902.1	60.1	449.1	1147.8	430.1

Supporting Table 28: Measured charge-based concentrations in the dilute phase for increasing concentrations of LiCl. For an explanation of charge-based concentrations see tables above.

[LiCl] added (mM)	$[Protamine]_{dil}$ (mM)	$[ATP]_{dil}$ (mM)	$[Tris]_{dil}$ (mM)	$[Na^+]_{dil}$ (mM)	$[Cl^-]_{dil}$ (mM)	$[Li^+]_{dil}$ (mM)
0	5.9 ± 0.02	76.4 ± 0.2	49.3 ± 0.1	87.9 ± 2.8	39.1 ± 1.4	0.0 ± 0.0
50	6.5 ± 0.04	78.1 ± 0.2	49.5 ± 0.1	89.1 ± 0.5	87.4 ± 0.8	49.9 ± 2.4
100	7.3 ± 0.05	78.9 ± 0.1	49.4 ± 0.2	84.5 ± 1.1	129.7 ± 0.7	94.3 ± 0.2
150	8.3 ± 0.03	79.1 ± 0.2	49.1 ± 0.2	89.6 ± 0.6	183.2 ± 1.6	147.9 ± 0.8
200	9.7 ± 0.14	82.1 ± 0.6	49.1 ± 0.4	88.5 ± 1.0	236.0 ± 0.8	202.8 ± 0.7
250	11.1 ± 0.03	82.8 ± 0.2	48.8 ± 0.1	89.0 ± 1.2	282.1 ± 2.2	230.1 ± 22.3
300	12.6 ± 0.05	85.3 ± 0.7	49.0 ± 0.3	89.0 ± 1.8	329.5 ± 0.2	273.9 ± 10.5

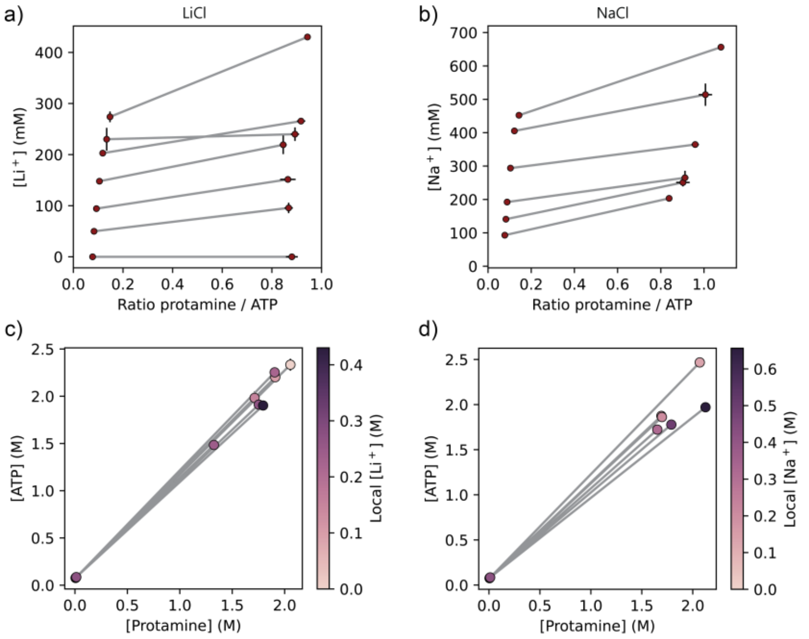

Supporting Figure 60: Phase diagrams for 1 mM protamine / 25 mM ATP condensates with increasing concentrations of LiCl (a, c) or NaCl (b, d). **a)** Li^+ is localized to the condensates and the partitioning stays approximately equal for increasing concentrations of Li^+. **b)** Na^+ is localized to the condensates and the partitioning stays approximately equal for increasing concentrations of Na^+. **c)** Addition of LiCl does not change the ratio of protamine : ATP inside the condensates. **d)** Addition of NaCl mildly changes the ratio of protamine : ATP, shifting the ratio towards more protamine at high NaCl concentrations.

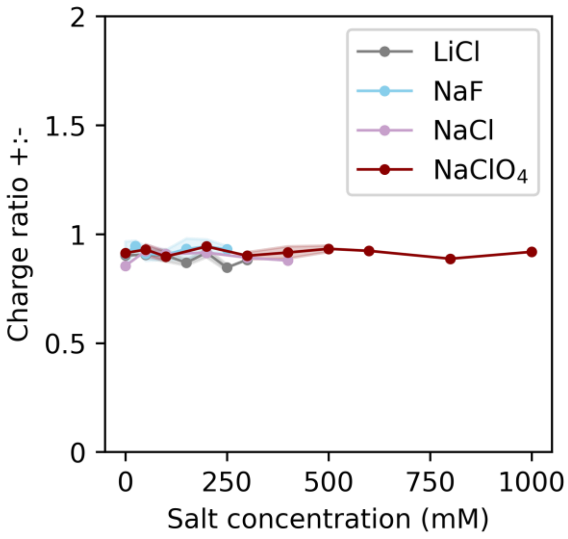

Supporting Figure 61: The overall charge ratio of the protamine/ATP condensates stays equal for different concentrations of LiCl, NaF, NaCl and $NaClO_4$.

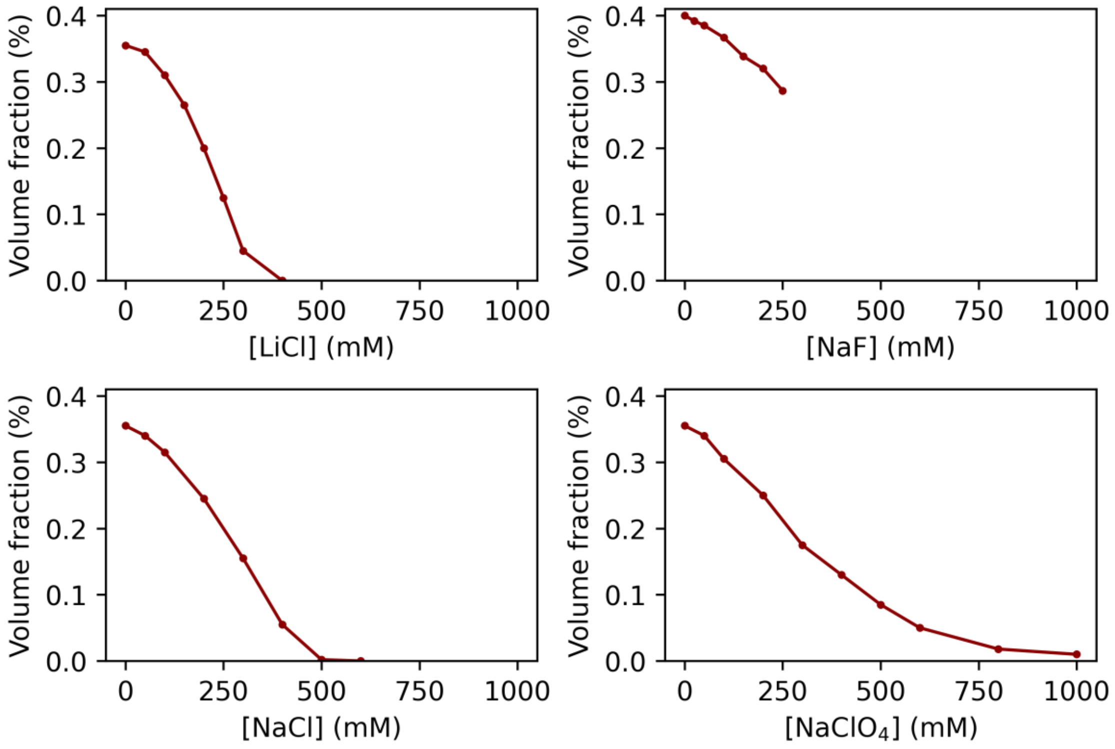

Supporting Figure 62: Changes in condensate volume fraction as a function of salt concentration for LiCl, NaF, NaCl and $NaClO_4$. Condensates dissolve more readily for LiCl and NaCl than for $NaClO_4$.

3.5.2. $[ATP]_{crit}$ protamine/ATP

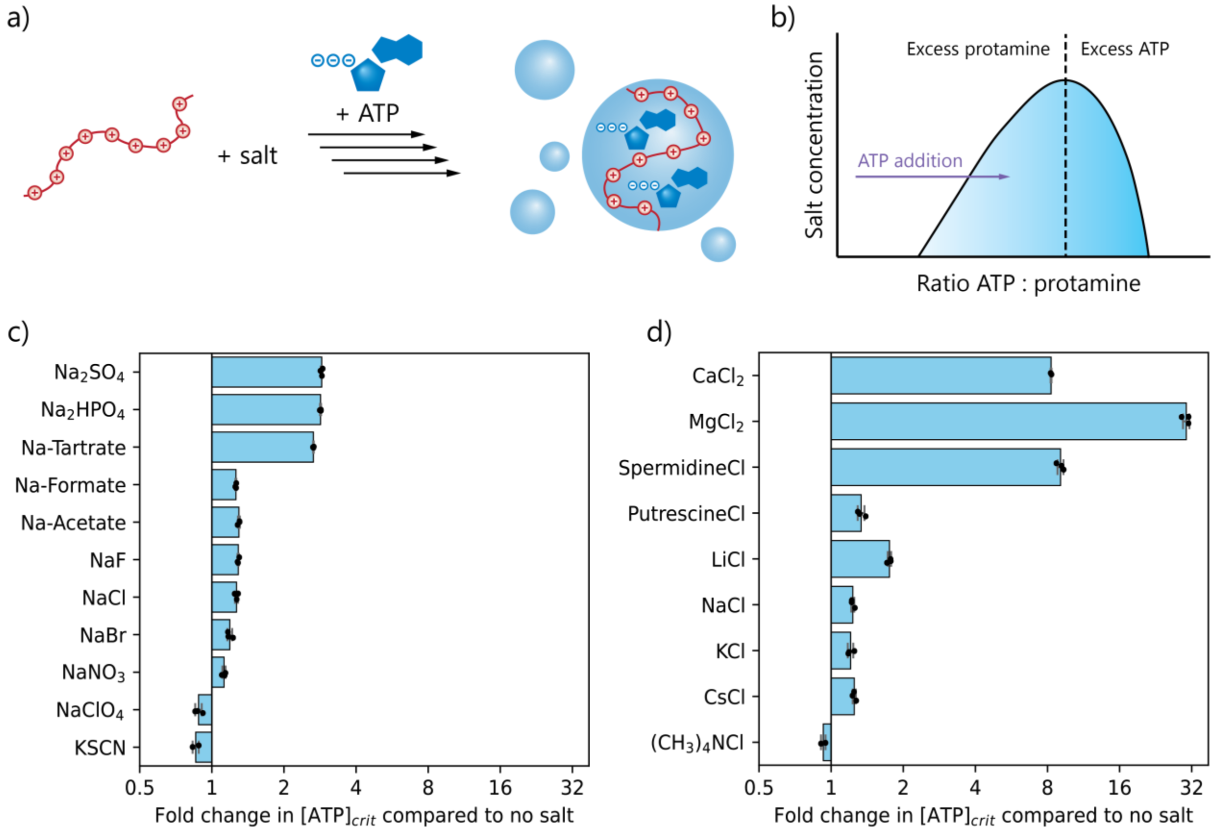

Supporting Figure 63: Fold-change in critical concentration of ATP ($[ATP]_{crit}$) required for formation of protamine/ATP coacervates. **a)** 0.5 mM protamine was mixed with 100 mM of monovalent salt, 50 mM of divalent salt, or 40 mM spermidine (molecule-based, see Supporting Information Section 2.1) and ATP was titrated in. The onset of turbidity is reported as $[ATP]_{crit}$. **b)** Phase diagram illustrating the approach of the binodal upon ATP titration. **c)** Anions that bind to protamine lower the $[ATP]_{crit}$, because they shield the positive charge on protamine and thereby lower the excess of positive charge. **d)** Cations that bind to ATP significantly increase the $[ATP]_{crit}$, because they shield the negative charge on ATP and thereby increase the excess of positive charge. Ions that do not bind specifically increase the $[ATP]_{crit}$ because of aspecific charge-screening effects. Interestingly, tetramethylammonia, which is highly chaotropic and thus binds ATP weakly lowers the $[ATP]_{crit}$, most likely due to its high concentration it outcompetes the stronger binding of ATP's counterion sodium. It can also clearly be seen that the effect of multivalent ions is significantly stronger than that of monovalent ions, and all multivalent ions increase the $[ATP]_{crit}$, even phosphate which does bind to protamine.

3.5.3. $[Polyion]_{crit}$ of other heterotypic condensates

To assess whether our observations for the effect of ions on protamine/ATP condensate stability are general, we determined the $[polyion]_{crit}$ for different types of heterotypic condensates (protamine/polyA, protamine/D_{30} and K_{30}/D_{30}), by titrating in either the polycation or the polyanion (Supporting Figures 64 & 65). We observe that for many of these condensate systems, addition of 100 mM of any salt always lowers the $[polyion]_{crit}$, indicating that for these condensates made with longer polymers, a small amount of salt helps to lower charge-repulsion within polymer chains and favors phase separation. Still, we observe a similar trend between different ions as for protamine/ATP: salt ions that bind specifically to the polyion in excess lower the critical concentration of the oppositely charged polyion, while ions that bind to the polyion that is titrated in increase the $[polyion]_{crit}$. These observations show that the effect of specific ion binding on condensate stability is a general effect for different heterotypic condensates.

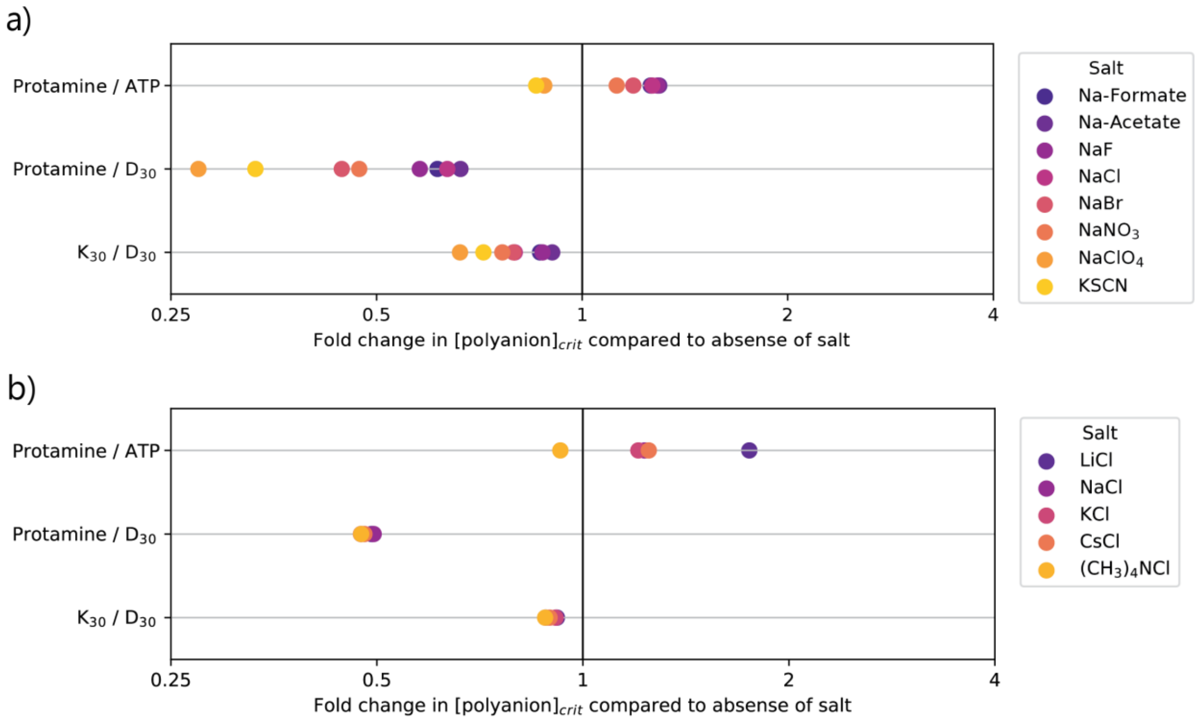

Supporting Figure 64: Fold-change in critical polyanion concentration ($[polyanion]_{crit}$) for formation of different charge-based condensates for 100 mM of different monovalent salts, with respect to the critical concentration in absence of salt. All systems were studied at pH 8.5. Anions that bind to the polycation have a stronger tendency to lower the $[polyanion]_{crit}$, while cations that bind to the polyanion have a stronger tendency to increase the $[polyanion]_{crit}$.

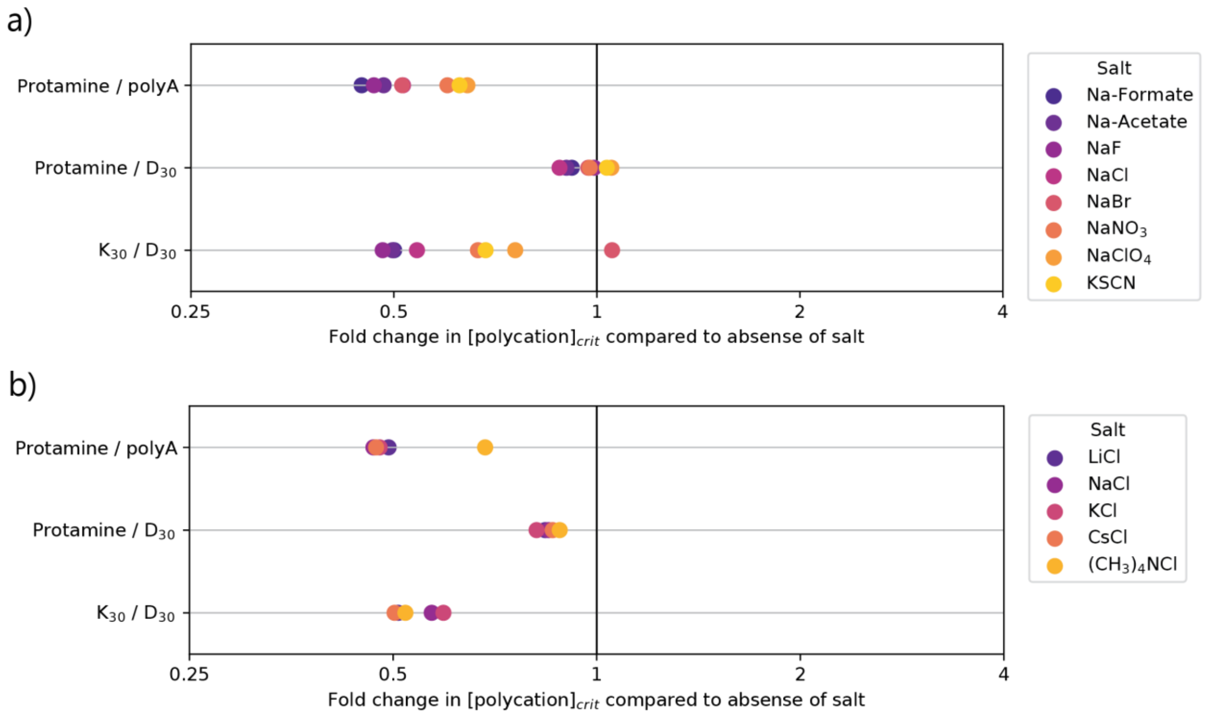

Supporting Figure 65: Fold-change in critical polycation concentration ($[polycation]_{crit}$) for formation of different charge-based condensates for 100 mM of different monovalent salts, with respect to the critical concentration in absence of salt. All systems were studied at pH 8.5. Anions that bind to the polycation have a stronger tendency to increase the $[polycation]_{crit}$, while cations that bind to the polyanion have a stronger tendency to lower the $[polycation]_{crit}$.

3.5.4. Salt effects on a homotypic hydrophobic condensate

To understand the effect of different ions on homotypic, hydrophobic condensates, we investigated condensates formed by the hydrophobic peptide derivative LLssLL[1] and observe that in this case, both specific ion binding and salting out effects play a role (Supporting Figure 66). The peptide derivative has two free N-termini which are positively charged at pH 8.5, and can therefore bind chaotropic anions. We observe a reduction in LCST in presence of perchlorate and thiocyanate, in line with binding to and subsequent neutralization of the N-termini resulting in a larger hydrophobicity and increased phase separation propensity. However, we also observe a reduction in LCST for phosphate and fluoride, indicating that also general salting-out effects play a role.

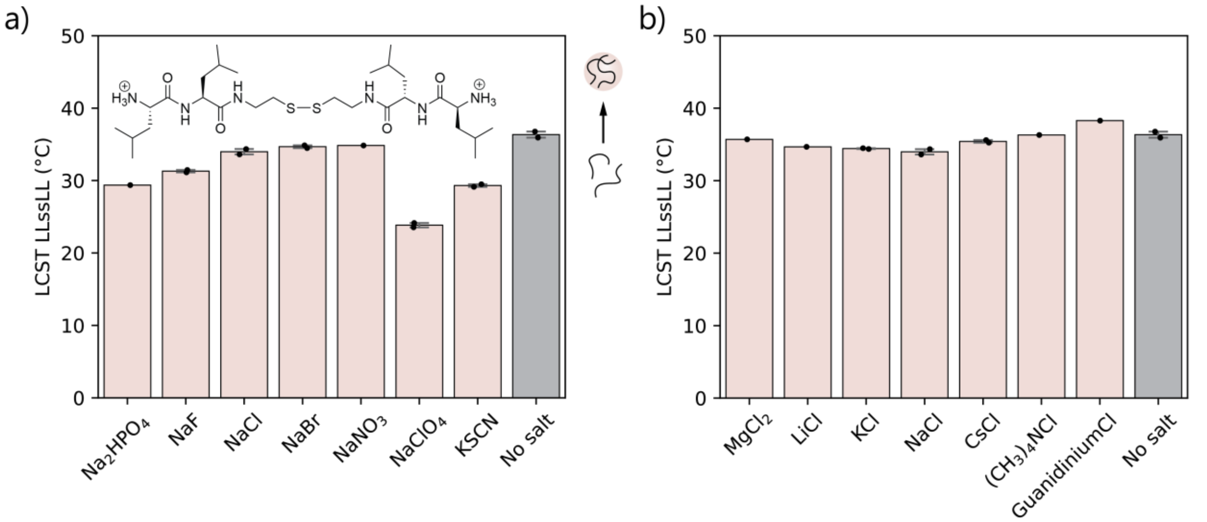

Supporting Figure 66: Lower critical solution temperature (LCST) of hydrophobic condensates of 3 mg/mL peptide derivative LLssLL with 100 mM of monovalent salt or 50 mM of divalent $MgCl_2$ or Na_2HPO_4 (molecule-based, see Supporting Information Section 2.1; to obtain equal charge concentrations of all salts) in 50 mM Tris pH 8.5. **a)** The LCST is lowered, i.e., condensates are stabilized, for both chaotropic and kosmotropic anions, via neutralization of the N-termini by selective chaotrope binding and the salting-out effect, respectively. **b)** Cations have a less pronounced effect on the LCST of LLssLL. Chaotropic cations slightly disfavor phase separation because they solubilize the hydrophobic peptide.

3.6. Supporting data 'Ion binding changes viscosity and interface potential of condensates'

Supporting Table 29: Diffusion coefficients and viscosities for protamine/ATP condensates prepared with different salts (molecule-based concentrations, see Supporting Information Section 2.1), as measured by RICS.

**Sample**	***D*** **(µm²/s)**	**η_d (mPa·s)**
No salt	0.144 ± 0.006	1.49 ± 0.06
50 mM $MgCl_2$	0.124 ± 0.010	1.74 ± 0.13
100 mM LiCl	0.173 ± 0.017	1.25 ± 0.12
100 mM NaCl	0.171 ± 0.014	1.26 ± 0.11
100 mM CsCl	0.182 ± 0.011	1.19 ± 0.07
50 mM Na_2HPO_4	0.177 ± 0.009	1.22 ± 0.06
100 mM NaF	0.153 ± 0.015	1.41 ± 0.14
100 mM $NaClO_4$	0.158 ± 0.009	1.37 ± 0.08
200 mM LiCl	0.253 ± 0.054	0.88 ± 0.18
200 mM NaCl	0.232 ± 0.024	0.93 ± 0.09
200 mM CsCl	0.254 ± 0.022	0.85 ± 0.07
200 mM NaF	0.168 ± 0.024	1.30 ± 0.21
200 mM $NaClO_4$	0.101 ± 0.008	2.13 ± 0.17

Supporting Table 30: Parameters used to calculate ζ-potentials of pLys/pGlu condensates. c_{ion} is the ionic strength of the buffer (determined via Henderson-Hasselbalch), the added salt and the counterions of the protamine and ATP. η_d was determined by RICS. E_0 was determined by testing the lowest electric field strength at which droplets moved. Other parameters used to calculate the ζ-potential are T = 298 K and water viscosity of 0.891 mPa·s. Salt concentrations are molecule-based, see Supporting Information Section 2.1.

**Sample**	**c_{ion} (mM)**	**η_d (mPa·s)**	***E (V)***	**E_0 (V)**	**ζ (mV)**
No salt	102.3	1.49 ± 0.06	8	1.9	-4.34 ± 1.71
50 mM $MgCl_2$	252.8	1.74 ± 0.13	5	2.5	3.49 ± 2.61
100 mM LiCl	202.6	1.25 ± 0.12	5 & 12	2.5	-0.40 ± 0.21
100 mM NaCl	202.6	1.26 ± 0.11	5 & 8	2.5	-1.07 ± 0.73
100 mM CsCl	203.3	1.19 ± 0.07	5 & 8	2.5	-0.81 ± 0.54
50 mM Na_2HPO_4	249.1	1.22 ± 0.06	5	1.5	-1.91 ± 0.57
100 mM NaF	203.2	1.41 ± 0.14	5 & 8	2.5	-2.17 ± 0.96
100 mM $NaClO_4$	202.3	1.37 ± 0.08	5 & 12	2.5	-1.19 ± 0.31
200 mM LiCl	302.9	0.88 ± 0.18	5	2.5	0.96 ± 0.50
200 mM NaCl	302.8	0.93 ± 0.09	5	2.5	-0.68 ± 0.24
200 mM CsCl	304.2	0.85 ± 0.07	5 & 8	2.5	-0.31 ± 0.08
200 mM NaF	304.1	1.30 ± 0.21	5 & 8	2.5	-1.46 ± 0.11
200 mM $NaClO_4$	302.3	2.13 ± 0.17	5 & 8	2.5	0.02 ± 0.11

3.7. Supporting data 'Ion binding alters RNA duplex stability in condensates'

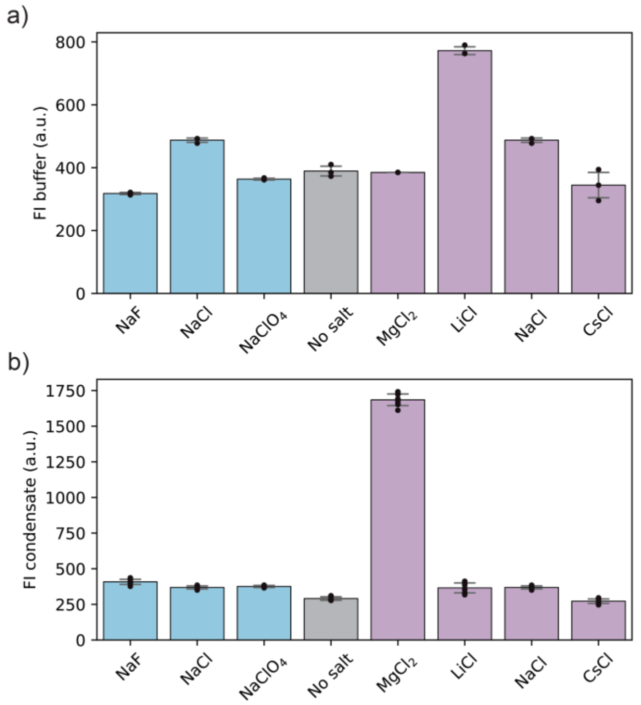

Supporting Figure 67: Cy3-Fluorescence intensity (FI) of the RNA FRET pair in buffer and in protamine/ATP condensates. **a)** In buffer all fluorescence intensities are in the same order of magnitude. **b)** Inside the condensate, however, the fluorescence intensity is significantly higher in presence of $MgCl_2$, indicating that the presence of $MgCl_2$ enhances the partitioning of RNA into the condensates.

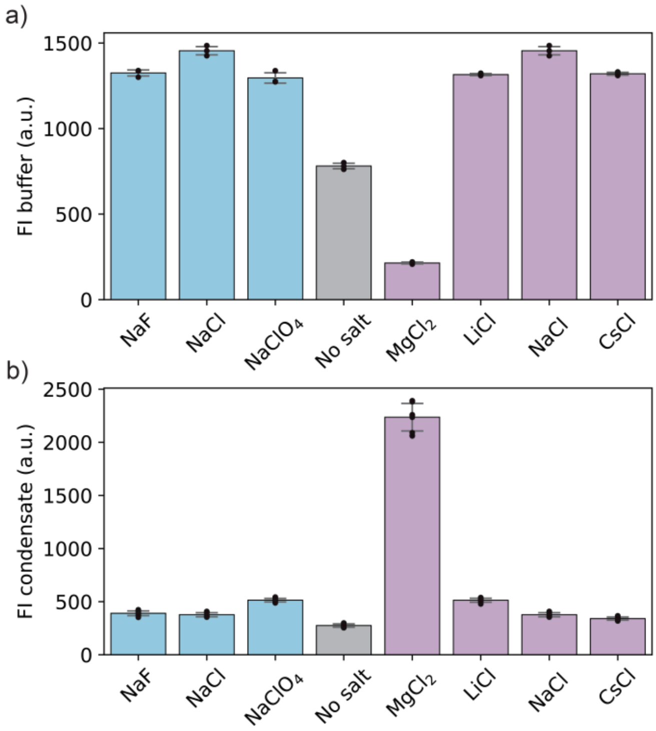

Supporting Figure 68: Cy3-Fluorescence intensity (FI) of the DNA FRET pair in buffer and in protamine/ATP condensates. **a)** In buffer all fluorescence intensities are in the same order of magnitude, except for $MgCl_2$, which seems to lower the fluorescence intensity. **b)** Inside the condensate, however, the fluorescence intensity is significantly higher in presence of $MgCl_2$, indicating that the presence of $MgCl_2$ enhances the partitioning of DNA into the condensates. Either quenching by Mg^{2+} is less pronounced in the condensates, or the increased partitioning of DNA in presence of $MgCl_2$ is still an underestimation.

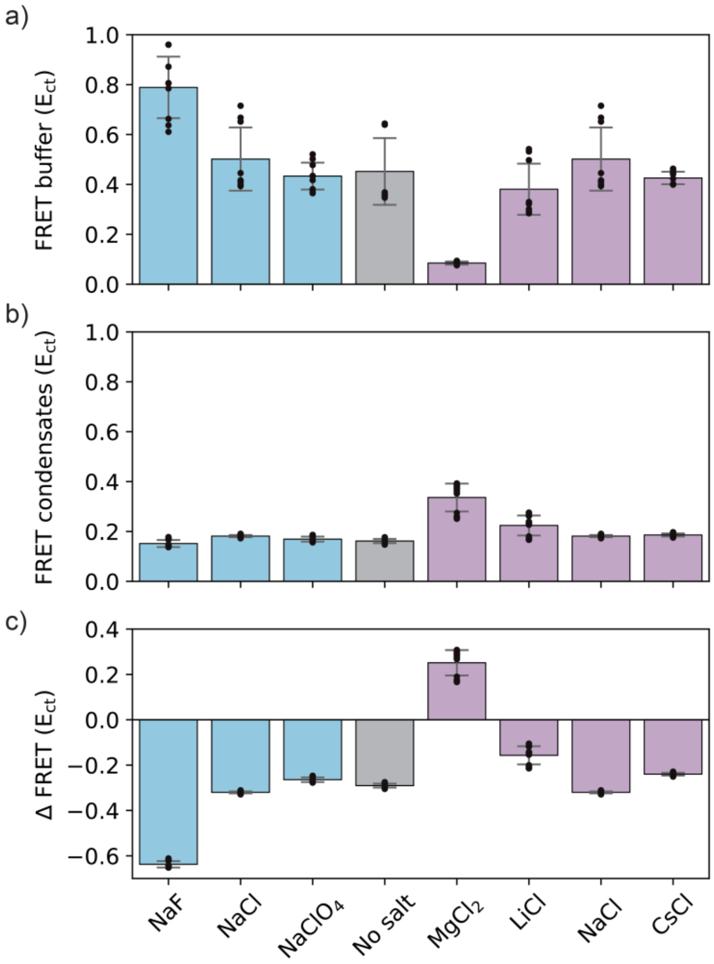

Supporting Figure 69: FRET intensities for the Cy3/Cy5-labelled DNA duplex in buffer and in protamine/ATP condensates. **a)** FRET efficiency in buffer, **b)** FRET efficiency in protamine/ATP condensates, **c)** difference in FRET efficiency between condensates and buffer (ΔFRET = FRET efficiency condensate – FRET efficiency buffer). A similar trend is observed as for RNA: kosmotropic anions stabilize the duplex in buffer, while the kosmotropic cations Mg^{2+} and Li^{+} destabilize the duplex. In condensates, the trend for the cations is reversed compared to buffer, similar to what we observed for RNA. The duplex stabilization by F^{-}, however, disappears for DNA inside condensates.

4. Extended Discussion

4.1. Rethinking charge-charge interactions

In this paper, we observed that charge-charge interactions between condensate components or between condensate components and salt ions follow the law of matching water affinities, resulting in strong binding between two chaotropes or two kosmotropes, but weak binding between ions with very different hydration strengths. These observations show that the entropic rearrangement of water molecules during ion complexation has an important contribution to charge-charge interactions, and therefore charge-charge interactions should not be considered as purely enthalpic, as is the naïve interpretation. This prompts us to rethink charge-charge interactions in the context of biomolecular condensates and may help to explain the thermodynamic properties of charge-based condensate formation.

Because all organic anions are chaotropic and all organic cations are kosmotropic, interaction between polymeric condensate components is inherently unfavorable in terms of ion hydration according to the LMWA, as it involves association of two ions with different water affinities. Interactions with typical counterions (chloride, sodium) are more favorable due to more similar water affinity. This matches well with observations from literature that complexation of polyelectrolytes and complex coacervation are typically entropy-driven[23–28] and sometimes even enthalpically unfavorable.[23,26,28]

Condensate formation is therefore often explained in terms of counterion release,[23–26] although counterion release has both an entropic and enthalpic factor, which will depend on the counterion involved. If counterion-polyelectrolyte side chain interactions are stronger than side chain-side chain interactions between polyelectrolytes, then strong counterion-side chain bonds have to be broken during polyelectrolyte complexation and counterion release, explaining the observed positive or mildly negative enthalpy. Interestingly, Yang *et al.* observed that the enthalpy of polyelectrolyte complex formation is highly dependent on the anionic counterion involved, with the kosmotropic (and thus weakly binding) acetate giving a ΔH_{PEC} of -6.0 kJ/mol, while the chaotropic (and thus strongly binding) perchlorate gives ΔH_{PEC} = 12.8 kJ/mol,[29] indicating that strong counterion-polyelectrolyte interactions (and associated changes in water-water and ion-water interactions) can make polyelectrolyte complexation enthalpically unfavorable, and is an important factor to consider in the thermodynamics of condensate formation. The observation that the presence of strongly binding ions can make condensate formation less thermodynamically favorable, matches with our observed lower T_{crit} for protamine/ATP with strongly binding ions in Figure 4i.

In addition to enthalpy, ion type is also expected to influence the entropy of condensate formation. Counterion release increases the entropy of the counterions, but also involves rearrangement of water molecules in the ion's hydration shell, and, therefore, the entropy associated with counterion release will depend on the hydration strength of the ion.[30]

Altogether, it is clear that charge-based condensate formation is not just driven by enthalpy-driven electrostatic interactions, and a more nuanced description of the process is required, involving the important role of counterions and hydration water. Our quantitative analysis of interaction strengths between ions and condensate components helps elucidate and refine the role of salt and counterions in condensate formation.

4.2. Comparison to other approaches to investigate protein-ion binding

We have interpreted our results through a stoichiometric binding model between the protamine/ATP and ions and explained the observations in terms of the "law of matching water affinities" (LMWA). Also other approaches have been used in literature to quantify and explain the interactions of ions with macromolecules, and we will use this discussion to put our work in context of two frequently used models: Kirkwood-Buff integrals and the surface-bulk partitioning model.

Kirkwood-Buff integrals (G_{ij}, based on the radial distribution function of molecule *j* in the volume around molecule *i*) and the preferential interaction coefficients derived from it are an excellent way to relate macroscopic thermodynamic properties to microscopic interactions between the water, protein and ions on the molecular level.[31] Preferential interaction coefficients express the relative preference of a protein to be surrounded by the ion or by water: $\frac{d\mu_{protein}}{d\mu_{ion}} = -c_{ion}(G_{protein,ion} - G_{protein,ion})$, and give the useful insight that changes in protein chemical potential upon addition of solutes are not caused by bulk changes to water structure ($G_{water-water}$),[32–34] but rather by water-mediated protein-solute interactions[35] as we investigate in this work.

The magnitude of Kirkwood-Buff integrals is influenced by factors such as excluded volume by the ion or other solute (especially for bulky solutes), "fluctuations" or variations in the number of molecules surrounding the molecule of interest, and specific (binding) interactions, although experimentally-derived Kirkwood-Buff integrals cannot discriminate between these effects.[32] When we relate this to our NMR binding curves, excluded volume and fluctuations are expected to scale linearly with solute concentration, and therefore give a linear change in chemical shift. Solvent fluctuations were in fact invoked by Trevitt et al.[3] to rationalize their observed dependence of the slope *A* of the linear part of Equation 1 on ion hardness. The fact that, in addition to the linear change, we also observe a non-linear change in $\Delta\delta$ indicates that there is specific binding between ions and proteins, similar to ligand binding. Our observed $K_{D,app}$'s of 150 mM and lower indicate that this binding is significantly stronger than the protein-water interaction; in the case of comparable protein-ion and protein-water interactions, site-specific binding models would yield $K_{D,app}$ values significantly greater than 1000 mM (e.g., $K_{D,app}$ values calculated for interactions between urea and proteins are in the order of 5000 mM).[36] Because we observe protein-ion interactions that are significantly stronger than the protein-water interactions, we believe it makes sense to describe the effect via a stoichiometric ligand-binding model.

What is important to note is that these interactions also appear to be site-specific, which general Kirkwood-Buff theory does not take into account. In this regard, our work builds upon pioneering work by the group of Paul Cremer, which gives a solid foundation that ions selectively interact with specific functional groups on proteins (see Okur et al. for an overview).[37] For example, hydrophobic moieties adjacent to electron-withdrawing groups (e.g., α-protons on the protein backbone) have a significant tendency to form contact pairs with chaotropic anions, while other nearby hydrophobic moieties such as hydrophobic side chains do not. For these reasons, we think that a site-specific binding model is a good model to explain our data, and gives insights that general Kirkwood-Buff theory could not.

In addition to our approach to measure site-specific ion binding to proteins, Kirkwood-Buff theory can be modified to sub-molecular resolution to gain insight into site-specific ion localization, as is done in the surface-bulk partitioning model as used by Record and coworkers,[38,39] by determining preferential interaction parameters for different regions on the protein. This is done either

experimentally through measuring salt-induced changes to the solubility or osmotic pressure of solutions of small model compounds that represent specific regions on the protein of interest, or through molecular dynamics simulations for which the radial distribution functions of ions around different parts of the protein are analyzed. As also described in Kar et al.,[40] the experimental approach has several pitfalls, especially concerning the extrapolation of data for small model compounds to large proteins or peptides, as surface-accessible areas are dependent on the protein environment and the strength of ion-macromolecule interactions depends quite significantly on the macromolecule length.[4] The preferential interaction potentials obtained from model compounds may therefore differ significantly from what is experienced by the macromolecule. Nevertheless, the bulk-partitioning model gives a useful estimate of the relative preference of the protein site to interact with the ion or with water, especially when determined by molecular dynamics simulations, as this can actually report the individual contributions of $G_{protein,ion}$ and $G_{protein,water}$.

The gap in Kirkwood-Buff theory and the associated surface-bulk partitioning model is, however, that they do not explain the reason for the preferential interaction coefficients, as they just report on the number of ions or water molecules around the protein (site). Such an assumption-free model clearly has its advantages, but it does not give us more insight into the mechanisms of site-specific binding of ions to proteins. This is where LMWA comes in to explain the balance between protein-water, ion-water and water-water interactions that gives rise to specific binding between ions and proteins. According to LMWA, while chaotrope-chaotrope interactions seem to be driven by protein-water, ion-water and water-water interactions (i.e., driven by differences in hydration enthalpy), kosmotrope-kosmotrope interactions seem to be driven by strong protein-ion interactions between the high-charge-density (hard) kosmotropes (i.e., binding energy).

In this respect, it seems that Kirkwood-Buff theory and specific ion binding following LMWA are not mutually exclusive. Where Kirkwood-Buff theory gives insight into the effect of ion addition on the relative interaction preferences of the total ensemble of molecules in the system, and therefore the driving forces for changes in macroscopic properties, site-specific ion binding following LMWA can explain the cause of the ion-protein interactions.

5. Data repository

Raw data can be found on the Radboud Data repository (DOI:10.34973/bmt1-aa56).

6. References Supporting Information

(1) Abbas, M.; Lipiński, W. P.; Nakashima, K. K.; Huck, W. T. S.; Spruijt, E. A Short Peptide Synthon for Liquid–Liquid Phase Separation. *Nat. Chem.* **2021**, *13* (11), 1046–1054. https://doi.org/10.1038/s41557-021-00788-x.

(2) Rembert, K. B.; Paterová, J.; Heyda, J.; Hilty, C.; Jungwirth, P.; Cremer, P. S. Molecular Mechanisms of Ion-Specific Effects on Proteins. *J. Am. Chem. Soc.* **2012**, *134* (24), 10039–10046. https://doi.org/10.1021/ja301297g.

(3) Trevitt, C. R.; Yashwanth Kumar, D. R.; Fowler, N. J.; Williamson, M. P. Interactions between the Protein Barnase and Co-Solutes Studied by NMR. *Commun. Chem.* **2024**, *7* (1), 1–15. https://doi.org/10.1038/s42004-024-01127-0.

(4) Rogers, B. A.; Okur, H. I.; Yan, C.; Yang, T.; Heyda, J.; Cremer, P. S. Weakly Hydrated Anions Bind to Polymers but Not Monomers in Aqueous Solutions. *Nat. Chem.* **2022**, *14* (1), 40–45. https://doi.org/10.1038/s41557-021-00805-z.

(5) Paterová, J.; Rembert, K. B.; Heyda, J.; Kurra, Y.; Okur, H. I.; Liu, W. R.; Hilty, C.; Cremer, P. S.; Jungwirth, P. Reversal of the Hofmeister Series: Specific Ion Effects on Peptides. *J. Phys. Chem. B* **2013**, *117* (27), 8150–8158. https://doi.org/10.1021/jp405683s.

(6) Cho, Y.; Zhang, Y.; Christensen, T.; Sagle, L. B.; Chilkoti, A.; Cremer, P. S. Effects of Hofmeister Anions on the Phase Transition Temperature of Elastin-like Polypeptides. *J. Phys. Chem. B* **2008**, *112* (44), 13765–13771. https://doi.org/10.1021/jp8062977.

(7) Abraham, M. J.; Murtola, T.; Schulz, R.; Páll, S.; Smith, J. C.; Hess, B.; Lindah, E. Gromacs: High Performance Molecular Simulations through Multi-Level Parallelism from Laptops to Supercomputers. *SoftwareX* **2015**, *1–2*, 19–25. https://doi.org/10.1016/j.softx.2015.06.001.

(8) Piana, S.; Robustelli, P.; Tan, D.; Chen, S.; Shaw, D. E. Development of a Force Field for the Simulation of Single-Chain Proteins and Protein-Protein Complexes. *J. Chem. Theory Comput.* **2020**, *16* (4), 2494–2507. https://doi.org/10.1021/acs.jctc.9b00251.

(9) Li, P.; Song, L. F.; Merz, K. M. Systematic Parameterization of Monovalent Ions Employing the Nonbonded Model. *J. Chem. Theory Comput.* **2015**, *11* (4), 1645–1657. https://doi.org/10.1021/ct500918t.

(10) Bussi, G.; Donadio, D.; Parrinello, M. Canonical Sampling through Velocity Rescaling. *J. Chem. Phys.* **2007**, *126* (1), 14101. https://doi.org/10.1063/1.2408420.

(11) Parrinello, M.; Rahman, A. Polymorphic Transitions in Single Crystals: A New Molecular Dynamics Method. *J. Appl. Phys.* **1981**, *52* (12), 7182–7190. https://doi.org/10.1063/1.328693.

(12) Breßler, I.; Kohlbrecher, J.; Thünemann, A. F. SASfit: A Tool for Small-Angle Scattering Data Analysis Using a Library of Analytical Expressions. *J. Appl. Crystallogr.* **2015**, *48* (5), 1587–1598. https://doi.org/10.1107/S1600576715016544.

(13) Hammouda, B. Small-Angle Scattering from Branched Polymers. *Macromol. Theory Simulations* **2012**, *21* (6), 372–381. https://doi.org/10.1002/mats.201100111.

(14) Silva, E. R.; Listik, E.; Han, S. W.; Alves, W. A.; Soares, B. M.; Reza, M.; Ruokolainen, J.; Hamley, I. W. Sequence Length Dependence in Arginine/Phenylalanine Oligopeptides: Implications for Self-Assembly and Cytotoxicity. *Biophys. Chem.* **2018**, *233*, 1–12. https://doi.org/10.1016/j.bpc.2017.11.005.

(15) De Mello, L. R.; Hamley, I. W.; Castelletto, V.; Garcia, B. B. M.; Han, S. W.; De Oliveira, C. L. P.; Da Silva, E. R. Nanoscopic Structure of Complexes Formed between DNA and the Cell-Penetrating Peptide Penetratin. *J. Phys. Chem. B* **2019**, *123* (42), 8861–8871. https://doi.org/10.1021/acs.jpcb.9b05512.

(16) Smokers, I. B. A.; Spruijt, E. Quantification of Biomolecular Condensate Volume Reveals Network Swelling and Dissolution Regimes during Phase Transition. *Biomacromolecules* **2024**, *26* (1), 363–373. https://doi.org/10.1021/acs.biomac.4c01201.

(17) Jones, G.; Dole, M. The Viscosity of Aqueous Solutions of Strong Electrolytes with Special Reference to Barium Chloride. *J. Am. Chem. Soc.* **1929**, *51* (10), 2950–2964. https://doi.org/10.1021/ja01385a012.

(18) Collins, K. D. The Behavior of Ions in Water Is Controlled by Their Water Affinity. *Q. Rev. Biophys.* **2019**, *52*, e11. https://doi.org/10.1017/S0033583519000106.

(19) Robinson, J. B.; Strottmann, J. M.; Stellwagen, E. Prediction of Neutral Salt Elution Profiles for Affinity Chromatography. *Proc. Natl. Acad. Sci. U. S. A.* **1981**, *78* (4), 2287–2291. https://doi.org/10.1073/pnas.78.4.2287.

(20) Petrášek, Z.; Schwille, P. Precise Measurement of Diffusion Coefficients Using Scanning Fluorescence Correlation Spectroscopy. *Biophys. J.* **2008**, *94* (4), 1437–1448. https://doi.org/10.1529/biophysj.107.108811.

(21) Schrimpf, W.; Barth, A.; Hendrix, J.; Lamb, D. C. PAM: A Framework for Integrated Analysis of Imaging, Single-Molecule, and Ensemble Fluorescence Data. *Biophys. J.* **2018**, *114* (7), 1518–1528. https://doi.org/10.1016/j.bpj.2018.02.035.

(22) Van Haren, M. H. I.; Visser, B. S.; Spruijt, E. Probing the Surface Charge of Condensates Using Microelectrophoresis. *Nat. Commun.* **2024**, *15* (1), 1–10. https://doi.org/10.1038/s41467-024-47885-2.

(23) Chang, L. W.; Lytle, T. K.; Radhakrishna, M.; Madinya, J. J.; Vélez, J.; Sing, C. E.; Perry, S. L. Sequence and Entropy-Based Control of Complex Coacervates. *Nat. Commun.* **2017**, *8* (1), 1–8. https://doi.org/10.1038/s41467-017-01249-1.

(24) Kayitmazer, A. B. Thermodynamics of Complex Coacervation. *Adv. Colloid Interface Sci.* **2017**, *239*, 169–177. https://doi.org/10.1016/j.cis.2016.07.006.

(25) Chen, S.; Wang, Z. G. Driving Force and Pathway in Polyelectrolyte Complex Coacervation. *Proc. Natl. Acad. Sci. U. S. A.* **2022**, *119* (36), e2209975119. https://doi.org/10.1073/pnas.2209975119.

(26) Priftis, D.; Laugel, N.; Tirrell, M. Thermodynamic Characterization of Polypeptide Complex Coacervation. *Langmuir* **2012**, *28* (45), 15947–15957. https://doi.org/10.1021/la302729r.

(27) Fu, J.; Schlenoff, J. B. Driving Forces for Oppositely Charged Polyion Association in Aqueous Solutions: Enthalpic, Entropic, but Not Electrostatic. *J. Am. Chem. Soc.* **2016**, *138* (3), 980–990. https://doi.org/10.1021/jacs.5b11878.

(28) Chowdhury, A.; Borgia, A.; Ghosh, S.; Sottini, A.; Mitra, S.; Eapen, R. S.; Borgia, M. B.; Yang, T.; Galvanetto, N.; Ivanović, M. T.; Łukijańczuk, P.; Zhu, R.; Nettels, D.; Kundagrami, A.; Schuler, B. Driving Forces of the Complex Formation between Highly Charged Disordered Proteins. *Proc. Natl. Acad. Sci. U. S. A.* **2023**, *120* (41). https://doi.org/10.1073/pnas.2304036120.

(29) Yang, M.; Digby, Z. A.; Schlenoff, J. B. Precision Doping of Polyelectrolyte Complexes: Insight on the Role of Ions. *Macromolecules* **2020**, *53* (13), 5465–5474.

https://doi.org/10.1021/acs.macromol.0c00965.

(30) Shen, Y.; Li, S.; Jiang, J.; Sun, F.; Zhao, Y.; Qiao, F.; Qin, B. Ions Effect on Tunable Coacervate and Its Relevance to the Hofmeister Series. *Colloids Surfaces A Physicochem. Eng. Asp.* **2024**, *702*, 134597. https://doi.org/10.1016/j.colsurfa.2024.134597.

(31) Kirkwood, J. G.; Buff, F. P. The Statistical Mechanical Theory of Solutions. I. *J. Chem. Phys.* **1951**, *19* (6), 774–777. https://doi.org/10.1063/1.1748352.

(32) Abbott, S. Solubility Science: Principles and Practice https://www.stevenabbott.co.uk/practical-solubility/the-book.php (accessed Jun 29, 2025).

(33) Booth, J. J.; Abbott, S.; Shimizu, S. Mechanism of Hydrophobic Drug Solubilization by Small Molecule Hydrotropes. *J. Phys. Chem. B* **2012**, *116* (51), 14915–14921. https://doi.org/10.1021/jp309819r.

(34) Booth, J. J.; Omar, M.; Abbott, S.; Shimizu, S. Hydrotrope Accumulation around the Drug: The Driving Force for Solubilization and Minimum Hydrotrope Concentration for Nicotinamide and Urea. *Phys. Chem. Chem. Phys.* **2015**, *17* (12), 8028–8037. https://doi.org/10.1039/c4cp05414h.

(35) Shimizu, S.; McLaren, W. M.; Matubayasi, N. The Hofmeister Series and Protein-Salt Interactions. *J. Chem. Phys.* **2006**, *124* (23), 234905. https://doi.org/10.1063/1.2206174.

(36) Schellman, J. A. The Thermodynamics of Solvent Exchange. *Biopolymers* **1994**, *34* (8), 1015–1026. https://doi.org/10.1002/bip.360340805.

(37) Okur, H. I.; Hladílková, J.; Rembert, K. B.; Cho, Y.; Heyda, J.; Dzubiella, J.; Cremer, P. S.; Jungwirth, P. Beyond the Hofmeister Series: Ion-Specific Effects on Proteins and Their Biological Functions. *J. Phys. Chem. B* **2017**, *121* (9), 1997–2014. https://doi.org/10.1021/acs.jpcb.6b10797.

(38) Pegram, L. M.; Record, M. T. Thermodynamic Origin of Hofmeister Ion Effects. *J. Phys. Chem. B* **2008**, *112* (31), 9428–9436. https://doi.org/10.1021/jp800816a.

(39) Kozlov, A. G.; Cheng, X.; Zhang, H.; Shinn, M. K.; Weiland, E.; Nguyen, B.; Shkel, I. A.; Zytkiewicz, E.; Finkelstein, I. J.; Record, M. T.; Lohman, T. M. How Glutamate Promotes Liquid-Liquid Phase Separation and DNA Binding Cooperativity of E. Coli SSB Protein. *J. Mol. Biol.* **2022**, *434* (9), 167562. https://doi.org/10.1016/j.jmb.2022.167562.

(40) Kar, M.; Vogel, L. T.; Chauhan, G.; Felekyan, S.; Ausserwöger, H.; Welsh, T. J.; Dar, F.; Kamath, A. R.; Knowles, T. P. J.; Hyman, A. A.; Seidel, C. A. M.; Pappu, R. V. Solutes Unmask Differences in Clustering versus Phase Separation of FET Proteins. *Nat. Commun.* **2024**, *15* (1), 1–21. https://doi.org/10.1038/s41467-024-48775-3.
